# Supplementary material for: Structure-Activity Relationship Studies Towards Analogues of Pleconaril as Novel Enterovirus-D68 Capsid-Targeting Antivirals
Source: bioRxiv. 2025 Aug 12:2025.08.08.668114. Preprint. [Version 2] doi: 10.1101/2025.08.08.668114 (PMC12363871; doi:10.1101/2025.08.08.668114)
Supplement: Supplement 1 [file media-1.pdf]

Supporting Information: Structure-Activity Relationship Studies Towards Analogues of Pleconaril as Novel Enterovirus-D68 Capsid-Targeting Antivirals

David L. Cousins,[*,†,⊥] Ed J. Griffen,[†,⊥] Jessica Stacey,[†,⊥] Alpha A. Lee,[*,‡,⊥] Yuliia Filimonova,[¶] Anton Hlavin,[¶] Yuliia Holota,[¶] Roman Khmil,[¶] Mykyta Kordubailo,[¶] Oleksii Kostinov,[¶] Dmytro Lesyk,[¶] Ivan Logvinenko,[¶] Mariia Lototska,[¶] Viacheslav Lysenko,[¶] Anna Pashchenko,[¶] Mariia Pavlichenko,[¶] Anzhela Rodnichenko,[¶] Anton Tkachenko,[¶] Brett L. Hurst,[§] Justin G. Julander,[§] Hong Wang,[§] Rebecca Pearl,[‖,⊥] Jared Benjamin,[‖,⊥] Randy Diaz-Tapia,[‖,⊥] Mary E. Gordon,[‖,⊥] Randy A. Albrecht,[‖,⊥] and Kris White[‖,⊥]

†*MedChemica Consultancy Ltd, Macclesfield, Cheshire, SK11 6DU, UK. https://medchemica.com/*

‡*PostEra, 1 Broadway, 14th floor, Cambridge, MA 02142, USA. https://postera.ai/*

¶*Enamine Ltd., Winston Churchill Street 78, Kyiv, 02094 Ukraine. https://enamine.net/*

§*Institute for Antiviral Research, Utah State University, Logan, UT 84322, USA. https://caas.usu.edu/iar/*

‖*Department of Microbiology, Global Health and Emerging Pathogens Institute, Icahn School of Medicine at Mount Sinai, New York, NY, USA.*

https://icahn.mssm.edu/research/global-health

⊥*The ASAP Discovery Consortium, https://asapdiscovery.org/*

E-mail: david.cousins@medchemica.com; alpha.lee@postera.ai

Contents

Synthetic Chemistry and Characterisation Data **S-5**

General Laboratory Conditions S-7

Synthesis of Intermediate **9** S-9

Synthesis of Compound **2** S-10

Synthesis of Compound **22** S-11

Synthesis of Compound **23** S-14

Synthesis of Compound **24** S-17

Synthesis of Compounds **25** and **26** S-20

Synthesis of Compound **27** S-23

Synthesis of Compound **28** S-27

Synthesis of Compound **29** S-27

Synthesis of Compound **30** S-28

Synthesis of Compound **31** S-28

Synthesis of Compound **32** S-32

Synthesis of Compounds **33** and **34** S-36

Synthesis of Compound **35** S-39

Synthesis of Compound **36** S-41

Synthesis of Compound **37** S-41

Synthesis of Compound **38** S-43

Synthesis of Compound **39** S-45

Synthesis of Compound **40** S-47

Synthesis of Compound **41** S-48

Synthesis of Compound **42** S-49

Synthesis of Compound **43** S-49

Synthesis of Compound **44** S-50

Synthesis of Compound **45** S-51

Synthesis of Compound **46** S-53

Synthesis of Compound **47** S-54

Synthesis of Compound **48** S-57

Synthesis of Compound **49** S-60

Synthesis of Compounds **50** and **51** S-61

General Procedure A:

Parallel Synthesis of Phenol Ethers by Mitsunobu Coupling S-64

Synthesis of Compound **52** S-64

Synthesis of Compound **53** S-64

Synthesis of Compound **54** S-65

Synthesis of Compound **55** S-65

Synthesis of Compound **56** S-66

Synthesis of Compound **S-57** (BTA-188) S-67

Synthesis of Compound **S-59** (NLD-22) S-69

Synthesis of Compound **S-60** (OBR-5-340) S-73

Methodology for Antiviral and ADME Assays **S-76**

EV-D68 Antiviral Screening Assay in RD Cells S-76

EV Panel Antiviral Screening Assays S-77

ADME Assays - LogD S-77

ADME Assays - KSOL S-77

ADME Assays - MDCK Permeability S-78

ADME Assays - Human and Mouse Microsomal Stability S-78

Antiviral and ADME Profiling of Published Antivirals **S-80**

Published EV Capsid Structural Data **S-81**

List of Ligand-Bound Crystal and Cryo-EM Structures of VP1 . . . . S-81

3D Overlays S-84

Synthetic Chemistry and Characterisation Data

Abbreviations

**ACN**	Acetonitrile
**AcOH**	Acetic acid
**ADDP**	1,1-(Azodicarbonyl)dipiperidine
**Ar**	Argon
**CataCXium A**	Di(1-adamantyl)-n-butyl phosphine
**CDI**	1,1-Carbonyldiimidazole
**DCM**	Dichloromethane
**DEAD**	Diethyl azodicarboxylate
**DIAD**	Diisopropyl azodicarboxylate
**DIPEA**	N,N-Diisopropylethylamine
**DMAA**	Dimethylacetamide
**DMF**	Dimethylformamide
**DMSO**	Dimethyl sulfoxide
**ESI**	Electrospray ionisation
**EtOH**	Ethanol
**EtPh**	Ethylbenzene
**Et_2O**	Diethyl ether
**Et_3N**	Triethylamine
**FCC**	Flash column chromatography
**g**	Gram
**HATU**	O-(7-azabenzotriazol-1-yl)-N,N,N,N-tetramethyluronium hexafluorophosphate
**h**	Hour
**^1H**	Proton
**HCl**	Hydrochloric acid
**HPLC**	High performance liquid chromatography
**HSQC**	Heteronuclear single quantum correlation
**Hz**	Hertz
**K_3PO_4**	Potassium phosphate tribasic
**LCMS**	Liquid chromatography-mass spectrometry
**M**	Molar
**MeOH**	Methanol

**mg**	Milligram(s)
**$MgSO_4$**	Magnesium sulfate
**min**	Minute(s)
**mL**	Milliliter(s)
μ**M**	Microliter(s)
**mm**	Millimeter(s)
**mmol**	Millimole(s)
μ**mol**	Micromole(s)
**NaCl**	Sodium chloride
**NaH**	NaH
**$NaHCO_3$**	Sodium bicarbonate
**NaOH**	Sodium hydroxide
**Na_2SO_4**	Sodium sulfate
**NH_4Cl**	Ammonium chloride
**NH_4OH**	Ammonium hydroxide
**NOESY**	Nuclear Overhauser effect spectroscopy
**PMB**	4-Methoxybenzyl
**PSCBH**	Polymer-supported cyano borohydride
**quant**	Quantitative
**rt**	Room temperature
**RT**	Retention time
**SEM-Cl**	2-(Trimethylsilyl)ethoxymethyl chloride
**SFC**	Supercritical fluid chromatography
**TFAA**	Trifluoroacetic anhydride
**TFA**	Trifluoroacetic acid
**THF**	Tetrahydrofuran
**TLC**	Thin layer chromatography
**UPLC-MS**	Ultra performance liquid chromatography-mass spectrometry
% **wt.**	% weight
**XPhos Pd G3**	2-dicyclohexylphosphino-2,4,6-triisopropyl-1,1-biphenyl)[2-(2-amino-1,1-biphenyl)]palladium(II) methanesulfonate

General Laboratory Conditions

1 (pleconaril) was purchased from MedChemExpress, **3** (vapendavir) and **S-58** (pirodavir) were purchased from AA Blocks Inc., purchased compounds were used as obtained without further purification. All other starting materials were obtained from commercial suppliers and used without further purification unless stated otherwise.

All evaporations were carried out in vacuo with a rotary evaporator. Analytical samples were dried in vacuo (1-5 mmHg) at rt. All references to brine refer to a saturated aqueous solution of NaCl. Unless otherwise indicated, all temperatures are expressed in °C (degrees Centigrade). Most of the reactions were monitored by UPLC-MS (Shimadzu LCMS-2020 Single Quadrupole Liquid Chromatograph Mass Spectrometer or Waters ACQUITY UPLC I-Class PLUS System with Waters SQ Detector 2) or thin-layer chromatography on 0.25mm Merck silica gel plates (60F-254), visualized with UV light. Flash column chromatography was performed on prepacked silica gel cartridges (50 μM, puriFlash®), Biotage®Sfär KPAmino D (Duo 50 μM, 28-110 g) and puriFlash®RP-AQ or C18-HP (15 μM, 120-330 g). Preparative TLC was performed on silica gel plates glass-backed, 1000 μM, Analtech. Nuclear magnetic resonance (NMR) spectroscopy of samples was carried out on either a Bruker AVANCE DRX 500 or a Varian UNITYplus 400. Proton NMR spectra were recorded at 400 MHz or 500 MHz. 13C NMR spectra were recorded at 100 MHz or 125 MHz. Chemical shifts (δ) are given in parts per million (ppm) and are listed upfield with tetramethylsilane as a reference. Peaks are described as singlets (s), doublets (d), triplets (t), quartets (q), quintets (quint) multiplets (m) and broad (br.). All assignments of NMR spectra were based on 1D NMR data. Raw NMR data (FID) can be provided on request.

LCMS Method Details

Column Agilent Poroshell 120 SB-C18 4.6x30mm 2.7 μm

Column Temperature 60°C

Mobile phase A: water (0.1% formic acid), B: acetonitrile (0.1% formic acid)

Flow Rate 3 ml min^{-1}

Gradient 0.01 min – 1% B, 1.5 min – 100% B, 1.73 min – 100% B

MS Ionisation Mode ESI

MS Scan Range 83 – 600 m/z or 83 – 1000 m/z

UV Detection Wavelengths 215 nm, 254 nm and 280 nm

Synthesis of key building block: 2-methyl-4-[5-(trifluoromethyl)-1,2,4-oxadiazol-3-yl]phenol (Compound 9)

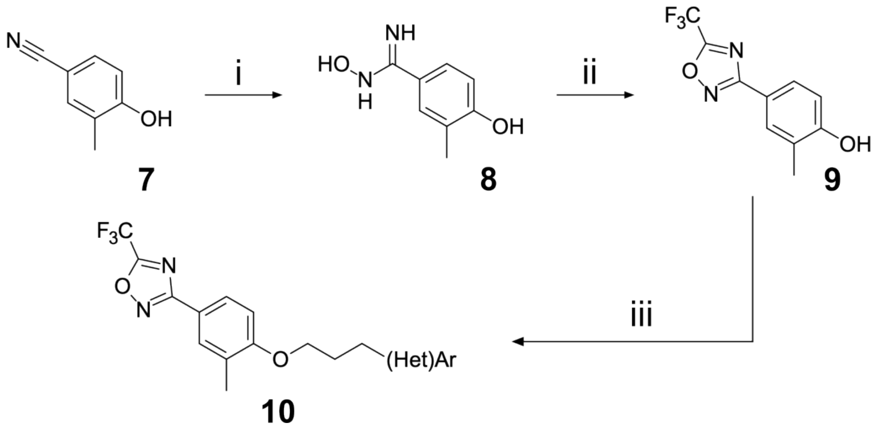

Scheme S1: Reagents and conditions: (i) hydroxylamine hydrochloride, K_2CO_3, EtOH (absolute), reflux, 16h (ii) TFAA, pyridine, 80 °C, 2h (iii) 3-(hetero)arylpropyl alcohol, PPh_3, DEAD, THF, RT, 12h

Step i: Data for N',4-dihydroxy-3-methyl-benzamidine (Compound 8)

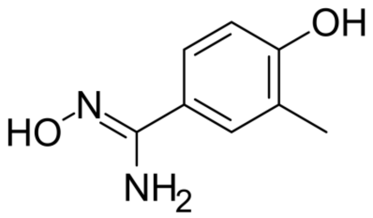

K_2CO_3 (51.9 g, 376 mmol) and hydroxylamine hydrochloride (26.1 g, 376 mmol) were added to a stirred solution of 4-hydroxy-3-methyl-benzonitrile (10.0 g, 75.1 mmol) in EtOH (284 mL) at room temperature. The resulting mixture was stirred at reflux overnight. The reaction mixture was filtered hot. The filter cake was washed with hot ethanol (2×100 mL). The combined filtrates were concentrated under reduced pressure to afford N',4-dihydroxy-3-methyl-benzamidine (16 g, 72.21 mmol, 96.15% yield, 75% purity) as a brown solid which was used in the next step without further purification. LCMS(ESI): $[M+H]^+$ m/z: calcd 167.08; found 167.2.

Step ii: Data for 2-methyl-4-[5-(trifluoromethyl)-1,2,4-oxadiazol-3-yl]phenol (Compound 9)

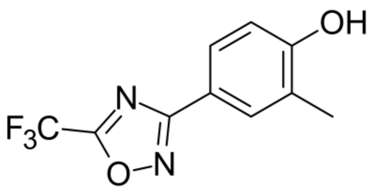

TFAA (30.3 g, 144 mmol, 20 mL) was added dropwise to a stirred solution of N',4-dihydroxy-3-methyl-benzamidine (16.0 g, 72.2 mmol) in pyridine (201 mL) at 85 °C. The resulting mixture was stirred at 85 °C for 1 hr. The reaction mixture was cooled to room temperature and concentrated under reduced pressure. The residue was partitioned between EtOAc (300 mL) and water (100 mL). The organic layer was separated, washed with water (3×100 mL) and brine (100 mL). The combined organic layers were dried over anhydrous sodium sulfate and concentrated under reduced pressure. The residue was subjected to flash column chromatography (SiO_2, Hex-EtOAc 4:1) to afford 2-methyl-4-[5-(trifluoromethyl)-1,2,4-oxadiazol-3-yl]phenol (9.50 g, 38.9 mmol, 53.9% yield) as a light-yellow solid. ^{1}H NMR (500 MHz, $CDCl_3$) δ_H 2.34 (s, 3H), 5.26 (s, 1H), 6.91 (d, 1H), 7.82 (d, 1H), 7.92 (s, 1H). LCMS(ESI): $[M+H]^+$ m/z: calcd 245.06; found 245.0.

Synthesis of N,N-dimethyl-5-[3-[2-methyl-4-[5-(trifluoromethyl)-1,2,4-oxadiazol-3-yl]phenoxy]propyl]isoxazole-3-carboxamide (Compound 2)

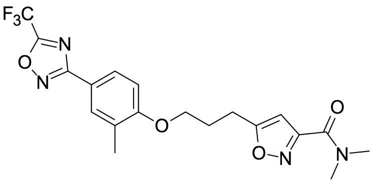

5-(3-Hydroxypropyl)-N,N-dimethyl-isoxazole-3-carboxamide (40.6 mg, 205 μmol) was mixed with 2-methyl-4-[5-(trifluoromethyl)-1,2,4-oxadiazol-3-yl]phenol (50.0 mg, 205 μmol) and PPh_3 (80.6 mg, 307 μmol) in dry THF (3.0 mL), after that DEAD (53.5 mg, 307 μmol) was added dropwise to the solution at 0 °C. The resulting mixture was allowed to warm up to room temper-

ature and stirred overnight. The reaction mixture was subjected to HPLC (0-1-6 min., 40-40-80% water – ACN, flow: 60 mL/min, column: XBridge C18 OBD 100×30 mm, 5 μm) to afford N,N-dimethyl-5-[3-[2-methyl-4-[5-(trifluoromethyl)-1,2,4-oxadiazol-3-yl]phenoxy]propyl]isoxazole-3-carboxamide (53.3 mg, 126 μmol, 61.3% yield) as a white solid. ^{1}H NMR (500 MHz, dmso) δ_H 2.14 – 2.20 (m, 2H), 2.22 (s, 3H), 2.98 (s, 3H), 3.01 (t, 2H), 3.04 (s, 3H), 4.14 (t, 2H), 6.53 (s, 1H), 7.13 (d, 1H), 7.84 (d, 1H), 7.86 (dd, 1H). LCMS(ESI): $[M+H]^+$ m/z: calcd 425.16; found 425.0.

Synthesis of N,N-dimethyl-5-[2-[[2-methyl-4-[5-(trifluoromethyl) - 1,2,4-oxadiazol-3-yl]phenyl]methoxy]ethyl]isoxazole-3-carboxamide (Compound 22)

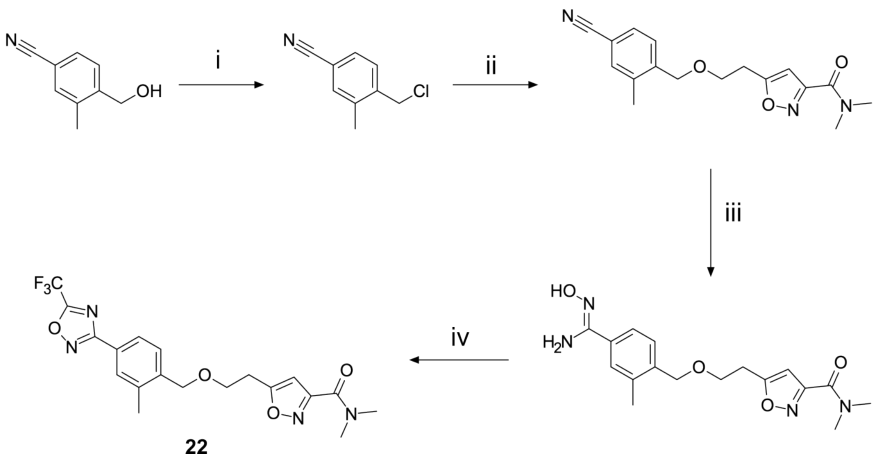

22

Scheme S2: Reagents and conditions: (i) $SOCl_2$, $CHCl_3$, 50 °C, 3h (ii) 5-(2-hydroxyethyl)-N,N-dimethyl-isoxazole-3-carboxamide, NaH, DMF, 0 °C to RT, 16h (iii) hydroxylamine hydrochloride, Na_2CO_3, H_2O/MeOH, 60 °C, 16h (iv) TFAA, pyridine, THF, 0 °C to 60 °C, 16h

Step i: Data for 4-(chloromethyl)-3-methyl-benzonitrile)

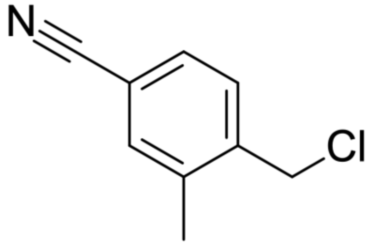

$SOCl_2$ (606 mg, 5.10 mmol, 372 μL) was added to a stirred solution of 4-(hydroxymethyl)-3-methyl-benzonitrile (500 mg, 3.40 mmol) in $CHCl_3$ (5.0 mL) at room temperature. The resulting mixture was stirred at 50 °C for 3 hr. The reaction mixture was cooled to room temperature and concentrated under reduced pressure to afford 4-(chloromethyl)-3-methyl-benzonitrile (560 mg, 3.38 mmol, 99.5% yield) as a yellow solid which was used in the next step without further purification. ^{1}H NMR (400 MHz, CDCl3) δ_H 2.47 (s, 3H), 4.60 (s, 2H), 7.41 – 7.54 (m, 3H).

Step ii: Data for 5-[2-[(4-cyano-2-methyl-phenyl)methoxy]ethyl]-N,N-dimethyl-isoxazole-3-carboxamide

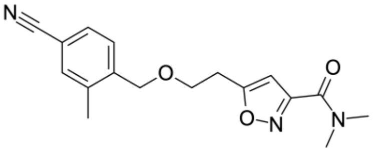

NaH (15.9 mg, 664 μmol, 60% dispersion in mineral oil) was added at 0 °C to a solution of 5-(2-hydroxyethyl)-N,N-dimethyl-isoxazole-3-carboxamide (111 mg, 604 μmol) in DMF (3.0 mL). The resulting mixture was stirred at 0 °C for 30 min. 4-(Chloromethyl)-3-methyl-benzonitrile (100 mg, 604 μmol) was added at 0 °C to the mixture. The resulting mixture was stirred at room temperature for 16 hr. The reaction mixture was concentrated under reduced pressure. The residue was subjected to reverse phase HPLC (0-1.3-6.3 min., 15-15-40% water – ACN, flow: 30 mL/min, column: Chromatorex 18 SMB100-5T 100×19 mm, 5 μm) to afford 5-[2-[(4-cyano-2-methyl-phenyl)methoxy]ethyl]-N,N-dimethyl-isoxazole-3-carboxamide (7.00 mg, 22.3 μmol, 3.70% yield) as a yellow oil. LCMS(ESI): $[M+H]^+$ m/z: calcd 314.17; found 314.2.

Step iii: Data for 5-[2-[[4-(N-hydroxycarbamimidoyl)-2-methyl-phenyl]methox

N,N-dimethyl-isoxazole-3-carboxamide

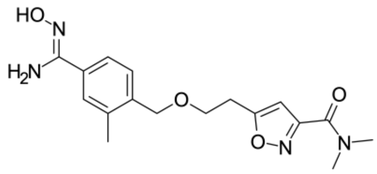

Hydroxylamine hydrochloride (4.66 mg, 67.0 μmol) and Na_2CO_3 (2.37 mg, 22.3 μmol) were added at room temperature to a stirred solution of 5-[2-[(4-cyano-2-methyl-phenyl)methoxy]ethyl]-N,N-dimethyl-isoxazole-3- carboxamide (7.00 mg, 22.3 μmol) in water (0.3 mL) and EtOH (0.5 mL). The resulting mixture was stirred at 60 °C for 16 hr. The reaction mixture was cooled to room temperature and concentrated under reduced pressure. The residue was redissolved in DCM (5 mL) and washed with water (3×4 mL). The organic layer was separated, dried over anhydrous sodium sulfate and concentrated under reduced pressure to afford 5-[2-[[4-(N-hydroxycarbamimidoyl)-2-methyl-phenyl]methoxy]ethyl]-N,N-dimethyl-isoxazole-3-carboxamide (7.00 mg, 20.2 μmol, 90.5% yield) as a white powder which was used in the next step without further purification. LCMS(ESI): $[M+H]^+$ m/z: calcd 347.17; found 347.2.

Step iv: Data for N,N-dimethyl-5-[2-[[2-methyl-4-[5-(trifluoromethyl)-1,2,4-oxadiazol-3-yl]phenyl]methoxy]ethyl]isoxazole-3-carboxamide (Compound 22)

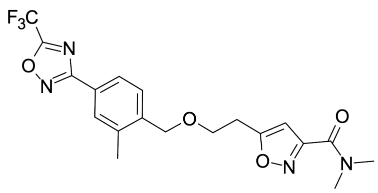

Pyridine (5.70 μL) and TFAA (10.6 mg, 50.5 μmol, 7.02 μL) were added to a stirred solution of 5-[2-[[4-(N-hydroxycarbamimidoyl)-2-methyl-phenyl]methoxy]ethyl]-N,N-dimethyl-isoxazole-3-carboxamide (7.00 mg, 20.2 μmol) in THF (1.0 mL) at 0 °C. The resulting mixture was stirred at 60 °C for 16 hr. The reaction mixture was cooled to room temperature and concentrated under reduced pressure. The residue was subjected to reverse phase HPLC (0-1.3-5.3 min., 45-45-65% water – ACN, flow: 30 mL/min, column: Chromatorex 18 SMB100-5T 100×19 mm, 5 μm) to afford N,N-dimethyl-5-[2-[[2-methyl-4-[5-(trifluoromethyl)-1,2,4-oxadiazol-3-yl]phenyl]methoxy]ethyl]isoxazole-3-carboxamide (3.50 mg, 8.25 μmol, 40.8% yield) as a yel-

low oil. ^{1}H NMR (500 MHz, CD_3OD) δ_H 2.36 (s, 3H), 3.10 (s, 3H), 3.16 (t, 2H), 3.18 (s, 3H), 3.89 (t, 2H), 4.63 (s, 2H), 6.41 (s, 1H), 7.49 (d, 1H), 7.87 – 7.93 (m, 2H). LCMS(ESI): $[M+H]^+$ m/z: calcd 425.16; found 425.2.

Synthesis of N,N-dimethyl-5-[2-[2-methyl-4-[5-(trifluoromethyl)-1,2,4-oxadiazol-3-yl]phenyl]ethoxymethyl]isoxazole-3-carboxamide (Compound 23)

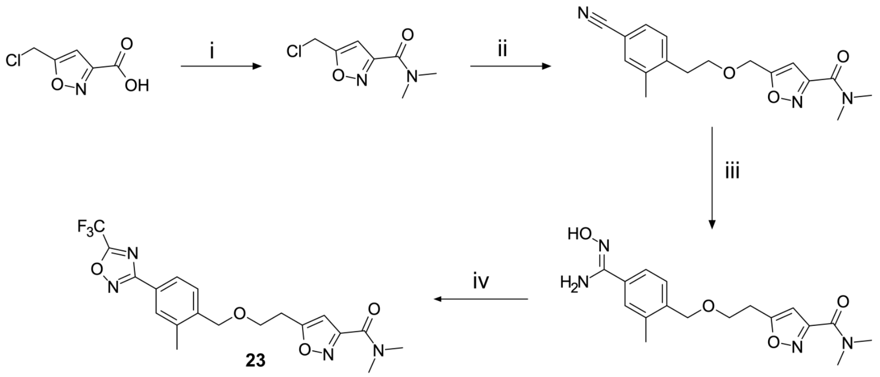

Scheme S3: Reagents and conditions: (i) Me_2NH, $(COCl)_2$, DIPEA, THF / DCM, 0 °C to RT, 16h (ii) 4-(2-hydroxyethyl)-3-methyl-benzonitrile, tetrabutylammonium hydrogen sulfate, NaOH, DCM, RT, 16h (iii) hydroxylamine hydrochloride, $NaHCO_3$, ^iPrOH, RT to reflux, 16h (iv) TFAA, pyridine, 80 °C, 16h

Step i: Data for 5-(chloromethyl)-N,N-dimethyl-isoxazole-3-carboxamide

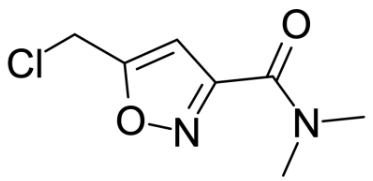

Oxalyl chloride (169 mg, 1.33 mmol, 116 μL) and a drop of DMF were added to a stirred solution of 5-(chloromethyl)isoxazole-3-carboxylic acid (143 mg, 885 μmol) in DCM (10 mL) at room temperature. The resulting mixture was stirred at room temperature for 1 hr. The reaction mixture was concentrated under reduced pressure. The residue was redissolved in THF (6.0 mL) and added dropwise to a solution of dimethylamine (79.4 mg, 974 μmol, 122 μL, HCl) and DIPEA (137 mg, 1.06 mmol, 185 μL) in THF (6.0 mL) at 0°C. The resulting

mixture was allowed to warm up to room temperature and stirred overnight. The reaction mixture was concentrated under reduced pressure. The residue was partitioned between EtOAc (10 mL) and water (5.0 mL). The aqueous layer was extracted with EtOAc (5.0 mL). The combined organic layers were washed with brine (5.0 mL), dried over anhydrous sodium sulfate and concentrated under reduced pressure to afford 5-(chloromethyl)-N,N-dimethyl-isoxazole-3-carboxamide (150 mg, 740 μmol, 83.6% yield) as red oil which was used in the next step without further purification. LCMS(ESI): $[M+H]^+$ m/z: calcd 189.05; found 189.0.

Step ii: Data for 5-[2-(4-cyano-2-methyl-phenyl)ethoxymethyl]-N,N-dimethyl-isoxazole-3-carboxamide

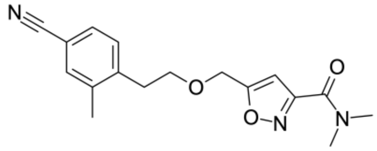

Tetrabutylammonium hydrogen sulfate (31.6 mg, 93.1 μmol) and 5-(chloromethyl)-N,N-dimethyl-isoxazole-3-carboxamide (105 mg, 558 μmol) were added to a vigorously stirred mixture of a solution of 4-(2-hydroxyethyl)-3-methyl-benzonitrile (7.50 mg, 465 μmol) in DCM (10 mL) and aqueous NaOH solution (5.0 mL, 15% wt.) at room temperature. The resulting mixture was stirred at room temperature overnight. The reaction mixture was washed with water (10 mL). The aqueous layer was separated and extracted with DCM (2×10 mL). The combined organic layers were washed with water (10 mL), brine (10 mL), dried over anhydrous sodium sulfate and concentrated under reduced pressure to afford 5-[2-(4-cyano-2-methyl-phenyl)ethoxymethyl]-N,N-dimethyl-isoxazole-3-carboxamide (150 mg, 192 μmol, 41.2% yield) as a brown oil which was used in the next step without further purification. LCMS(ESI): $[M+H]^+$ m/z: calcd 314.15; found 314.2.

Step iii: Data for 5-[2-[4-(N-hydroxycarbamimidoyl)-2-methyl-phenyl] ethoxymethyl]-N,N-dimethyl-isoxazole-3-carboxamide

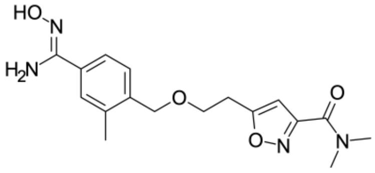

$NaHCO_3$ (96.5 mg, 1.15 mmol) and hydroxylamine hydrochloride (39.9 mg, 574 μmol) were added to a stirred solution of 5-[2-(4-cyano-2-methyl-phenyl)ethoxymethyl]-N,N-dimethyl-isoxazole-3-carboxamide (150 mg, 192 μmol) in iPrOH (10.1 mL) at room temperature. The resulting mixture was refluxed overnight. The reaction mixture was cooled to room temperature and filtered. The filter cake was washed with iPrOH (5 mL). The filtrate was concentrated under reduced pressure to afford 5-[2-[4-(N-hydroxycarbamimidoyl)-2-methyl-phenyl]ethoxymethyl]-N,N-dimethyl-isoxazole-3-carboxamide (130 mg, 184 μmol, 96.1% yield) as a brown solid which was used in the next step without further purification. LCMS(ESI): $[M+H]^+$ m/z: calcd 347.17; found 347.2.

Step iv: Synthesis of N,N-dimethyl-5-[2-[2-methyl-4-[5-(trifluoromethyl)-1,2,4-oxadiazol-3-yl]phenyl]ethoxymethyl]isoxazole-3-carboxamide (Compound 23)

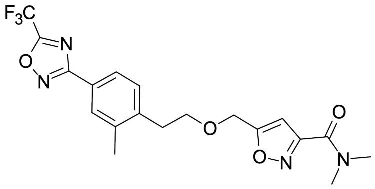

TFAA (116 mg, 552 μmol, 76.7 μL) was added to a stirred solution of 5-[2-[4-(N-hydroxycarbamimidoyl)-2-methyl-phenyl]ethoxymethyl]-N,N-dimethyl-isoxazole-3- carboxamide (130 mg, 184 μmol) in pyridine (4.9 mL). The resulting mixture was stirred at 80 °C overnight. The reaction mixture was cooled to room temperature and concentrated under reduced pressure. The residue was subjected to reverse phase HPLC (0-1-5 min., 45-45-75% water – ACN, flow: 30 mL/min, column: Chromatorex 18 SMB100-5T 100×19 mm, 5 μm) to afford N,N-dimethyl-5-[2-[2-methyl-4-[5-(trifluoromethyl)-1,2,4-oxadiazol-3-

yl]phenyl]ethoxymethyl]isoxazole-3-carboxamide (58.0 mg, 137 μmol, 74.3% yield) as a brown solid. ^{1}H NMR (500 MHz, dmso) δ_H 2.37 (s, 3H), 2.94 (t, 2H), 2.98 (s, 3H), 3.03 (s, 3H), 3.74 (t, 2H), 4.67 (s, 2H), 6.62 (s, 1H), 7.41 (d, 1H), 7.80 (d, 1H), 7.84 (s, 1H). LCMS(ESI): $[M+H]^+$ m/z: calcd 425.16; found 425.0.

Synthesis of N,N-dimethyl-5-[2-[[2-methyl-4-[5-(trifluoromethyl) - 1,2,4-oxadiazol-3-yl]phenyl]methoxy]ethyl]isoxazole-3-carboxamide (Compound 24)

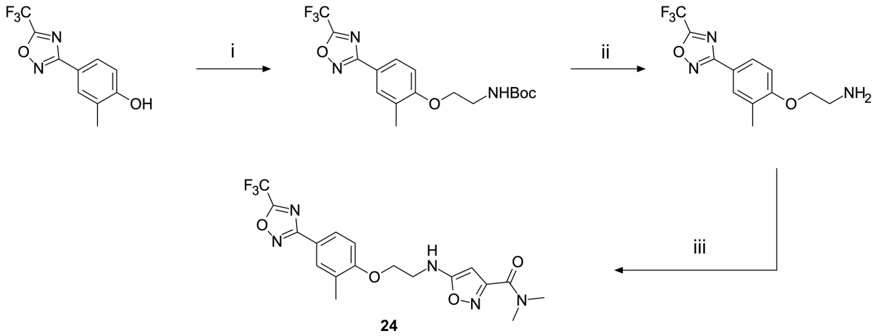

Scheme S4: Reagents and conditions: (i) tert-butyl 2,2-dioxooxathiazolidine-3-carboxylate, NaH, DMF, RT, 15h (ii) AcCl, MeOH, 0 °C to RT, 16h (iii) 5-chloro-N,N-dimethyl-isoxazole-3-carboxamide, K_2CO_3, DMF, 120 °C 48h

Step i: Data for tert-butyl N-[2-[2-methyl-4-[5-(trifluoromethyl)-1,2,4-oxadiazol-3-yl]phenoxy]ethyl]carbamate

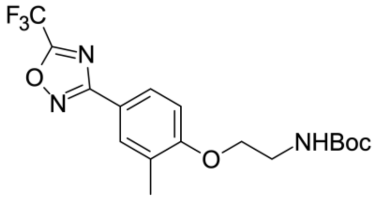

NaH (23.6 mg, 983 μmol, 60% dispersion in mineral oil) was added to a solution of 2-methyl-4-[5-(trifluoromethyl)-1,2,4-oxadiazol-3-yl]phenol (200 mg, 819 μmol) in DMF (5.0 mL) at room temperature. The resulting mixture was stirred at ambient temperature for 2 hr. Then, tert-butyl 2,2-dioxooxathiazolidine-3-carboxylate (219 mg, 983 μmol) was added in one portion to the mixture. The resulting mixture was stirred at room temperature for 15 hr. The reaction

mixture was concentrated under reduced pressure. The residue was redissolved in EtOAc (30 mL) and washed with brine (3×30 mL). The combined organic layers were dried over anhydrous sodium sulfate and concentrated under reduced pressure to afford tert-butyl N-[2-[2-methyl-4-[5-(trifluoromethyl)-1,2,4-oxadiazol-3-yl]phenoxy]ethyl]carbamate (100 mg, 258 μmol, 31.5% yield) as a yellow solid which was used in the next step without further purification. ^{1}H NMR (500 MHz, CDCl3) δ_H 1.46 (s, 9H), 2.29 (s, 3H), 3.54 - 3.65 (m, 2H), 4.10 (t, 2H), 4.94 (br. s, 1H), 6.90 (d, 1H), 7.88 – 7.95 (m, 2H). LCMS(ESI): [M+H-Boc]+ m/z: calcd 288.1; found 288.0.

Step ii: Data for 2-[2-methyl-4-[5-(trifluoromethyl)-1,2,4-oxadiazol-3-yl]phenoxy]ethanamine

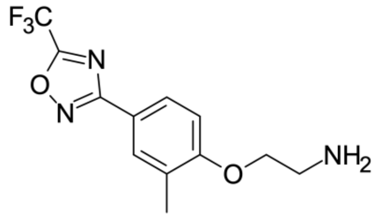

Acetyl chloride (30.4 mg, 387 μmol, 23.5 μL) was added dropwise to a stirred MeOH (5.0 mL) at 0 ºC. Tert-butyl N-[2-[2-methyl-4-[5-(trifluoromethyl)-1,2,4-oxadiazol-3-yl]phenoxy]ethyl]carbamate (100 mg, 258 μmol) was added to the obtained solution. The resulting mixture was stirred at room temperature for 15 hr. The reaction mixture was concentrated under reduced pressure to afford 2-[2-methyl-4-[5-(trifluoromethyl)-1,2,4-oxadiazol-3-yl]phenoxy]ethanamine (75.0 mg, 232 μmol, 89.8% yield, HCl) as a yellow solid which was used in the next step without further purification. LCMS(ESI): $[M+H]^+$ m/z: calcd 288.1; found 288.2.

Step iii: Data for N,N-dimethyl-5-[2-[2-methyl-4-[5-(trifluoromethyl)-1,2,4-oxadiazol-3-yl]phenoxy]ethylamino]isoxazole-3-carboxamide (Compound 24)

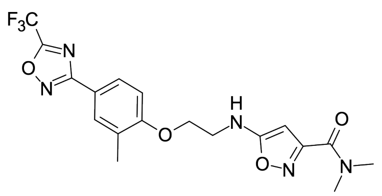

Potassium carbonate (96.1 mg, 695 μmol) and 5-chloro-N,N-dimethyl-isoxazole-3-carboxamide (40.5 mg, 232 μmol) were added to a solution of 2-[2-methyl-4-[5-(trifluoromethyl)-1,2,4-oxadiazol-3-yl]phenoxy]ethanamine (75.0 mg, 232 μmol, HCl) in DMF (4.0 mL). The resulting mixture was stirred at 120 °C for 48 hr. The reaction mixture was cooled to room temperature, filtered and subjected to HPLC (0-1-5 min., 30-30-60% water – ACN, flow: 30 mL/min, column: Chromatorex 18 SMB100-5T 100×19 mm, 5 μm) to afford N,N-dimethyl-5-[2-[2-methyl-4-[5-(trifluoromethyl)-1,2,4-oxadiazol-3-yl]phenoxy]ethylamino]isoxazole-3-carboxamide (1.50 mg, 3.53 μmol, 1.52% yield) as a yellow gum. ^{1}H NMR (500 MHz, CD_3OD) δ_H 2.29 (s, 3H), 3.07 (s, 3H), 3.21 (s, 3H), 3.86 (t, 2H), 4.12 (t, 2H), 5.47 (s, 1H), 7.25 (d, 1H), 7.60 (dd, 1H), 7.69 (d, 1H). LCMS(ESI): $[M+H]^+$ m/z: calcd 426.15; found 426.2.

Synthesis of 5-[3-(5-cyanoindol-1-yl)propyl]-N,N-dimethyl-isoxazole-3-carboxamide (Compound 25) and N,N-dimethyl-5-[3-[5-[5-(trifluoromethyl)-1,2,4-oxadiazol-3-yl]indol-1-yl]propyl]isoxazole-3-carboxamide (Compound 26)

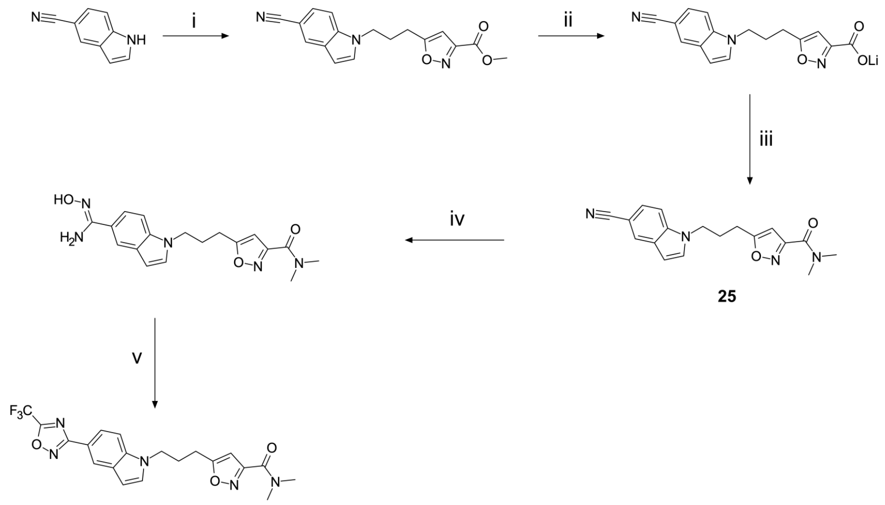

Scheme S5: Reagents and conditions: (i) methyl 5-(3-bromopropyl)isoxazole-3-carboxylate, NaH, DMF, 0 °C to 50 °C, 16h (ii) LiOH, H_2O, 0 °C to RT, 16h (iii) Me_2NH, HATU, DIPEA, DMF, RT, 16h (iv) hydroxylamine hydrochloride, $NaHCO_3$, EtOH, RT to 70 °C, 16h (v) TFAA, RT, 16h

Step i: Data for methyl 5-[3-(5-cyanopyrrolo[3,2-b]pyridin-1-yl)propyl] isoxazole-3-carboxylate

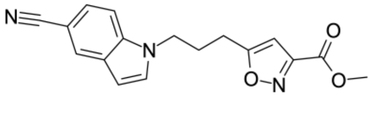

1H-indole-5-carbonitrile (249 mg, 1.39 mmol, HCl) was added to a stirred mixture of NaH (139 mg, 3.48 mmol, 60% dispersion in mineral oil) in DMF (20 mL) at 0 °C. After stirring for 30 min, methyl 5-(3-bromopropyl)isoxazole-3-carboxylate (449 mg, 1.81 mmol) was added

at 0 °C to the mixture. The resulting mixture was stirred at 50 °C for 16 hr. The reaction mixture was cooled to room temperature and diluted with water (30 mL). The resulting mixture was extracted with EtOAC (3×15 mL). The combined organic layers were washed with water (3×15 mL) and brine (15 mL), dried over anhydrous sodium sulfate and concentrated under reduced pressure. The residue was subjected to column chromatography (SiO_2, Hexane-MTBE 7:3) to afford methyl 5-[3-(5-cyanopyrrolo[3,2-b]pyridin-1-yl)propyl]isoxazole-3-carboxylate (600 mg, 1.55 mmol, 92.4% yield) as a yellow solid. ^{1}H NMR (500 MHz, dmso) δ_H 2.18 (p, 2H), 2.81 (t, 2H), 3.87 (s, 3H), 4.33 (t, 2H), 6.61 (d, 1H), 6.68 (s, 1H), 7.49 (d, 1H), 7.62 (d, 1H), 7.69 (d, 1H), 8.08 (s, 1H). LCMS(ESI): $[M+H]^+$ m/z: calcd 310.12; found 310.2.

Step ii: Data for [5-[3-(5-cyanopyrrolo[3,2-b]pyridin-1-yl)propyl]isoxazole-3-carbonyl]oxylithium

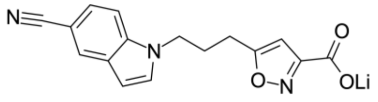

Water (200 μL) and LiOH·H_2O (200 mg, 645 μmol) were added to a stirred solution of methyl 5-[3-(5-cyanopyrrolo[3,2-b]pyridin-1-yl)propyl]isoxazole-3-carboxylate (200 mg, 645 μmol) in THF (5.0 mL) at 0 °C. The resulting mixture was stirred at room temperature for 16 hr. The reaction mixture was concentrated under reduced pressure to afford [5-[3-(5-cyanopyrrolo[3,2-b]pyridin-1-yl)propyl]isoxazole-3-carbonyl]oxylithium (340 mg, 824 μmol, 72.8% yield) as a yellow gum which was used in the next step without further purification. LCMS(ESI): [M-Li]- m/z: calcd 294.3; found 294.0.

Step iii: Data for 5-[3-(5-cyanoindol-1-yl)propyl]-N,N-dimethyl-isoxazole-3-carboxamide

(Compound 25)

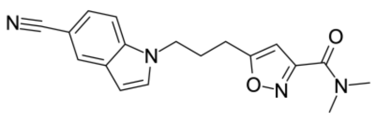

N-Methylmethanamine (54.0 mg, 662 μmol, 83.0 μL, HCl), HATU (302 mg, 794 μmol) and DIPEA (257 mg, 1.99 mmol, 346 μL) were added sequentially to a stirred solution of [5-[3-(5-cyanoindol-1-yl)propyl]isoxazole-3-carbonyl]oxylithium (199 mg, 662 μmol) in DMF (10 mL) at room temperature. The resulting mixture was stirred at room temperature for 16 hr. The reaction mixture was diluted with water (20 mL). The resulting mixture was extracted with EtOAc (2×15 mL). The combined organic layers were washed with an aqueous solution of NH_4Cl (10 mL), an aqueous solution of $NaHCO_3$ (10 mL), water (2×10 mL) and brine (10 mL). The organic layer was dried over anhydrous sodium sulfate and concentrated under reduced pressure to afford 5-[3-(5-cyanoindol-1-yl)propyl]-N,N-dimethyl-isoxazole-3-carboxamide (180 mg, 452 μmol, 80.1% yield) as a yellow solid which was used in the next step without further purification. LCMS(ESI): $[M+H]^+$ m/z: calcd 323.15; found 323.2.

Note: purification of a similar sample under HPLC conditions (0-1-5 min., 40-40-65% water – MeOH, flow: 30 mL/min, column: Chromatorex 18 SMB100-5T 100×19 mm, 5 μm) provided a pure sample with the following NMR data: ^{1}H NMR (500 MHz, CD_3OD) δ_H 2.28 (p, 2H), 2.81 (t, 2H), 3.09 (s, 3H), 3.16 (s, 3H), 4.34 (t, 2H), 6.31 (s, 1H), 6.61 (d, 1H), 7.41 – 7.46 (m, 2H), 7.57 (d, 1H), 7.98 (s, 1H).

Step iv: Data for 5-[3-[5-[(Z)-N'-hydroxycarbamimidoyl]indol-1-yl]propyl]-N,N-dimethyl-isoxazole-3-carboxamide

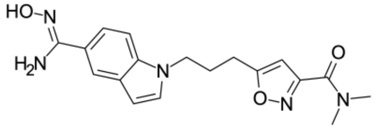

Hydroxylamine hydrochloride (65.8 mg, 946 μmol) and $NaHCO_3$ (88.3 mg, 1.05 mmol) were added respectively at room temperature to a stirred solution of 5-[3-(5-cyanoindol-1-yl)propyl]-N,N-dimethyl-isoxazole-3-carboxamide (167 mg, 526 μmol) in EtOH (15 mL). The resulting mixture was stirred at 70 °C for 16 hr. The reaction mixture was cooled to room temperature and filtered. The filtrate was concentrated under reduced pressure to afford 5-[3-[5-[(Z)-N'-hydroxycarbamimidoyl]indol-1-yl]propyl]-N,N-

dimethyl-isoxazole-3-carboxamide (200 mg, 310 μmol, 55.4% yield) as a yellow gum which was used in the next step without further purification. LCMS(ESI): $[M+H]^+$ m/z: calcd 356.17; found 356.2.

Step iii: Data for N,N-dimethyl-5-[3-[5-[5-(trifluoromethyl)-1,2,4-oxadiazol-3-yl]indol-1-yl]propyl]isoxazole-3-carboxamide (Compound 26)

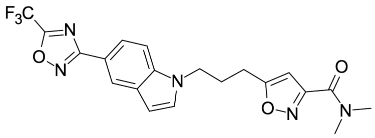

TFAA (150 mg, 716 μmol, 99.5 μL) was added to a stirred solution of 5-[3-[5-[(Z)-N'-hydroxycarbamimidoyl]indol-1-yl]propyl]-N,N-dimethyl-isoxazole-3-carboxamide (170 mg, 477 μmol) in THF (10 mL) at room temperature. The resulting mixture was stirred at room temperature for 16 hr. The reaction mixture was concentrated under reduced pressure. The residue was quenched with an aqueous solution of $NaHCO_3$ (10 mL). The aqueous layer was separated and extracted with MTBE (2×10 mL). The combined organic layers were washed with water (10 mL), brine (10 mL), dried over anhydrous sodium sulfate and concentrated under reduced pressure. The residue was subjected to reverse phase HPLC (0-1-5 min., 55-55-75% water – ACN, flow: 30 mL/min, column: Chromatorex 18 SMB100-5T 100×19 mm, 5 μm) to afford N,N-dimethyl-5-[3-[5-[5-(trifluoromethyl)-1,2,4-oxadiazol-3-yl]indol-1-yl]propyl]isoxazole-3-carboxamide (68.0 mg, 157 μmol, 51.6% yield) as an off-white gum. ^{1}H NMR (500 MHz, dmso) δ_H 2.19 (p, 2H), 2.79 (t, 2H), 2.96 (s, 3H), 3.01 (s, 3H), 4.33 (t, 2H), 6.47 (s, 1H), 6.65 (d, 1H), 7.56 (d, 1H), 7.71 (d, 1H), 7.81 (d, 1H), 8.31 (s, 1H). LCMS(ESI): $[M+H]^+$ m/z: calcd 434.16; found 434.2.

Synthesis of N,N-dimethyl-5-[3-[5-[5-(trifluoromethyl)-1,2,4-oxadiazol-3-yl]indazol-1-yl]propyl]isoxazole-3-carboxamide (Compound 27)

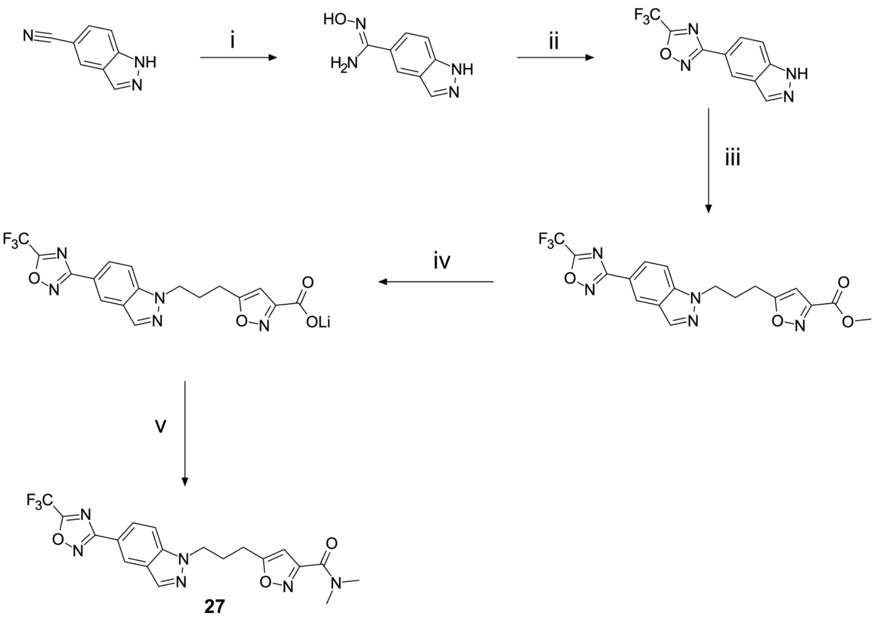

27

Scheme S6: Reagents and conditions: (i) hydroxylamine hydrochloride, $NaHCO_3$, MeOH, RT, 16h (ii) TFAA, THF, RT, 16h (iii) methyl 5-(3-bromopropyl)isoxazole-3-carboxylate, K_2CO_3, DMF, RT to 50 °C, 16h (iv) LiOH, H_2O, MeOH, RT, 16h (v) Me_2NH, HATU, DIPEA, DMF, RT to 50 °C, 16h

Step i: Data for N'-hydroxy-1H-indazole-5-carboxamidine

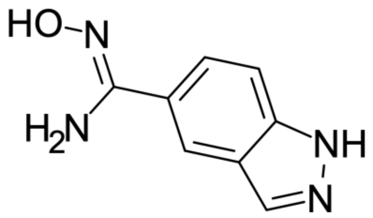

$NaHCO_3$ (1.47 g, 17.5 mmol) and hydroxylamine hydrochloride (485 mg, 6.99 mmol) were added to a stirred solution of 1H-indazole-5-carbonitrile (500 mg, 3.49 mmol) in MeOH (18 mL) at room temperature. The resulting mixture was stirred at room temperature overnight. The reaction mixture was filtered. The filtercake was washed with MeOH (2×5 mL). The combined filtrate was concentrated under reduced pressure to afford N'-hydroxy-1H-indazole-5-carboxamidine (600 mg, 2.72 mmol, 78.0% yield) as a brown solid which was used in the next step without further purification. LCMS(ESI): $[M+H]^+$ m/z: calcd 177.07; found 177.2.

Step ii: Data for 3-(1H-indazol-5-yl)-5-(trifluoromethyl)-1,2,4-oxadiazole

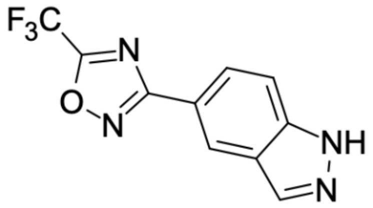

TFAA (1.45 g, 6.90 mmol, 960 μL) was added to a stirred solution of N'-hydroxy-1H-indazole-5-carboxamidine (600 mg, 2.72 mmol) in THF (15.0 mL) at room temperature. The resulting mixture was stirred at room temperature overnight. The reaction mixture was concentrated under reduced pressure. The residue was partitioned between EtOAc (20 mL) and water (10 mL). The aqueous layer was separated and extracted with EtOAc (10 mL). The combined organic layers were washed with water (10 mL) and brine (10 mL), dried over anhydrous sodium sulfate and concentrated under reduced pressure to afford 3-(1H-indazol-5-yl)-5-(trifluoromethyl)-1,2,4-oxadiazole (700 mg, 1.79 mmol, 65.7% yield) as a brown solid which was used in the next step without further purification. ^{1}H NMR (500 MHz, dmso) δ_H 7.76 (d, 1H), 8.00 (d, 1H), 8.28 (s, 1H), 8.57 (s, 1H), 13.37 (br. s, 1H). LCMS(ESI): $[M+H]^+$ m/z: calcd 255.05; found 255.0.

Step iii: Data for methyl 5-[3-[5-[5-(trifluoromethyl)-1,2,4-oxadiazol-3-yl]indazol-1-yl]propyl]isoxazole-3-carboxylate

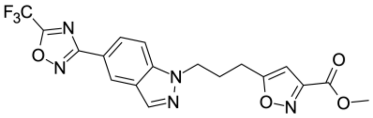

Methyl 5-(3-bromopropyl)isoxazole-3-carboxylate (533 mg, 2.15 mmol) and K_2CO_3 (619 mg, 4.48 mmol) were added to a stirred solution of 3-(1H-indazol-5-yl)-5-(trifluoromethyl)-1,2,4-oxadiazole (700 mg, 1.79 mmol) in DMF (7.0 mL) at room temperature. The resulting mixture was stirred at 50 °C overnight. The reaction mixture was cooled to room temperature and filtered. The filtrate was subjected to preparative HPLC (0-1-6 min., 50-50-70% water – ACN, flow: 30 mL/min, column: Chromatorex 18 SMB100-5T 100×19 mm, 5 μm) to afford methyl 5-[3-[5-[5-(trifluoromethyl)-1,2,4-oxadiazol-3-yl]indazol-1-yl]propyl]isoxazole-3-carboxylate (89.0 mg, 211 μmol, 11.8% yield) as a yellow

solid. ^{1}H NMR (500 MHz, CD_3OD) δ_H 2.41 (p, 2H), 2.92 (t, 2H), 3.95 (s, 3H), 4.62 (t, 2H), 6.58 (s, 1H), 7.85 (d, 1H), 8.14 (d, 1H), 8.29 (s, 1H), 8.62 (s, 1H). LCMS(ESI): $[M+H]^+$ m/z: calcd 422.11; found 422.2.

Step iv: Data for [5-[3-[5-[5-(trifluoromethyl)-1,2,4-oxadiazol-3-yl]indazol-1-yl]propyl]isoxazole-3-carbonyl]oxylithium

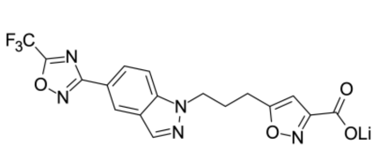

$LiOH \cdot H_2O$ (10.8 mg, 256 μmol) in water (1.0 mL) was added to a stirred solution of methyl 5-[3-[5-[5-(trifluoromethyl)-1,2,4-oxadiazol-3-yl]indazol-1-yl]propyl]isoxazole-3-carboxylate (90.0 mg, 214 μmol) in THF (1.0 mL) and MeOH (1.0 mL) at room temperature. The resulting mixture was stirred at room temperature overnight. The reaction mixture was concentrated under reduced pressure to afford [5-[3-[5-[5-(trifluoromethyl)-1,2,4-oxadiazol-3-yl]indazol-1-yl]propyl]isoxazole-3-carbonyl]oxylithium (100 mg, 26.6 μmol, 12.5% yield) as a brown solid which was used in the next step without further purification. LCMS(ESI): [M-Li+2H]+ m/z: calcd 408.09; found 408.0.

Step v: Data for N,N-dimethyl-5-[3-[5-[5-(trifluoromethyl)-1,2,4-oxadiazol-3-yl]indazol-1-yl]propyl]isoxazole-3-carboxamide (Compound 27)

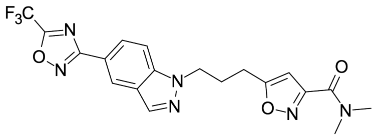

DIPEA (103 mg, 799 μmol, 139 μL) and HATU (202 mg, 532 μmol) were added to a stirred solution of [5-[3-[5-[5-(trifluoromethyl)-1,2,4-oxadiazol-3-yl]indazol-1-yl]propyl]isoxazole-3-carbonyl]oxylithium (100 mg, 26.6 μmol) and dimethylamine (32.6 mg, 399 μmol, 50.1 μL, HCl) in DMF (3.0 mL) at room temperature. The resulting mixture was stirred at 50 °C overnight. The reaction mix-

ture was cooled to room temperature and subjected to preparative HPLC (0-5 min., 60-85% water – MeOH, flow: 30 mL/min, column: Chromatorex 18 SMB100-5T 100×19 mm, 5 μm) to afford N,N-dimethyl-5-[3-[5-[5-(trifluoromethyl)-1,2,4-oxadiazol-3-yl]indazol-1-yl]propyl]isoxazole-3-carboxamide (7.50 mg, 17.3 μmol, 64.9% yield) as a beige solid. ^{1}H NMR (500 MHz, dmso) δ_H 2.26 (p, 2H), 2.81 (t, 2H), 2.96 (s, 3H), 3.01 (s, 3H), 4.55 (t, 2H), 6.45 (s, 1H), 7.90 (d, 1H), 8.03 (d, 1H), 8.29 (s, 1H), 8.55 (s, 1H). LCMS(ESI): $[M+H]^+$ m/z: calcd 435.15; found 435.0.

Data for N,N-dimethyl-5-[3-[5-[5-(trifluoromethyl)-1,2,4-oxadiazol-3-yl]pyrrolo[3,2-b]pyridin-1-yl]propyl]isoxazole-3-carboxamide (Compound 28)

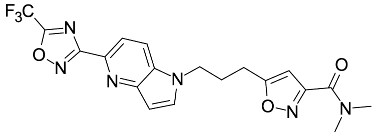

Compound 28 was made by a similar route to compound 26, starting with 1H-Pyrrolo[3,2-b]pyridine-5-carbonitrile, affording N,N-dimethyl-5-[3-[5-[5-(trifluoromethyl)-1,2,4-oxa diazol-3-yl]pyrrolo[3,2-b]pyridin-1-yl]propyl]isoxazole-3-carbox amide (56.0 mg, 129 μmol, 27.0% yield) as an off-white gum. ^{1}H NMR (500 MHz, dmso) δ_H 2.21 (p, 2H), 2.79 (t, 2H), 2.96 (s, 3H), 3.01 (s, 3H), 4.36 (t, 2H), 6.46 (s, 1H), 6.75 (d, 1H), 7.90 – 7.96 (m, 2H), 8.18 (d, 1H). LCMS(ESI): $[M+H]^+$ m/z: calcd 435.15; found 435.2.

Data for N,N-dimethyl-5-[3-[5-[5-(trifluoromethyl)-1,2,4-oxadiazol-3-yl]pyrrolo[2,3-b]pyridin-1-yl]propyl]isoxazole-3-carboxamide (Compound 29)

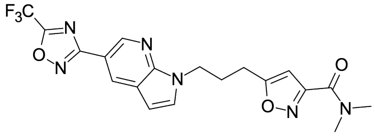

Compound 29 was made by a similar route to compound 26, starting with 1H-pyrrolo[2,3-b]pyridine-5-carbonitrile, affording N,N-dimethyl-5-[3-[5-[5-(trifluoromethyl)-1,2,4-oxadiazol-3-yl]pyrrolo[2,3-b]pyridin-1-yl]propyl]isoxazole-3-carboxamide

(150 mg, 345 μmol, 35.2% yield) as an off-white solid. ^{1}H NMR (500 MHz, dmso) δ_H 2.24 (p, 2H), 2.80 (t, 2H), 2.97 (s, 3H), 3.02 (s, 3H), 4.40 (t, 2H), 6.47 (s, 1H), 6.68 (d, 1H), 7.78 (d, 1H), 8.64 (d, 1H), 8.90 (d, 1H). LCMS(ESI): $[M+H]^+$ m/z: calcd 435.15; found 435.2.

Data for N,N-dimethyl-5-[3-[5-[5-(trifluoromethyl)-1,2,4-oxadiazol-3-yl]pyrrolo[2,3-c]pyridin-1-yl]propyl]isoxazole-3-carboxamide (Compound 30)

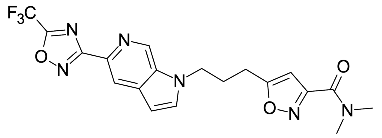

Compound 30 was made by a similar route to compound 26, starting with 1H-pyrrolo[2,3-c]pyridine-5-carbonitrile, affording N,N-dimethyl-5-[3-[5-[5-(trifluoromethyl)-1,2,4-oxadiazol-3-yl]pyrrolo[2,3-c]pyridin-1- yl]propyl]isoxazole-3-carboxamide (174 mg, 401 μmol, 35.7% yield) as a white solid. ^{1}H NMR (500 MHz, dmso) δ_H 2.25 (p, 2H), 2.81 (t, 2H), 2.96 (s, 3H), 3.01 (s, 3H), 4.47 (t, 2H), 6.48 (s, 1H), 6.73 (d, 1H), 7.80 (d, 1H), 8.39 (s, 1H), 9.08 (s, 1H). LCMS(ESI): $[M+H]^+$ m/z: calcd 435.15; found 435.2.

Synthesis of N,N-dimethyl-5-[3-[4-[5-(trifluoromethyl)-1,2,4-oxadiazol-3-yl]phenoxy]phenyl]isoxazole-3-carboxamide (Compound 31)

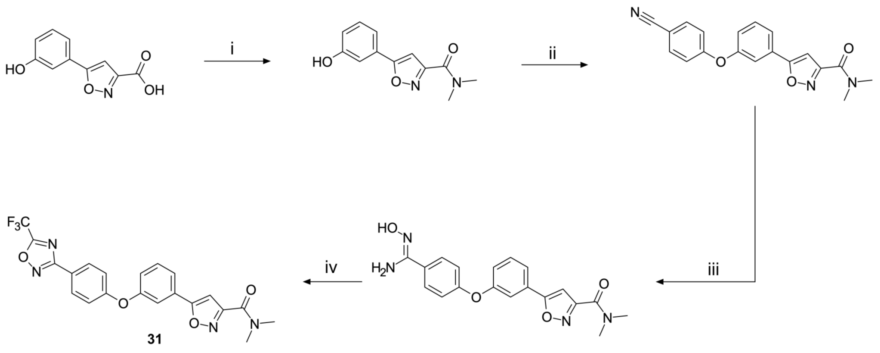

Scheme S7: Reagents and conditions: (i) Me_2NH, HATU, DIPEA, DMF, 50 °C, 16h (ii) 4-fluorobenzonitrile, K_2CO_3, 100 °C, 16h (iii) hydroxylamine hydrochloride, $NaHCO_3$, $^{i}PrOH$, 75 °C, 16h (iv) TFAA, pyridine, 50 °C, 16h

Step i: Data for 5-(3-hydroxyphenyl)-N,N-dimethyl-isoxazole-3-carboxamide

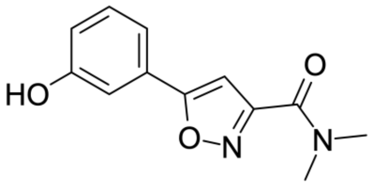

5-(3-Hydroxyphenyl)isoxazole-3-carboxylic acid (1.00 g, 4.87 mmol), HATU (2.22 g, 5.85 mmol) and N-methylmethanamine (1.99 g, 24.4 mmol, 3.06 mL) and DIPEA (6.30 g, 48.7 mmol) were mixed in dry DMF (30 mL). The resulting mixture was stirred at 50 °C overnight. The reaction mixture was cooled to room temperature, diluted with water (200 mL) and extracted with EtOAc (3×50 mL). The combined organic layers were washed with water (3×100 mL) and brine (100 mL), dried over anhydrous sodium sulfate and filtered. The filtrate was concentrated under reduced pressure to afford 5-(3-hydroxyphenyl)-N,N-dimethyl-isoxazole-3-carboxamide (620 mg, crude) as a yellow solid which was used in the next step without further purification. LCMS(ESI): $[M+H]^+$ m/z: calcd 233.09; found 233.0.

Step ii: Data for 5-[3-(4-cyanophenoxy)phenyl]-N,N-dimethyl-isoxazole-3-carboxamide

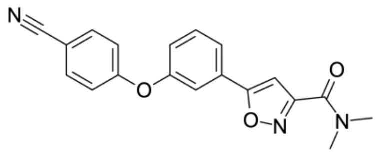

5-(3-Hydroxyphenyl)-N,N-dimethyl-isoxazole-3-carboxamide (620 mg, 2.67 mmol), K_2CO_3 (738 mg, 5.34 mmol) and 4-fluorobenzonitrile (388 mg, 3.20 mmol) were mixed in dry DMF (5 mL). The resulting mixture was stirred at 100 °C overnight. The mixture was cooled to room temperature and diluted with water (10 mL). The formed precipitate was collected by filtration, washed with water (5 mL) and air dried to afford 5-[3-(4-cyanophenoxy)phenyl]-N,N-dimethyl-isoxazole-3-carboxamide (288 mg, 734 μmol, 27.5% yield) as a brown solid which was used in the next step without further purification. ^{1}H NMR (500 MHz, dmso) δ_H 3.03 (s, 3H), 3.11 (s, 3H), 7.19 (d, 2H), 7.32 (d, 1H), 7.36 (s, 1H), 7.65 (t, 1H), 7.73 (s, 1H), 7.83 (d, 1H), 7.88 (d, 2H). LCMS(ESI): $[M+H]^+$ m/z: calcd 334.12; found 334.0.

Step iii: Data for 5-[3-[4-[(Z)-N'-hydroxycarbamimidoyl]phenoxy]phenyl]-N,N-dimethyl-isoxazole-3-carboxamide

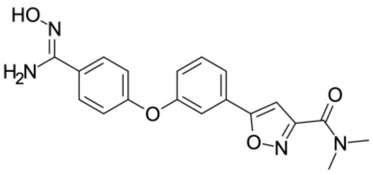

Hydroxylamine hydrochloride (62.5 mg, 900 μmol) and $NaHCO_3$ (151 mg, 1.80 mmol) were added to a solution of 5-[3-(4-cyanophenoxy)phenyl]-N,N-dimethyl-isoxazole-3-carboxamide (100 mg, 300 μmol) in i-PrOH (3.0 mL). The resulting mixture was stirred at 75 °C overnight. The reaction mixture was cooled to room temperature and filtered. The filtrate was concentrated under reduced pressure to afford 5-[3-[4-[(Z)-N'-hydroxycarbamimidoyl]phenoxy]phenyl]-N,N-dimethyl-isoxazole-3-carboxamide (80.0 mg, crude) as a yellow solid which was used in the next step without further purification. LCMS(ESI): $[M+H]^+$ m/z: calcd 367.14; found 367.0.

Step iv: Data for N,N-dimethyl-5-[3-[4-[5-(trifluoromethyl)-1,2,4-oxadiazol-3-yl]phenoxy]phenyl]isoxazole-3-carboxamide (Compound 31)

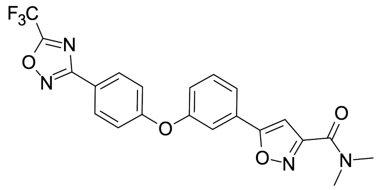

Trifluoroacetic anhydride (138 mg, 655 μmol, 91.0 μL) was added to a solution of 5-[3-[4-[(Z)-N'-hydroxycarbamimidoyl] phenoxy]phenyl]-N,N-dimethyl-isoxazole-3-carboxamide (80.0 mg, 218 μmol) in pyridine (1.0 mL). The resulting mixture was stirred at 50 °C overnight. The reaction mixture was cooled to room temperature and concentrated under reduced pressure. The residue was re-disolved with ACN (2.0 mL) and subjected to HPLC (0-1-6 min., 40-40-90% water – ACN, flow: 60 mL/min, column: XBridge OBD 100×30 mm, 5 μm) to afford N,N-dimethyl-5-[3-[4-[5-(trifluoromethyl)-1,2,4-oxadiazol-3-yl]phenoxy]phenyl]isoxazole-3-carboxamide (35.3 mg, 78.5 μmol, 35.9% yield) as a yellow oil. ^{1}H NMR (500 MHz, dmso) δ_H 3.03 (s, 3H), 3.11 (s, 3H), 7.26 (d, 2H), 7.33 (d, 1H), 7.36 (s, 1H), 7.66 (t, 1H), 7.73 (s, 1H), 7.82 (d, 1H), 8.10 (d, 2H). LCMS(ESI): $[M+H]^+$ m/z: calcd 445.12; found 445.2.

Synthesis of N,N-dimethyl-5-[5-[2-methyl-4-[5-(trifluoromethyl)-1,2,4-oxadiazol-3-yl]phenoxy]-3-pyridyl]isoxazole-3-carboxamide (Compound 32)

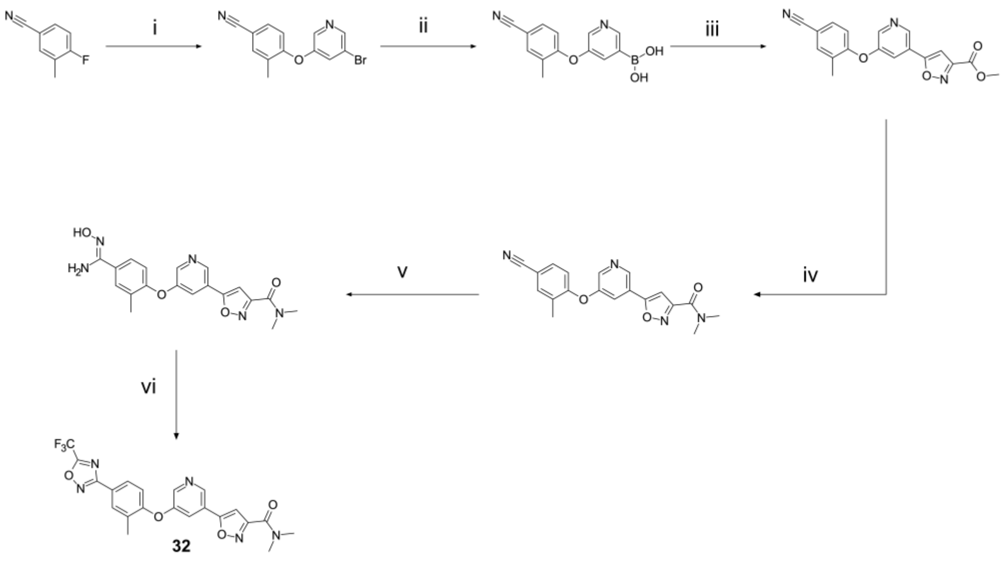

Scheme S8: Reagents and conditions: (i) 5-bromopyridin-3-ol, K_2CO_3, DMF, 115 °C, 15h (ii) bis(pinacolato)diboron, Pd(dppf)Cl_2, KOAc, dioxane, 90 °C, 15h (iii) methyl 5-bromoisoxazole-3-carboxylate, Na_2CO_3, Pd(dppf)Cl_2, H_2O, dioxane, 90 °C, 15h (iv) dimethylamine, H_2O, THF, 60 °C, 16h (v) hydroxylamine hydrochloride, EtOH, 70 °C, 15h (vi) TFAA, THF, RT, 15h

Step i: Data for 4-[(5-bromo-3-pyridyl)oxy]-3-methyl-benzonitrile

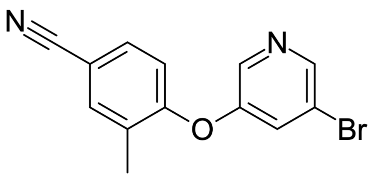

Potassium carbonate (1.59 g, 11.5 mmol) and 4-fluoro-3-methyl-benzonitrile (777 mg, 5.75 mmol) were added in one portion to a solution of 5-bromopyridin-3-ol (1.00 g, 5.75 mmol) in DMF (20 mL). The resulting mixture was stirred at 115 °C for 15 hr. The reaction mixture was cooled to room temperature, filtered and concentrated under reduced pressure. The residue was diluted with EtOAc (40 mL) the resulting mixture was washed with brine (3×20 mL). The obtained organic layer was dried over anhydrous sodium sulfate and concentrated under reduced pres-

sure to afford 4-[(5-bromo-3-pyridyl)oxy]-3-methyl-benzonitrile (1.50 g, crude) as a yellow oil which was used in the next step without further purification. LCMS(ESI): $[M+H]^+$ m/z: calcd 289.0; found 289.0.

Step ii: Data for 5-(4-cyano-2-methylphenoxy)pyridin-3-ylboronic acid

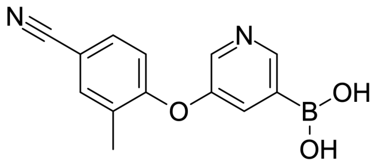

Potassium acetate (1.02 g, 10.4 mmol) was added to a solution of 4-[(5-bromo-3-pyridyl)oxy]-3-methyl-benzonitrile (1.50 g, 5.19 mmol) in dioxane (40 mL). The resulting mixture was stirred at ambient temperature for 5 min. 4,4,5,5-tetramethyl-2-(4,4,5,5-tetramethyl-1,3,2-dioxaborolan-2-yl)-1,3,2-dioxaborolane (1.32 g, 5.19 mmol) and bis(diphenylphosphino) ferrocene]dichloropalladium(II)-DCM (212 mg, 259 μmol) were added to the mixture under argon atmosphere. The resulting mixture was stirred with a reflux condenser under argon atmosphere at 90 °C for 15 hrs.. The resulting mixture was cooled to room temperature and concentrated under reduced pressure. Water (30 mL) was added to the residue and then resulting mixture was extracted with DCM (3×20 mL). The combined organic layers were dried over anhydrous sodium sulfate and filtered. The filtrate was concentrated under reduced pressure to afford 5-(4-cyano-2-methylphenoxy)pyridin-3-ylboronic acid (2.00 g, crude) as a brown solid which was used in the next step without further purification. LCMS(ESI): $[M+H]^+$ m/z: calcd 255.10; found 255.0.

Step iii: Data for methyl 5-[5-(4-cyano-2-methyl-phenoxy)-3 pyridyl] isoxazole-3-carboxylate

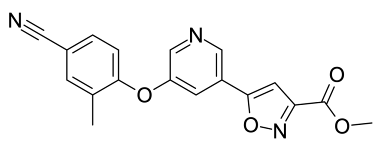

Sodium carbonate (631 mg, 5.95 mmol) was added to a solution of 5-(4-cyano-2-methylphenoxy)pyridin-3-ylboronic acid (1.00 g, crude) in dioxane (19.8 mL) and water (3.96 mL). The resulting mixture was stirred at ambient temperature for 5 min. Methyl 5-bromoisoxazole-3-carboxylate (613 mg, 2.97 mmol) and bis(diphenylphosphino) ferrocene]dichloropalladium(II)-DCM (122 mg, 149 μmol) were added to the mixture under argon atmosphere. The resulting mixture was stirred with a reflux condenser under argon atmosphere at 90 °C for 15 hrs. The reaction mixture was cooled to room temperature and concentrated under reduced pressure. The residue was subjected to HPLC (0-1-5 min., 35-35-60% water – ACN, flow: 30 mL/min, column: Chromatorex 18 SMB100-5T 100×19 mm, 5 μm) to afford methyl 5-[5-(4-cyano-2-methyl-phenoxy)-3-pyridyl]isoxazole-3-carboxylate (53.2 mg, 159 μmol, 5.33% yield) as a yellow solid which was used in the next step without further purification. LCMS(ESI): $[M+H]^+$ m/z: calcd 336.1; found 336.2.

Step iv: Data for 5-[5-(4-cyano-2-methyl-phenoxy)-3-pyridyl]-N,N-dimethyl-isoxazole-3-carboxamide

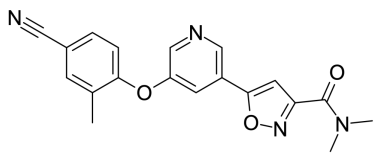

Dimethylamine solution (40% in water, 3.33 mL) was added in one portion to a solution of methyl 5-[5-(4-cyano-2-methyl-phenoxy)-3-pyridyl]isoxazole-3-carboxylate (30.0 mg, 89.5 μmol) in THF (700 μL). The resulting mixture was stirred at 65 °C for 16 hr. The mixture was cooled to room temperature and concentrated under reduced pressure to afford 5-[5-(4-cyano-2-methyl-phenoxy)-3-pyridyl]-N,N-dimethyl-isoxazole-3-carboxamide (30.0 mg, 86.1 μmol, 96.3% yield) as a yellow solid which was used in the next step without further purification. LCMS(ESI): $[M+H]^+$ m/z: calcd 349.13; found 349.0.

Step v: Data for 5-[5-[4-[(Z)-N'-hydroxycarbamimidoyl]-2-methyl-phenoxy]-3-pyridyl]-N,N-dimethyl-isoxazole-3-carboxamide

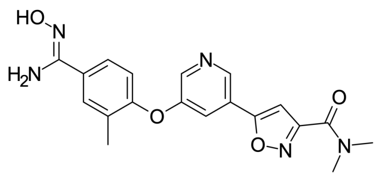

Hydroxylamine hydrochloride (18.0 mg, 258 μmol) was added in one portion to a solution of 5-[5-(4-cyano-2-methyl-phenoxy)-3-pyridyl]-N,N-dimethyl-isoxazole-3-carboxamide (50.0 mg, 144 μmol) in EtOH (10 mL). The resulting mixture was stirred at 70 °C for 15 hr. The reaction mixture was cooled to room temperature and concentrated under reduced pressure to afford 5-[5-[4-[(Z)-N'-hydroxycarbamimidoyl]-2-methyl-phenoxy]-3-pyridyl]-N,N-dimethyl-isoxazole-3-carboxamide (100 mg, crude) as a brown oil which was used in the next step without further purification. LCMS(ESI): $[M+H]^+$ m/z: calcd 382.15; found 382.2.

Step vi: Data for N,N-dimethyl-5-[5-[2-methyl-4-[5-(trifluoromethyl)-1,2,4-oxadiazol-3-yl]phenoxy]-3-pyridyl]isoxazole-3-carboxamide (Compound 32)

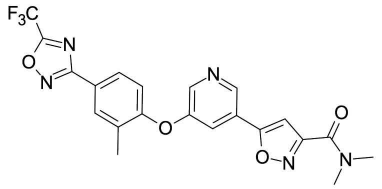

Trifluoroacetic anhydride (82.6 mg, 393 μmol, 54.5 μL) was added a stirred solution of 5-[5-[4-[(Z)-N'-hydroxycarbamimidoyl]-2-methyl-phenoxy]-3-pyridyl] -N,N-dimethyl-isoxazole-3-carboxamide (100 mg, 262 μmol) in THF (10 mL) at room temperature to. The resulting mixture was stirred at room temperature for 15 hr. The reaction mixture was concentrated under reduced pressure and quenched with an aqueous solution of $NaHCO_3$ (10 mL). The aqueous layer was separated and extracted with DCM (2×30 mL). The combined organic layers were washed with water (10 mL), brine (10 mL), dried over anhydrous sodium sulfate and concentrated under reduced pressure. The residue was subjected to HPLC (0-1-5 min., 45-45-70% water – ACN, flow: 30 mL/min, column: Chromatorex 18 SMB100-5T 100×19 mm, 5 μm)

to afford N,N-dimethyl-5-[5-[2-methyl-4-[5-(trifluoromethyl)-1,2,4-oxadiazol-3-yl]phenoxy]-3-pyridyl]isoxazole-3-carboxamide (12.8 mg, 27.9 μmol, 10.6% yield) as a beige solid. ^{1}H NMR (500 MHz, CD_3OD) δ_H 2.40 (s, 3H), 3.13 (s, 3H), 3.24 (s, 3H), 7.16 (d, 1H), 7.20 (s, 1H), 7.91 (t, 1H), 8.02 (dd, 1H), 8.14 (s, 1H), 8.45 (d, 1H), 8.88 (d, 1H). LCMS(ESI): $[M+H]^+$ m/z: calcd 460.13; found 460.0.

Synthesis of N,N-dimethyl-5-(3-(2-methyl-4-(5-(trifluoromethyl)-1,2,4-oxadiazol-3-yl)phenoxy)pyrrolidin-1-yl)isoxazole-3-carboxamide (Enantiomers 33 and 34)

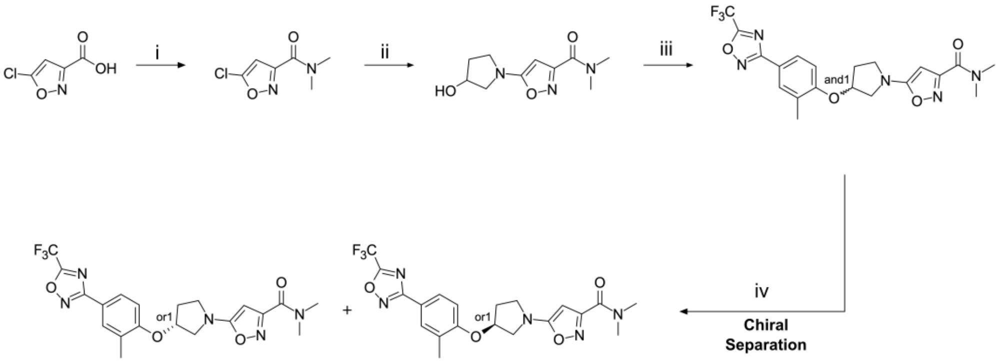

Scheme S9: Reagents and conditions: (i) CDI, N-methylmethanamine, triethylamine, RT, 16h (ii) pyrrolidin-3-ol, K_2CO_3, DMF, 90 °C, 16h (iii) 2-methyl-4-[5-(trifluoromethyl)-1,2,4-oxadiazol-3-yl]phenol, DIAD, PPh_3, THF, RT, 24h (iv) chiral HPLC

Step i: Data for 5-chloro-N,N-dimethyl-isoxazole-3-carboxamide

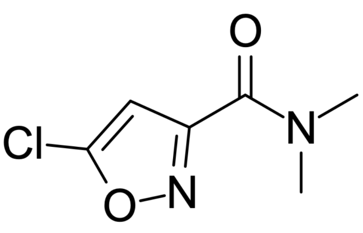

CDI (687 mg, 4.24 mmol) was added to a solution of 5-chloroisoxazole-3-carboxylic acid (500 mg, 3.39 mmol) in THF (5.0 mL) at room temperature. The resulting mixture was stirred at room temperature for 2 hr. Et_3N (567 μL) and N-methylmethanamine (153 mg, 1.87 mmol, 235 μL, HCl) were added to the mixture. The resulting mixture was

stirred at room temperature for 16 hr. The reaction mixture was concentrated under reduced pressure. The residue was subjected to reverse phase HPLC (0-1.3-5.3 min., 20-20-70% water – MeOH, +0.1% vol. of 25% aq. NH_3, flow: 30 mL/min, column: Chromatorex 18 SMB100-5T 100×19 mm, 5 μm) to afford 5-chloro-N,N-dimethyl-isoxazole-3-carboxamide (100 mg, 573 μmol, 16.9% yield) as a brown oil. LCMS(ESI): $[M+H]^+$ m/z: calcd 175.02; found 175.0.

Step ii: Data for 5-(3-hydroxypyrrolidin-1-yl)-N,N-dimethyl-isoxazole-3-carboxamide

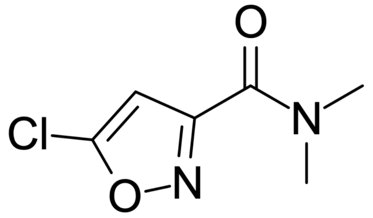

K_2CO_3 (158 mg, 1.15 mmol) was added to a stirred solution of 5-chloro-N,N-dimethyl-isoxazole-3-carboxamide (100 mg, 573 μmol) and pyrrolidin-3-ol (74.9 mg, 859 μmol, 71.4 μL) in DMF (4.0 mL) at room temperature. The resulting mixture was stirred at 90 °C for 16 hr. The reaction mixture was cooled to room temperature and filtered. The filtrate was concentrated under reduced pressure. The residue was subjected to reverse phase HPLC (0-1.3-5.3 min., 10-10-60% water – MeOH, flow: 30 mL/min, column: Chromatorex 18 SMB100-5T 100×19 mm, 5 μm) to afford 5-(3-hydroxypyrrolidin-1-yl)-N,N-dimethyl-isoxazole-3-carboxamide (76.0 mg, 337 μmol, 58.9% yield) as a yellow oil. ^{1}H NMR (500 MHz, dmso) δ_H 1.85 – 1.92 (m, 1H), 1.96 – 2.05 (m, 1H), 2.95 (s, 3H), 3.05 (s, 3H), 3.22 (d, 1H), 3.38 – 3.49 (m, 3H), 4.36 – 4.40 (m, 1H), 5.16 (s, 1H). LCMS(ESI): $[M+H]^+$ m/z: calcd 226.13; found 226.2.

Step iii: Data for N,N-dimethyl-5-[3-[2-methyl-4-[5-(trifluoromethyl)-1,2,4-oxadiazol-3-yl]phenoxy]pyrrolidin-1-yl]isoxazole-3-carboxamide

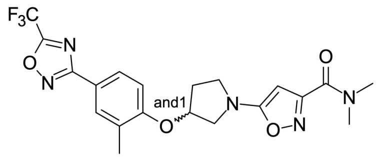

DIAD (75.1 mg, 371 μmol, 73.1 μL) and PPh_3 (97.4 mg, 371 μmol) were to a stirred solution of 5-(3-hydroxypyrrolidin-1-yl)-N,N-dimethyl-isoxazole-3-carboxamide (76.0 mg, 337 μmol) and 2-methyl-4-[5-(trifluoromethyl)-1,2,4-oxadiazol-3-yl]phenol (90.6 mg, 371 μmol) in THF (5.0 mL) added at room temperature. The resulting mixture was stirred at room temperature for 24 hr. The reaction mixture was concentrated under reduced pressure. The residue was subjected to reverse phase HPLC (0-1.3-6.3 min., 50-55-95% water – MeOH, flow: 30 mL/min, column: Chromatorex 18 SMB100-5T 100×19 mm, 5 μm) to afford N,N-dimethyl-5-[3-[2-methyl-4-[5-(trifluoromethyl)-1,2,4-oxadiazol-3-yl]phenoxy]pyrrolidin-1-yl]isoxazole-3-carboxamide (50.0 mg, 111 μmol, 32.8% yield) as a colorless gum. LCMS(ESI): $[M+H]^+$ m/z: calcd 452.17; found 452.0.

Step iv: Chiral resolution

N,N-Dimethyl-5-[3-[2-methyl-4-[5-(trifluoromethyl)-1,2,4-oxadiazol-3-yl]phenoxy]pyrrolidin-1-yl]isoxazole-3-carboxamide (50.0 mg, 114 μmol) was subjected to chiral HPLC (column: CHIRALPAK IA (250×20 mm, 5 μm); eluent: Hexane-IPA-MeOH, 50:25:25; flow: 13 mL/min) to afford **33** (14.0 mg, 31.9 μmol, 28.0% yield) and (**34** (15.0 mg, 34.1 μmol, 30.0% yield) as yellow oils.

Data for (R,or1)-N,N-dimethyl-5-(3-(2-methyl-4-(5-(trifluoromethyl)-1,2,4-oxadiazol-3-yl)phenoxy)pyrrolidin-1-yl)isoxazole-3-carboxamide (Compound 33)

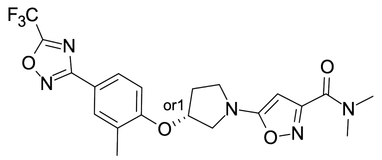

Analytical data: ^{1}H NMR (500 MHz, dmso) δ_H 2.16 (s, 3H), 2.19 – 2.26 (m, 1H), 2.29 – 2.40 (m, 1H), 2.94 (s, 3H), 3.03 (s, 3H), 3.49 – 3.62 (m, 3H), 3.78 (dd, 1H), 5.26 (s, 1H), 5.28 – 5.32 (m, 1H), 7.24 (d, 1H), 7.86 (s, 1H), 7.89 (d, 1H).

LCMS(ESI): $[M+H]^+$ m/z: calcd 452.17; found 452.0. Analytical chiral HPLC (column: Chiralpak IA (250 × 4.6 mm, 5 μm; mobile phase: Hexane-IPA-MeOH, 50:25:25; flow 0.6 mL/min.): Rt 22.09 min.

Data for (S,or1)-N,N-dimethyl-5-(3-(2-methyl-4-(5-(trifluoromethyl)-1,2,4-oxadiazol-3-yl)phenoxy)pyrrolidin-1-yl)isoxazole-3-carboxamide (Compound 34)

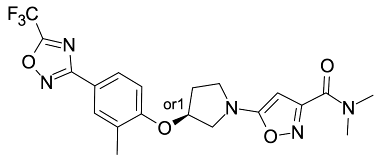

Analytical data: ^{1}H NMR (500 MHz, dmso) δ_H 2.16 (s, 3H), 2.19 – 2.26 (m, 1H), 2.29 – 2.40 (m, 1H), 2.94 (s, 3H), 3.03 (s, 3H), 3.49 – 3.62 (m, 3H), 3.78 (dd, 1H), 5.26 (s, 1H), 5.28 – 5.32 (m, 1H), 7.24 (d, 1H), 7.86 (s, 1H), 7.89 (d, 1H). LCMS(ESI): $[M+H]^+$ m/z: calcd 452.17; found 452.0. Analytical chiral HPLC (column: Chiralpak IA (250 × 4.6 mm, 5 μm; mobile phase: Hexane-IPA-MeOH, 50:25:25; flow 0.6 mL/min.): Rt 34.50 min.

3-[3-methyl-4-[3-(1,3,4-oxadiazol-2-yl)propoxy]phenyl]-5-(trifluoromethyl)-1,2,4-oxadiazole (Compound 35)

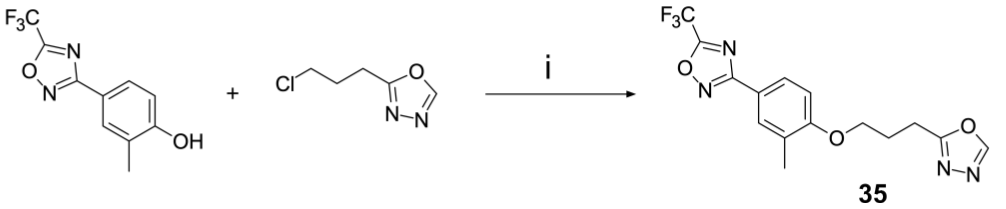

Scheme S10: Reagents and conditions: (i) ^{t}BuOK, NaI, DMF, RT, 24h

Step i: Data for 3-[3-methyl-4-[3-(1,3,4-oxadiazol-2-yl)propoxy]phenyl]-5-(trifluoromethyl)-1,2,4-oxadiazole (Compound 35)

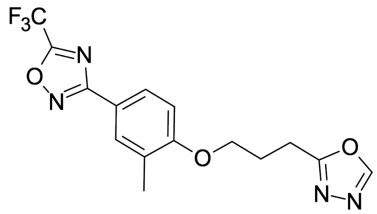

NaI (93.0 mg, 620 μmol), ^{t}BuOK (83.5 mg, 744 μmol) and 2-(3-chloropropyl)-1,3,4-oxadiazole (100 mg, 682 μmol) were added sequentially to a solution of 2-methyl-4-[5-(trifluoromethyl)-1,2,4-oxadiazol-3-yl]phenol (151 mg, 620 μmol) in DMF (1.0 mL). The resulting mixture was stirred at room temperature for 24 hr. The reaction mixture was subjected to HPLC (0-1.2-6 min, 30-30-80% water+FA (0.1% vol.) - ACN+FA (0.1% vol.); flow: 30 mL/min, column: Chromatorex 18 SMB100-5T, 100×19 mm, 5 μm) to afford 3-[3-methyl-4-[3-(1,3,4-oxadiazol-2-yl)propoxy]phenyl]-5-(trifluoromethyl)-1,2,4-oxadiazole (2.20 mg, 5.90 μmol, 0.95% yield) as a brown solid. ^{1}H NMR (500 MHz, CDCl3) δ_H 2.26 (s, 3H), 2.40 (p, 2H), 3.17 (t, 2H), 4.18 (t, 2H), 6.90 (d, 1H), 7.89 (s, 1H), 7.92 (d, 1H), 8.36 (s, 1H). LCMS(ESI): $[M+H]^+$ m/z: calcd 355.11; found 355.0.

Synthesis of 2-methyl-5-[3-[2-methyl-4-[5-(trifluoromethyl)-1,2,4-oxadiazol-3-yl]phenoxy]propyl]-1,3,4-oxadiazole (Compound 36)

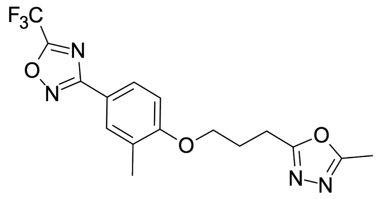

36 was synthesised by a route similar to Compound **2**: DIAD (99.4 mg, 492 μmol) was added dropwise to a stirred solution of 3-(5-methyl-1,3,4-oxadiazol-2-yl)propan-1-ol (58.2 mg, 410 μmol), 2-methyl-4-[5-(trifluoromethyl)-1,2,4-oxadiazol-3-yl]phenol (100 mg, 410 μmol) and PPh_3 (129 mg, 492 μmol) in anhydrous THF (5.0 mL). The resulting mixture was stirred at room temperature for 16 hr. The reaction mixture was concentrated under reduced pressure. The residue was subjected to HPLC (0-1.5-6 min, 30-30-85% water+FA (0.1% vol.) - ACN+FA (0.1% vol.); flow: 30 mL/min, column: Chromatorex 18 SMB100-5T, 100×19 mm, 5 μm) to afford 2-methyl-5-[3-[2-methyl-4-[5-(trifluoromethyl)-1,2,4-oxadiazol-3-yl]phenoxy]propyl]-1,3,4-oxadiazole (45.0 mg, 116 μmol, 28.3% yield) as a yellow gum. ^{1}H NMR (500 MHz, dmso) δ_H 2.15 – 2.24 (m, 5H), 2.42 (s, 3H), 3.01 (t, 2H), 4.16 (t, 2H), 7.13 (d, 1H), 7.83 (d, 1H), 7.87 (dd, 1H). LCMS(ESI): $[M+H]^+$ m/z: calcd 369.13; found 369.2.

Synthesis of 2-(difluoromethyl)-5-[3-[2-methyl-4-[5-(trifluoromethyl)-1,2,4-oxadiazol-3-yl]phenoxy]propyl]-1,3,4-oxadiazole (Compound 37)

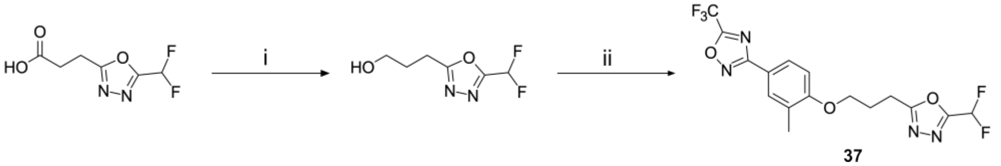

Scheme S11: Reagents and conditions: (i) borane dimethyl sulfide complex, THF, RT, 2h (ii) 2-methyl-4-[5-(trifluoromethyl)-1,2,4-oxadiazol-3-yl]phenol, PPh_3, DIAD, THF, RT, 24h

Step i: Data for 3-[5-(difluoromethyl)-1,3,4-oxadiazol-2-yl]propan-1-ol

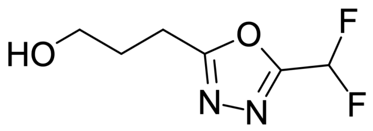

Borane dimethyl sulfide complex (158 mg, 2.08 mmol, 198 μL) was added in one portion to a stirred solution of 3-[5-(difluoromethyl)-1,3,4-oxadiazol-2-yl]propanoic acid (200 mg, 1.04 mmol) in THF (10 mL). The resulting mixture was stirred at room temperature for 2 hr. The reaction mixture was diluted with MeOH (2.0 mL), heated for 10 min and concentrated under reduced pressure. The residue was subjected to HPLC (0-1-5 min., 5-5-45% water – MeOH, flow: 30 mL/min, column: Chromatorex 18 SMB100-5T 100×19 mm, 5 μm) to afford 3-[5-(difluoromethyl)-1,3,4-oxadiazol-2-yl]propan-1-ol (24.6 mg, 138 μmol, 13.3% yield) as a white oil. LCMS(ESI): $[M+H]^+$ m/z: calcd 179.07; found 179.0.

Step ii: Data for 2-(difluoromethyl)-5-[3-[2-methyl-4-[5-(trifluoromethyl)-1,2,4-oxadiazol-3-yl]phenoxy]propyl]-1,3,4-oxadiazole (Compound 37)

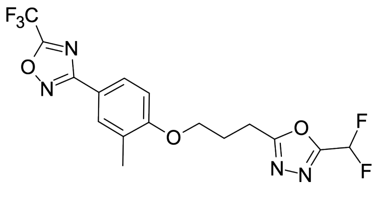

PPh_3 (43.5 mg, 166 μmol), DIAD (33.5 mg, 166 μmol) and 2-methyl-4-[5-(trifluoromethyl)-1,2,4-oxadiazol-3-yl]phenol (33.7 mg, 138 μmol) were added to a solution of 3-[5-(difluoromethyl)-1,3,4-oxadiazol-2-yl]propan-1-ol (24.6 mg, 138 μmol) in THF (5.0 mL). The resulting mixture was stirred at room temperature for 15 hr. The reaction mixture was subjected to HPLC (0-5 min., 40-90% water – ACN, flow: 30 mL/min, column: Chromatorex 18A31 SMB100-5T 100×19 mm, 5 μm) to afford 2-(difluoromethyl)-5-[3-[2-methyl-4-[5-(trifluoromethyl)-1,2,4-oxadiazol-3-yl]phenoxy]propyl]-1,3,4-oxadiazole (27.0 mg, 66.8 μmol, 48.4% yield) as a white solid. ^{1}H NMR (500 MHz, dmso) δ_H 2.15 (s, 3H), 2.26 (p, 2H), 3.16 (t, 2H), 4.19 (t, 2H),

7.13 (d, 1H), 7.45 (t, 1H, CHF2), 7.83 (d, 1H), 7.85 – 7.89 (m, 1H). LCMS(ESI): $[M+H]^+$ m/z: calcd 405.11; found 405.2.

Synthesis of 2-[3-[2-methyl-4-[5-(trifluoromethyl)-1,2,4-oxadiazol-3-yl]phenoxy]propyl]-5-(trifluoromethyl)-1,3,4-oxadiazole (Compound 38)

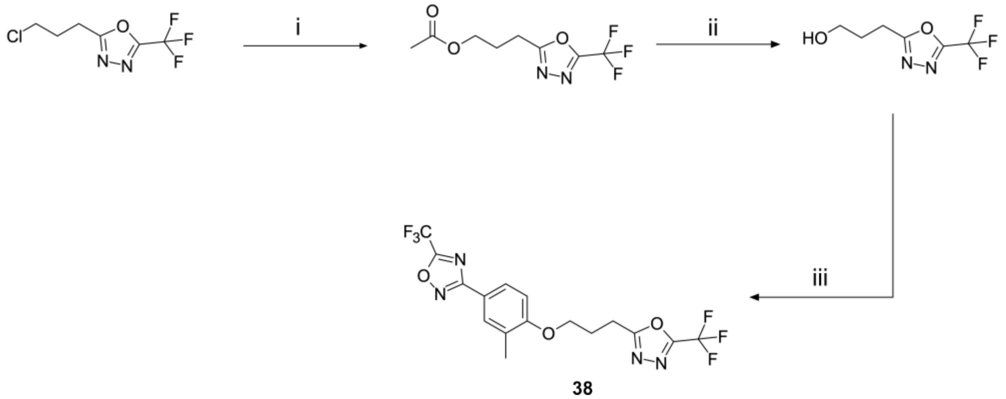

Scheme S12: Reagents and conditions: (i) KOAc, DMF, 60 °C, 16h (ii) NaOH, EtOH, H_2O, THF, 0 °C to RT, 16h (iii) 2-methyl-4-[5-(trifluoromethyl)-1,2,4-oxadiazol-3-yl]phenol, PPh_3, DIAD, THF, RT, 36h

Step i: Data for 3-[5-(trifluoromethyl)-1,3,4-oxadiazol-2-yl]propyl acetate

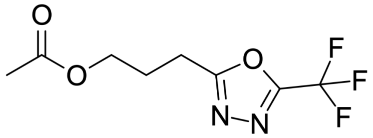

AcOK (1.14 g, 11.7 mmol) was added to a solution of 2-(3-chloropropyl)-5-(trifluoromethyl)-1,3,4-oxadiazole (500 mg, 2.33 mmol) in DMF (3.0 mL). The resulting mixture was stirred at 60 °C for 16 hr. The reaction mixture was cooled to room temperature, quenched with water (10 mL) and extracted with EtOAc (3×20 mL). The combined organic layers were washed with brine, dried over anhydrous sodium sulfate and concentrated under reduced pressure to afford 3-[5-(trifluoromethyl)-1,3,4-oxadiazol-2-

yl]propyl acetate (400 mg, 1.68 mmol, 72.1% yield) as a yellow oil which was used in the next step without further purification. LCMS(ESI): $[M+H]^+$ m/z: calcd 239.07; found 239.2.

Step ii: Data for 3-[5-(trifluoromethyl)-1,3,4-oxadiazol-2-yl]propan-1-ol

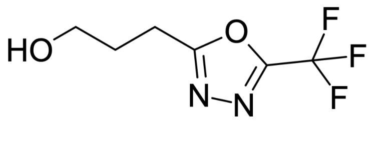

A solution of NaOH (92.4 mg, 2.31 mmol) in EtOH (2.0 mL) and water (500 μL) was added to a solution of 3-[5-(trifluoromethyl)-1,3,4-oxadiazol-2-yl]propyl acetate (500 mg, 2.10 mmol) in THF (2.0 mL) at 0 °C. The resulting mixture was stirred at room temperature overnight. The reaction mixture was concentrated under reduced pressure. The residue was extracted with EtOAc (2×10 mL). The combined organic layers were dried over anhydrous sodium sulfate and concentrated under reduced pressure to afford 3-[5-(trifluoromethyl)-1,3,4-oxadiazol-2-yl]propan-1-ol (150 mg, 765 μmol, 36.4% yield) as a yellow oil which was used in next step without further purification. LCMS(ESI): $[M+H]^+$ m/z: calcd 197.06; found 197.0.

Step iii: Data for 2-[3-[2-methyl-4-[5-(trifluoromethyl)-1,2,4-oxadiazol-3-yl]phenoxy]propyl]-5-(trifluoromethyl)-1,3,4-oxadiazole (Compound 38)

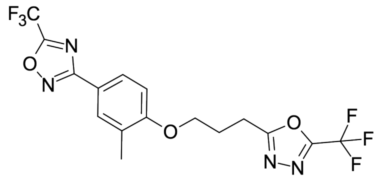

DIAD (186 mg, 918 μmol, 181 μL) was added dropwise to a stirred solution of 3-[5-(trifluoromethyl)-1,3,4-oxadiazol-2-yl]propan-1-ol (150 mg, 765 μmol), 2-methyl-4-[5-(trifluoromethyl)-1,2,4-oxadiazol-3-yl]phenol (187 mg, 765 μmol) and PPh_3 (241 mg, 918 μmol) in anhydrous THF (1.47 mL). The resulting mixture was stirred at room temperature for 36 hr. The reaction mixture was cooled to room temperature and concentrated under reduced pres-

sure. The residue was subjected to HPLC (0-1.5-5.3 min., 50-50-80% water – ACN, +0.1% vol. of 25% aq. NH_3, flow: 30 mL/min, column: SunFire 100×19 mm, 5 μm) to afford 2-[3-[2-methyl-4-[5-(trifluoromethyl)-1,2,4-oxadiazol-3-yl]phenoxy]propyl]-5-(trifluoromethyl)-1,3,4-oxadiazole (2.00 mg, 4.74 μmol, 0.62% yield) as a colorless gum. LCMS(ESI): $[M+H]^+$ m/z: calcd 423.1; found 423.0.

Synthesis of 3-[3-methyl-4-[3-(5-methyl-4H-1,2,4-triazol-3-yl)propoxy] phenyl]-5-(trifluoromethyl)-1,2,4-oxadiazole (Compound 39)

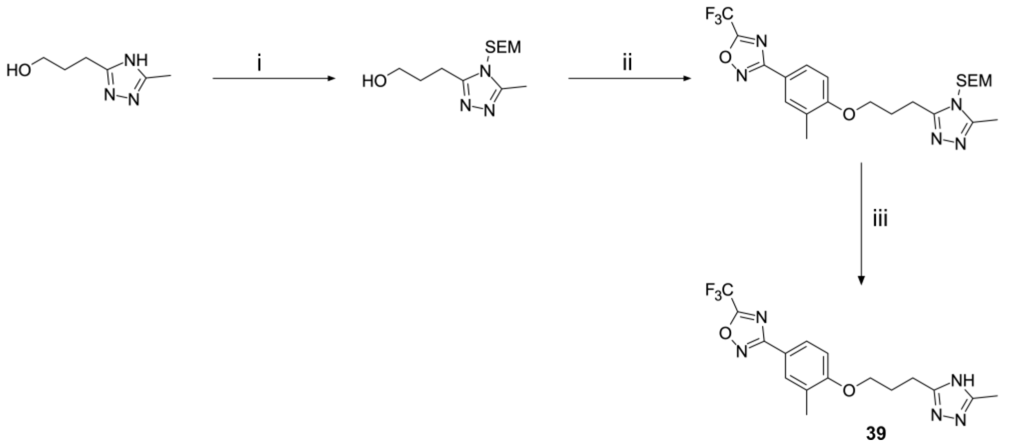

Scheme S13: Reagents and conditions: (i) SEM-Cl, Et_3N, DMF, RT, 16h (ii) 2-methyl-4-[5-(trifluoromethyl)-1,2,4-oxadiazol-3-yl]phenol, PPh_3, DIAD, THF, 0 °C to RT, 16h (iii) TFA, DCM, 0 °C to RT, 16h

Step i: Data for 3-[5-methyl-4-(2-trimethylsilylethoxymethyl)-1,2,4-triazol-3-yl]propan-1-ol

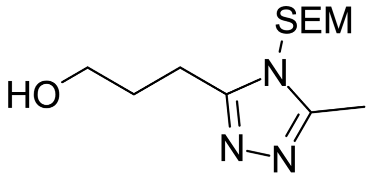

3-(5-Methyl-4H-1,2,4-triazol-3-yl)propan-1-ol (400 mg, 2.83 mmol), 2-(chloromethoxy)ethyl-trimethyl-silane (543 mg, 3.26 mmol) and Et_3N (287 mg, 2.83 mmol, 395 μL) were mixed in DMF (10 mL). The resulting mixture was

stirred at room temperature for 16 hr. The reaction mixture was diluted with DCM (20 mL) and washed with water (20 mL). The organic layer was separated and concentrated under reduced pressure to afford 3-[5-methyl-4-(2-trimethylsilylethoxymethyl)-1,2,4-triazol-3-yl]propan-1-ol (300 mg, 674 μmol, 23.8% yield) as a yellow oil which was used in the next step without further purification. LCMS(ESI): $[M+H]^+$ m/z: calcd 272.18; found 272.2.

Step ii: Data for trimethyl-[2-[[3-methyl-5-[3-[2-methyl-4-[5-(trifluoromethyl)-1,2,4-oxadiazol-3-yl]phenoxy]propyl]-1,2,4-triazol-4-yl]methoxy]ethyl]silane

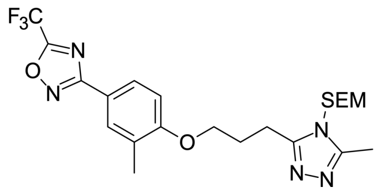

DIAD (72.4 mg, 358 μmol, 70.5 μL) was added dropwise to a stirred solution of 2-methyl-4-[5-(trifluoromethyl)-1,2,4-oxadiazol-3-yl]phenol (72.9 mg, 298 μmol), 3-[5-methyl-4-(2-trimethylsilylethoxymethyl)-1,2,4-triazol-3-yl]propan-1-ol (300 mg, 298 μmol) and PPh_3 (93.9 mg, 358 μmol) in anhydrous THF (10 mL) at 0 °C. The resulting mixture was stirred at room temperature for 16 hr. The resulting mixture was concentrated under reduced pressure. The residue was partitioned between EtOAc (25 mL) and water (7 mL). The organic layer was separated, dried over anhydrous sodium sulfate and concentrated under reduced pressure to afford trimethyl-[2-[[3-methyl-5-[3-[2-methyl-4-[5-(trifluoromethyl)-1,2,4-oxadiazol-3-yl]phenoxy]propyl]-1,2,4-triazol-4-yl]methoxy]ethyl]silane (600 mg, 265 μmol, 88.9% yield) as a brown oil which was used in next step without further purification. LCMS(ESI): $[M+H]^+$ m/z: calcd 498.22; found 498.0.

Step iii: Data for 3-[3-methyl-4-[3-(5-methyl-4H-1,2,4-triazol-3-yl)propoxy] phenyl]-5-(trifluoromethyl)-1,2,4-oxadiazole (Compound 39)

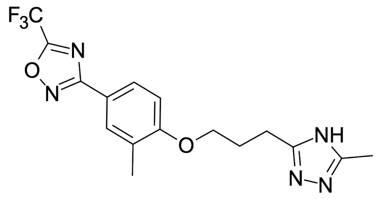

TFA (182 mg, 1.59 mmol, 122 μL) was added to a solution of trimethyl-[2-[[3-methyl-5-[3-[2-methyl-4-[5-(trifluoromethyl)-1,2,4-oxadiazol-3-yl]phenoxy]propyl]-1,2,4-triazol-4-yl]methoxy]ethyl]silane (600 mg, 265 μmol) in DCM (10 mL) at 0 °C. The resulting mixture was stirred at room temperature for 16 hr. The reaction mixture was concentrated under reduced pressure. The residue was subjected to HPLC (0-1.3-5.3 min., 40-40-65% water – ACN, flow: 30 mL/min, column: Chromatorex 18 SMB100-5T 100×19 mm, 5 μm) to afford 3-[3-methyl-4-[3-(5-methyl-4H-1,2,4-triazol-3-yl)propoxy]phenyl]-5-(trifluoromethyl)-1,2,4-oxadiazole (22.5 mg, 58.2 μmol, 21.9% yield) as a white solid. ^{1}H NMR (500 MHz, dmso) δ_H 2.05 - 2.26 (m, 8H), 2.69 – 2.87 (m, 2H), 4.11 (t, 2H), 7.11 (d, 1H), 7.82 – 7.88 (m, 2H), 13.15 – 2.27 (m, 1H). LCMS(ESI): $[M+H]^+$ m/z: calcd 368.15; found 368.2.

Synthesis of 3-[3-methyl-4-[3-(1H-pyrazol-4-yl)propoxy]phenyl] -5-(trifluoromethyl)-1,2,4-oxadiazole (Compound 40)

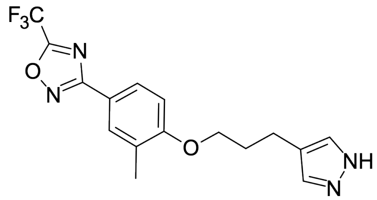

40 was synthesised by a route similar to Compound **2**: DIAD (96.2 mg, 476 μmol, 93.6 μL), PPh_3 (125 mg, 476 μmol) and 2-methyl-4-[5-(trifluoromethyl)-1,2,4-oxadiazol-3-yl]phenol (96.8 mg, 396 μmol) were added to a solution of 3-(1H-pyrazol-4-yl)propan-1-ol (50.0 mg, 396 μmol) in THF (5.0 mL). The resulting mixture was stirred at room temperature for 14 hr. The reaction mixture was subjected to HPLC (0-1-5 min., 55-55-80% water – ACN, flow: 30 mL/min, column:

Chromatorex 18 SMB100-5T 100×19 mm, 5 μm) to afford 3-[3-methyl-4-[3-(1H-pyrazol-4-yl)propoxy]phenyl]-5-(trifluoromethyl)-1,2,4-oxadiazole (38.3 mg, 109 μmol, 27.4% yield) as a beige solid. ^{1}H NMR (500 MHz, dmso) δ_H 1.96 – 2.06 (m, 2H), 2.24 (s, 3H), 2.61 (t, 2H), 4.06 (t, 2H), 7.11 (d, 1H), 7.32 (br. s, 1H), 7.52 (br. s, 1H), 7.82 – 7.89 (m, 2H), 12.52 (br. s, 1H). LCMS(ESI): $[M+H]^+$ m/z: calcd 353.14; found 353.2.

Synthesis of 3-[3-methyl-4-[3-(1-methylpyrazol-4-yl)propoxy]phenyl]-5-(trifluoromethyl)-1,2,4-oxadiazole (Compound 41)

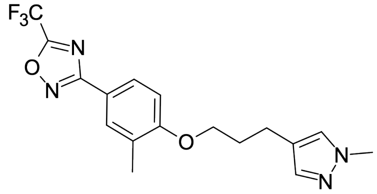

41 was synthesised by a route similar to Compound **2**: 2-Methyl-4-[5-(trifluoromethyl)-1,2,4-oxadiazol-3-yl]phenol (50.0 mg, 205 μmol) was mixed with 3-(1-methylpyrazol-4-yl)propan-1-ol (28.7 mg, 205 μmol) and PPh_3 (80.6 mg, 307 μmol) in dry THF (3.0 mL), after that DEAD (53.5 mg, 307 μmol) was added dropwise to the solution at 0 °C. The resulting mixture was allowed to warm up to room temperature and stirred overnight. The reaction mixture was subjected to HPLC (0-1-6 min., 40-40-90% water – ACN, flow: 60 mL/min, column: XBridge C18 OBD 100×30 mm, 5 μm) to afford 3-[3-methyl-4-[3-(1-methylpyrazol-4-yl)propoxy]phenyl]-5-(trifluoromethyl)-1,2,4-oxadiazole (65.4 mg, 179 μmol, 87.2% yield) as a colorless oil. ^{1}H NMR (500 MHz, dmso) δ_H 1.93 – 2.08 (m, 2H), 2.23 (s, 3H), 2.57 (t, 2H), 3.74 (s, 3H), 4.06 (t, 2H), 7.11 (d, 1H), 7.24 (s, 1H), 7.48 (s, 1H), 7.82 – 7.88 (m, 2H). LCMS(ESI): $[M+H]^+$ m/z: calcd 367.16; found 367.0.

Synthesis of 3-[3-methyl-4-[3-(2-pyridyl)propoxy]phenyl]-5-(trifluoromethyl)-1,2,4-oxadiazole)-1,2,4-oxadiazole (Compound 42)

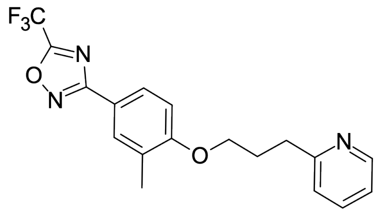

42 was synthesised by a route similar to Compound **2**: PPh_3 (107 mg, 410 μmol) and DIAD (82.8 mg, 410 μmol) were added to a stirred solution of 2-methyl-4-[5-(trifluoromethyl)-1,2,4-oxadiazol-3-yl]phenol (100 mg, 410 μmol) and 3-(2-pyridyl)propan-1-ol (51.1 mg, 372 μmol) in THF (4.0 mL) at room temperature. The resulting mixture was stirred at room temperature for 24 hr. The reaction mixture was concentrated under reduced pressure. The residue was subjected to HPLC (0-1.3-5.3 min., 60-60-85% water – ACN, flow: 30 mL/min, column: Chromatorex 18 SMB100-5T 100×19 mm, 5 μm) to afford 3-[3-methyl-4-[3-(2-pyridyl)propoxy]phenyl]-5-(trifluoromethyl)-1,2,4-oxadiazole (6.60 mg, 18.2 μmol, 4.88% yield) as a white solid. ^{1}H NMR (500 MHz, dmso) δ_H 2.13 – 2.20 (m, 2H), 2.21 (s, 3H), 2.92 (t, 2H), 4.11 (t, 2H), 7.11 (d, 1H), 7.19 (dd, 1H), 7.28 (d, 1H), 7.65 – 7.72 (m, 1H), 7.81 – 7.88 (m, 2H), 8.48 (dd, 1H). LCMS(ESI): $[M+H]^+$ m/z: calcd 364.15; found 364.2.

Synthesis of 3-[3-methyl-4-[3-(3-pyridyl)propoxy]phenyl]-5-(trifluoromethyl)-1,2,4-oxadiazole (Compound 43)

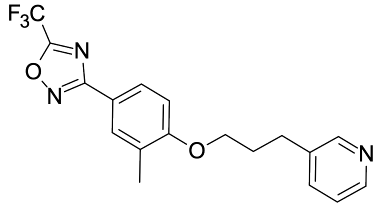

43 was synthesised by a route similar to Compound **2**: DIAD (95.2 mg, 471 μmol, 92.7 μL), PPh_3 (124 mg, 471 μmol) and 3-(3-pyridyl)propan-1-ol (64.6 mg, 471 μmol) were added to a solution of 2-methyl-4-[5-(trifluoromethyl)-1,2,4-oxadiazol-3-yl]phenol (100 mg, 410 μmol) in THF (15.0 mL) at 0 °C. The resulting mixture was stirred at room temperature for 16 hr. The reaction mixture was

concentrated under reduced pressure. The residue was subjected to HPLC (0-1-5 min, 35-35-55% water+FA (0.1% vol.) - ACN+FA (0.1% vol.); flow: 30 mL/min, column: Chromatorex 18 SMB100-5T, 100×19 mm, 5 μm) to afford 3-[3-methyl-4-[3-(3-pyridyl)propoxy]phenyl]-5-(trifluoromethyl)-1,2,4-oxadiazole (20.0 mg, 55.1 μmol, 13.4% yield) as an off-white solid. ^{1}H NMR (500 MHz, dmso) δ_H 1.96 – 2.06 (m, 2H), 2.22 (s, 3H), 2.80 (t, 2H), 4.07 (t, 2H), 7.11 (d, 1H), 7.30 (dd, 1H), 7.63 – 7.69 (m, 1H), 7.82 – 7.87 (m, 2H), 8.39 (dd, 1H), 8.45 (d, 1H). LCMS(ESI): $[M+H]^+$ m/z: calcd 364.15; found 364.2.

Synthesis of 3-[3-methyl-4-[3-(4-pyridyl)propoxy]phenyl]-5-(trifluoromethyl)-1,2,4-oxadiazole (Compound 44)

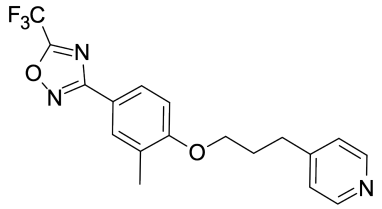

44 was synthesised by a route similar to Compound **2**: 2-Methyl-4-[5-(trifluoromethyl)-1,2,4-oxadiazol-3-yl]phenol (50.0 mg, 205 μmol) was mixed with 3-(4-pyridyl)propan-1-ol (28.1 mg, 205 μmol) and PPh_3 (80.6 mg, 307 μmol) in dry THF (3.0 mL), after that DEAD (53.5 mg, 307 μmol) was added dropwise to the solution at 0 °C. The resulting mixture was allowed to warm up to room temperature and stirred overnight. The reaction mixture was subjected to HPLC (0-1-6 min., 40-40-90% water – ACN, flow: 60 mL/min, column: XBridge C18 OBD 100×30 mm, 5 μm) to afford 3-[3-methyl-4-[3-(4-pyridyl)propoxy]phenyl]-5-(trifluoromethyl)-1,2,4-oxadiazole (45.0 mg, 124 μmol, 60.5% yield) as a beige solid. ^{1}H NMR (500 MHz, dmso) δ_H 2.10 (p, 2H), 2.21 (s, 3H), 2.79 (t, 2H), 4.07 (t, 2H), 7.11 (d, 1H), 7.26 (d, 2H), 7.82 – 7.87 (m, 2H), 8.44 (d, 2H). LCMS(ESI): $[M+H]^+$ m/z: calcd 364.15; found 364.0.

Synthesis of 3-[3-methyl-4-[3-(1-phenyltetrazol-5-yl)propoxy]phenyl]-5-(trifluoromethyl)-1,2,4-oxadiazole (Compound 45)

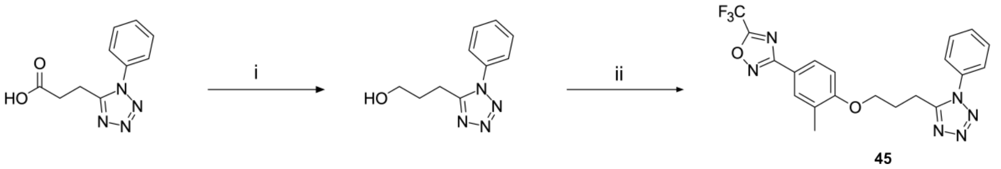

Scheme S14: Reagents and conditions: (i) borane dimethyl sulfide complex, THF, 10 °C to RT, 16h (ii) PPh_3, DIAD, 2-methyl-4-[5-(trifluoromethyl)-1,2,4-oxadiazol-3-yl]phenol, THF, RT, 16h

Step i: Data for 3-(1-phenyltetrazol-5-yl)propan-1-ol

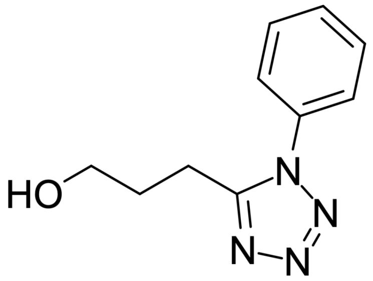

Borane dimethyl sulfide complex (69.6 mg, 917 μmol, 86.9 μL) was added to a pre-cooled to 0 °C stirred solution of 3-(1-phenyltetrazol-5-yl)propanoic acid (100 mg, 458 μmol) in THF (1.0 mL) keeping temperature below 10 °C. The resulting mixture was allowed to warm up to room temperature and stirred for 16 hr. The reaction mixture was concentrated under reduced pressure. The residue was dissolved in MeOH (2.0 mL) and concentrated under reduced pressure again to afford 3-(1-phenyltetrazol-5-yl)propan-1-ol (100 mg, crude) as a yellow oil which was used in the next step without further purification. LCMS(ESI): $[M+H]^+$ m/z: calcd 205.11; found 205.2.

Step ii: Data for 3-[3-methyl-4-[3-(1-phenyltetrazol-5-yl)propoxy]phenyl]-5-(trifluoromethyl)-1,2,4-oxadiazole (Compound 45)

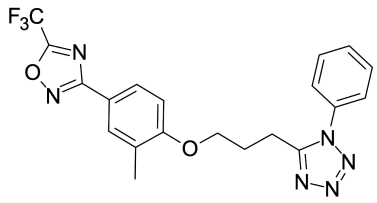

DIAD (218 mg, 1.08 mmol) was added dropwise to a stirred solution of 3-(1-phenyltetrazol-5-yl)propan-1-ol (200 mg, 979 μmol), 2-methyl-4-[5-(trifluoromethyl)-1,2,4-oxadiazol-3-yl]phenol (191 mg, 783 μmol) and PPh_3 (283 mg, 1.08 mmol) in anhydrous THF (1.5 mL). The resulting mixture was stirred at room temperature for 16 hr. The reaction mixture was concentrated under reduced pressure. The residue was subjected to HPLC (0-55 min., 40-80% water – ACN, flow: 30 mL/min, column: PHENYL SMB100-5T 100×19 mm, 5 μm) to afford 3-[3-methyl-4-[3-(1-phenyltetrazol-5-yl)propoxy]phenyl]-5-(trifluoromethyl)-1,2,4-oxadiazole (41.0 mg, 95.3 μmol, 9.73% yield) as a light-yellow solid. ^{1}H NMR (500 MHz, dmso) δ_H 2.06 (s, 3H), 2.19 (p, 2H), 3.10 (t, 2H), 4.10 (t, 2H), 7.06 (d, 1H), 7.56 – 7.66 (m, 5H), 7.80 (s, 1H), 7.84 (d, 1H). LCMS(ESI): $[M+H]^+$ m/z: calcd 431.16; found 431.2.

Synthesis of 5-[3-[(3-ethoxy-1,2-benzoxazol-6-yl)oxy]propyl]-N,N-dimethyl-isoxazole-3-carboxamide

(Compound 46)

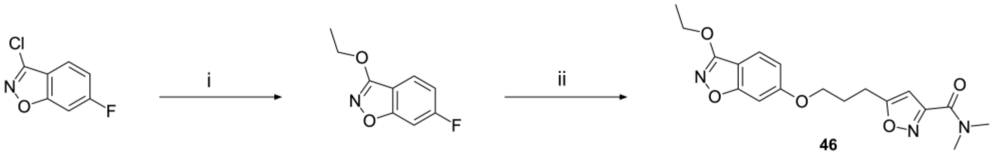

Scheme S15: Reagents and conditions: (i) NaH, EtOH, DMF, RT, 16h (ii) 5-(3-Hydroxypropyl)-N,N-dimethyl-isoxazole-3-carboxamide, NaH, DMF, 0 °C to RT, 16h

Step i: Data for 3-ethoxy-6-fluoro-1,2-benzoxazole

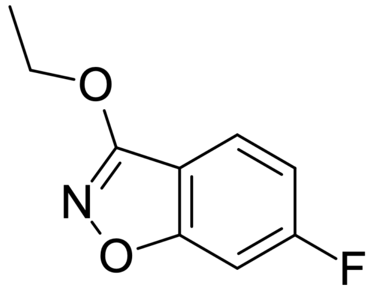

NaH (14.0 mg, 583 μmol, 60% dispersion in mineral oil) was added portionwise to an ice-cooled mixture of 3-Chloro-6-fluoro-1,2-benzoxazole (100 mg, 583 μmol) and EtOH (26.9 mg, 583 μmol, 34.0 μL) in DMF (2.0 mL). The resulting mixture was stirred at room temperature for 16 hr. The reaction mixture was cooled to room temperature to afford a solution of 3-ethoxy-6-fluoro-1,2-benzoxazole (100 mg, 254 μmol, 46% purity by LCMS) in DMF which was used in the next step without work up.

Step ii: Data for 5-[3-[(3-ethoxy-1,2-benzoxazol-6-yl)oxy]propyl]-N,N-dimethyl-isoxazole-3-carboxamide

(Compound 46)

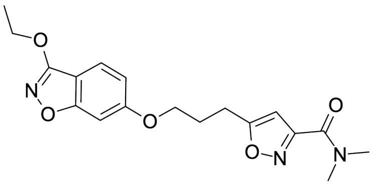

5-(3-Hydroxypropyl)-N,N-dimethyl-isoxazole-3-carboxamide (109 mg, 552 μmol) and NaH (13.3 mg, 552 μmol, 60% dispersion in mineral oil) were added sequentially to a stirred solution

of 3-ethoxy-6-fluoro-1,2-benzoxazole (100 mg, 552 μmol) in DMF (2.0 mL) at 0 °C. The resulting mixture was stirred at room temperature for 16 hr. The reaction mixture was partitioned between EtOAc (20 mL) and water (5.0 mL). The organic layer was separated, washed with water (3×5 mL) and brine (5 mL), dried over anhydrous sodium sulfate and concentrated under reduced pressure. The residue was subjected to HPLC (0-1.3-6 min., 35-35-45% water – ACN, +0.1% vol. of 25% aq. NH_3, flow: 30 mL/min, column: XBridge BEH C18 100×19 mm, 5 μm) to afford a mixture of two regioisomers which was further subjected to chiral HPLC (column: Chromatorex EP2 (100×19 mm, 5 μm), eluent: 95:2.5:2.5 Hexane:IPA:MeOH, flow: 12.0 mL/min) to afford 5-[3-[(3-ethoxy-1,2-benzoxazol-6-yl)oxy]propyl]-N,N-dimethyl-isoxazole-3-carboxamide (3.40 mg, 9.27 μmol, 1.68% yield) as a light-yellow solid. ^{1}H NMR (600 MHz, dmso) δ_H 1.41 (t, 3H), 2.14 (p, 2H), 2.94 – 3.00 (m, 5H), 3.04 (s, 3H), 4.12 (t, 2H), 4.39 (q, 2H), 6.52 (s, 1H), 6.90 (dd, 1H), 7.16 (d, 1H), 7.54 (d, 1H). LCMS(ESI): $[M+H]^+$ m/z: calcd 360.17; found 360.0.

Synthesis of 5-[3-[(3-ethoxy-5-methyl-1,2-benzoxazol-6-yl)oxy]propyl]-N,N-dimethyl-isoxazole-3-carboxamide (Compound 47)

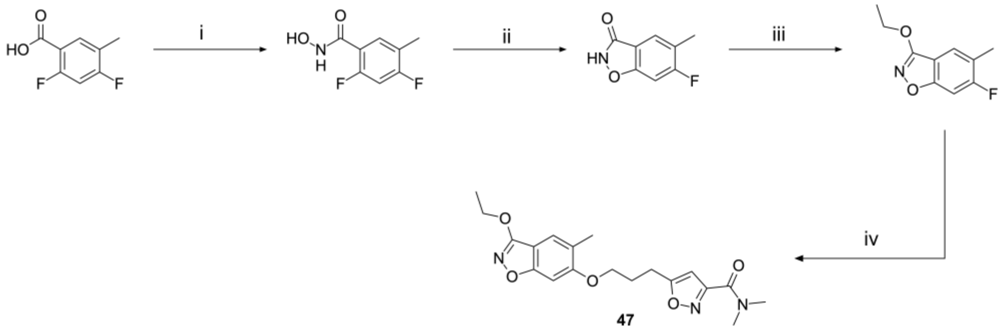

Scheme S16: Reagents and conditions: (i) hydroxylamine, oxalyl chloride, DCM, DMF, 0 °C to RT, 16h (ii) KOH, ^{n}BuOH, 130 °C, 4h (iii) Ag_2O, iodoethane, $CHCl_3$, RT to reflux, 16h (iv) 5-(3-hydroxypropyl)-N,N-dimethyl-isoxazole-3-carboxamide, NaH, DMF, 100 °C, 16h

Step i: Data for 2,4-difluoro-5-methyl-benzenecarbohydroxamic acid

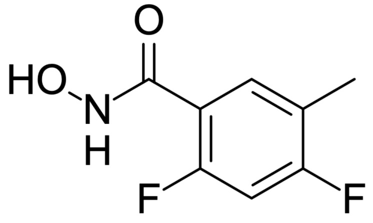

A catalytic amount of DMF and oxalyl chloride (3.48 g, 27.5 mmol, 2.39 mL) were added sequentially to a stirred solution of 2,4-difluoro-5-methyl-benzoic acid (3.15 g, 18.3 mmol) in DCM (50 mL) at 0 °C. The resulting mixture was allowed to warm up to room temperature and stirred overnight. The reaction mixture was concentrated under reduced pressure. The residue was redissolved in a minimum amount of EtOAc. This solution was added dropwise to a vigorously stirring mixture of hydroxylamine (2.54 g, 36.6 mmol, 1.52 mL, HCl), K_2CO_3 (10.1 g, 73.2 mmol), water (80 mL) and EtOAc (160 mL) at 0 °C. The aqueous layer was separated and extracted with EtOAc (80 mL). The combined organic layers were dried over anhydrous sodium sulfate and concentrated under reduced pressure to afford 2,4-difluoro-5-methyl-benzenecarbohydroxamic acid (3.00 g, 16.0 mmol, 87.6% yield) as a brown solid which was used in the next step without further purification. ^{1}H NMR (500 MHz, dmso) δ_H 2.21 (s, 3H), 7.26 (t, 1H), 7.49 (t, 1H), 9.19 (s, 1H), 10.91 (s, 1H). LCMS(ESI): $[M+H]^+$ m/z: calcd 188.05; found 188.0.

Step ii: Data for 6-fluoro-5-methyl-1,2-benzoxazol-3-one

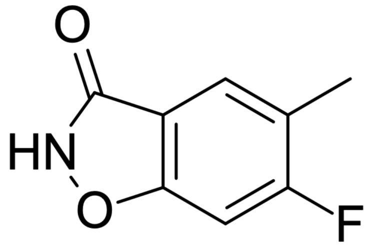

KOH (1.08 g, 19.2 mmol) was added to a stirred solution of 2,4-difluoro-5-methyl-benzenecarbohydroxamic acid (600 mg, 3.21 mmol) in ^{n}BuOH (6.0 mL) at room temperature. The resulting mixture was stirred at 130 °C for 4 hr. The reaction mixture was cooled to room temperature and concentrated under reduced pressure. The residue was diluted with water (10 mL), acidified with hydrochloric acid and extracted with EtOAc (20 mL × 2). The combined organic layers were washed with brine (10 mL), dried over anhydrous sodium sulfate and concentrated under reduced pressure. The residue was subjected to column chromatog-

raphy (ISCO®: CombiFlash; 40 g SiO_2, gradient $CHCl_3$/MTBE with MTBE from 0 95%, flow rate = 40 mL/min, Rf = 8-10 CV.) to afford 6-fluoro-5-methyl-1,2-benzoxazol-3-one (200 mg, 1.20 mmol, 37.3% yield) as a yellow solid. ^{1}H NMR (500 MHz, dmso) δ_H 2.30 (s, 3H), 7.46 (d, 1H), 7.64 (d, 1H), 12.34 (br. s, 1H). LCMS(ESI): $[M+H]^+$ m/z: calcd 168.05; found 168.0.

Step iii: Data for 3-ethoxy-6-fluoro-5-methyl-1,2-benzoxazole

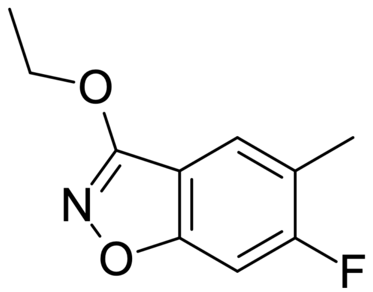

Ag_2O (555 mg, 2.39 mmol) and iodoethane (187 mg, 1.20 mmol, 96.2 μL) were added sequentially to a stirred solution of 6-fluoro-5-methyl-1,2-benzoxazol-3-one (200 mg, 1.20 mmol) in $CHCl_3$ (50 mL) at room temperature. The resulting mixture was stirred under reflux overnight. The reaction mixture was cooled to room temperature and filtered through a thin pad of silica gel. The filtercake was washed several times with $CHCl_3$. The filtrate was concentrated under reduced pressure. The residue was subjected to reverse phase HPLC (0-1-5 min., 35-35-80% water – ACN, flow: 30 mL/min, column: Chromatorex 18 SMB100-5T 100×19 mm, 5 μm) to afford 3-ethoxy-6-fluoro-5-methyl-1,2-benzoxazole (65.0 mg, 333 μmol, 27.8% yield) as a yellow solid. LCMS(ESI): $[M+H]^+$ m/z: calcd 196.09; found 196.0.

Step iv: Data for 5-[3-[(3-ethoxy-5-methyl-1,2-benzoxazol-6-yl)oxy]propyl]-N,N-dimethyl-isoxazole-3-carboxamide (Compound 47)

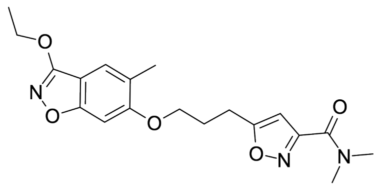

NaH (6.45 mg, 161 μmol, 60% dispersion in mineral oil) was added to a stirred solution of 5-(3-hydroxypropyl)-N,N-dimethyl-isoxazole-3-carboxamide (32.0 mg, 161 μmol)

in DMF (3.0 mL) at room temperature. The mixture was stirred for 15 min then 3-ethoxy-6-fluoro-5-methyl-1,2-benzoxazole (30.0 mg, 154 μmol) was added. The resulting mixture was stirred at 100 °C overnight. The reaction mixture was cooled to room temperature and subjected to preparative HPLC (0-1-7 min., 30-35-60% water+FA (0.1% vol.) - ACN+FA (0.1% vol.); flow: 30 mL/min, column: Chromatorex 18 SMB100-5T 100×19 mm, 5 μm) to afford 5-[3-[(3-ethoxy-5-methyl-1,2-benzoxazol-6-yl)oxy]propyl]-N,N-dimethyl-isoxazole-3-carboxamide (16.5 mg, 44.2 μmol, 28.8% yield) as a white solid. ^{1}H NMR (500 MHz, dmso) δ_H 1.40 (t, 3H), 2.13 – 2.22 (m, 5H), 2.97 (s, 3H), 3.00 (t, 2H), 3.03 (s, 3H), 4.11 (t, 2H), 4.38 (q, 2H), 6.52 (s, 1H), 7.14 (s, 1H), 7.41 (s, 1H). LCMS(ESI): $[M+H]^+$ m/z: calcd 374.19; found 374.2.

Synthesis of 5-[3-[(3-ethoxy-7-methyl-1,2-benzoxazol-6-yl)oxy]propyl]-N,N-dimethyl-isoxazole-3-carboxamide (Compound 48)

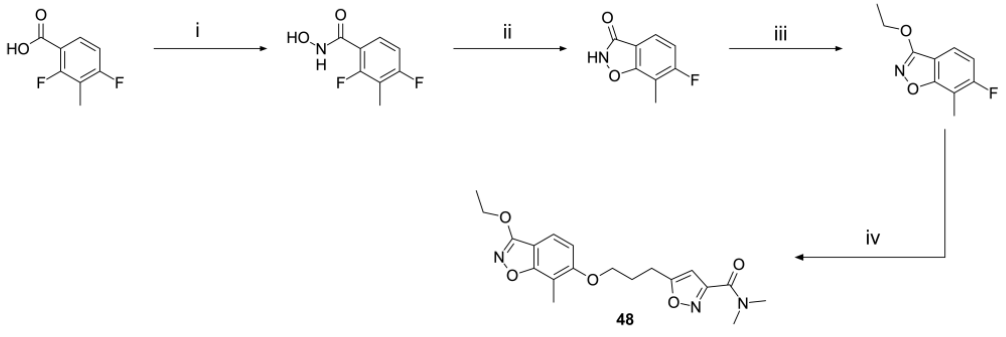

Scheme S17: Reagents and conditions: (i) hydroxylamine, oxalyl chloride, DCM, DMF, 0 °C to RT, 16h (ii) KOH, ^{n}BuOH, 130 °C, 4h (iii) Ag_2O, iodoethane, $CHCl_3$, RT to reflux, 16h (iv) 5-(3-hydroxypropyl)-N,N-dimethyl-isoxazole-3-carboxamide, NaH, DMF, 100 °C, 16h

Step i: Data for 2,4-difluoro-3-methyl-benzenecarbohydroxamic acid

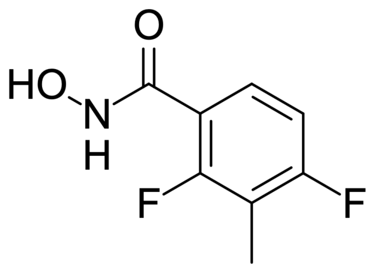

A catalytic amount of DMF and oxalyl chloride (553 mg, 4.36 mmol, 380 μL) were added sequentially to a stirred solution of 2,4-difluoro-3-methyl-benzoic acid (500 mg, 2.90 mmol) in DCM (10 mL) at 0 °C. The resulting mixture was allowed to warm up to room temperature and stirred overnight. The reaction mixture was concentrated under reduced pressure. The residue was redissolved in a minimum amount of EtOAc. This solution was added dropwise to a vigorously stirring mixture of hydroxylamine (404 mg, 5.81 mmol, HCl), K_2CO_3 (1.61 g, 11.6 mmol), water (15 mL) and EtOAc (30 mL) at 0 °C. The aqueous layer was separated and extracted with EtOAc (40 mL). The organic layer was dried over anhydrous sodium sulfate and concentrated under reduced pressure to afford 2,4-difluoro-3-methyl-benzenecarbohydroxamic acid (460 mg, 2.46 mmol, 84.6% yield) as a red solid which was used in the next step without further purification. ^{1}H NMR (500 MHz, dmso) δ_H 2.16 (s, 3H), 7.12 (t, 1H), 7.40 (q, 1H), 9.20 (s, 1H), 10.95 (s, 1H). LCMS(ESI): $[M+H]^+$ m/z: calcd 188.05; found 188.0.

Step ii: Data for 6-fluoro-7-methyl-1,2-benzoxazol-3-one

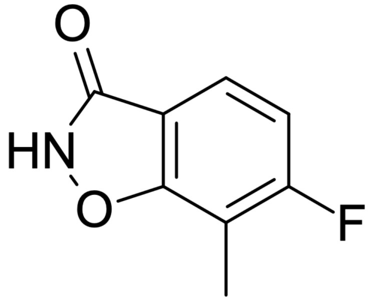

KOH (828 mg, 14.8 mmol) was added to a stirred solution of 2,4-difluoro-3-methyl-benzenecarbohydroxamic acid (460 mg, 2.46 mmol) in n-BuOH (10 mL) at room temperature. The resulting mixture was stirred at 100 °C for 4 hr. The reaction mixture was cooled to room temperature and concentrated under reduced pressure. The residue was diluted with water (10 mL), acidified with hydrochloric acid and extracted with EtOAc (20 mL × 2). The combined organic layers were washed with brine (10 mL), dried over anhydrous sodium sulfate and concentrated under reduced pressure to afford 6-fluoro-7-methyl-1,2-benzoxazol-3-one (400 mg, 1.68 mmol, 68.2% yield) as a brown solid which was

used in the next step without further purification. LCMS(ESI): [M-H]- m/z: calcd 166.03; found 166.0.

Step iii: Data for 3-ethoxy-6-fluoro-7-methyl-1,2-benzoxazole

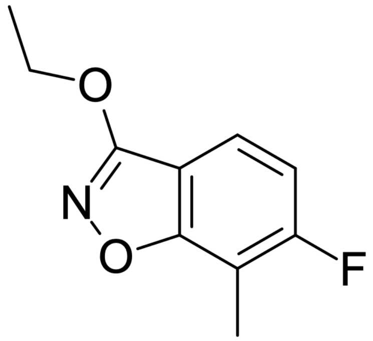

Ag_2O (776 mg, 3.35 mmol) and iodoethane (26 mg, 1.68 mmol, 135 μL) were added to a stirred solution of 6-fluoro-7-methyl-1,2-benzoxazol-3-one (400 mg, 1.68 mmol) in $CHCl_3$ (25 mL) at room temperature. The resulting mixture was stirred under reflux overnight. The reaction mixture was cooled to room temperature and filtered through a thin pad of silica gel. The filtercake was washed several times with $CHCl_3$. The filtrate was concentrated under reduced pressure. The residue was subjected to reverse phase HPLC (0-1-5 min., 35-35-80% water – ACN, flow: 30 mL/min, column: Chromatorex 18 SMB100-5T 100×19 mm, 5 μm) to afford 3-ethoxy-6-fluoro-7-methyl-1,2-benzoxazole (77.0 mg, 394 μmol, 23.6% yield) as a yellow solid. ^{1}H NMR (500 MHz, dmso) δ_H 1.51 (t, 3H), 2.39 (s, 3H), 4.47 (q, 2H), 6.98 (t, 1H), 7.39 (dd, 1H). LCMS(ESI): $[M+H]^+$ m/z: calcd 196.09; found 196.0.

Step iv: Data for 5-[3-[(3-ethoxy-7-methyl-1,2-benzoxazol-6-yl)oxy]propyl]-N,N-dimethyl-isoxazole-3-carboxamide (Compound 48)

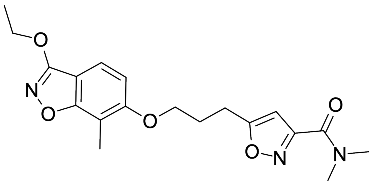

NaH (6.45 mg, 161 μmol, 60% dispersion in mineral oil) was added to a stirred solution of 5-(3-hydroxypropyl)-N,N-dimethyl-isoxazole-3-carboxamide (32.0 mg, 161 μmol) in DMF (3.0 mL) at room temperature. The mixture was stirred for 15 min then 3-ethoxy-6-fluoro-7-methyl-1,2-

benzoxazole (30.0 mg, 154 μmol) was added. The resulting mixture was stirred at 100 °C overnight. The reaction mixture was cooled to room temperature and subjected to preparative HPLC (0-6 min., 30-60% water – ACN, flow: 30 mL/min, column: Chromatorex 18 SMB100-5T 100×19 mm, 5 μm) to afford 5-[3-[(3-ethoxy-7-methyl-1,2-benzoxazol-6-yl)oxy]propyl]-N,N-dimethyl-isoxazole-3-carboxamide (5.40 mg, 14.5 μmol, 9.41% yield) as a beige solid. ^{1}H NMR (500 MHz, CD_3OD) δ_H 1.48 (t, 3H), 2.22 – 2.31 (m, 5H), 3.05 – 3.12 (m, 5H), 3.17 (s, 3H), 4.17 (t, 2H), 4.42 (q, 2H), 6.41 (s, 1H), 6.99 (d, 1H), 7.40 (d, 1H). LCMS(ESI): $[M+H]^+$ m/z: calcd 374.19; found 374.2.

Synthesis of 5-[3-[[3-(cyclopropylmethoxy)-1,2-benzoxazol-6-yl]oxy]propyl]-N,N-dimethyl-isoxazole-3-carboxamide (Compound 49)

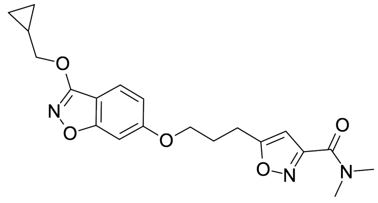

49 was synthesised by a route similar to Compound **46**: 5-(3-Hydroxypropyl)-N,N-dimethyl-isoxazole-3-carboxamide (332 mg, 1.67 mmol) was added to the previously obtained solution of 6-fluoro-3-isobutoxy-1,2-3-(cyclopropylmethoxy)-6-fluoro-1,2-benzoxazole (100 mg, 0.48 mmol) in DMF (2 mL). The obtained solution was cooled to 0 °C. To the resulting mixture NaH (19 mg, 0.48 mmol, 60% dispersion in mineral oil) was added. The reaction mixture was stirred at ambient temperature for 16 hrs. The resulting mixture was diluted with water (5 mL) and extracted with ethyl acetate (10 mL). The organic layer was washed with water (3 × 1 mL), dried over anhydrous sodium sulphate and concentrated. The residue was subjected to HPLC (0-1.3-5 min. 35-35-75%, water – ACN +0.1% vol. of 25% aq. NH_3, flow: 30 mL/min; column: XBridge C18 100×19mm,) to afford 15.1 mg of a mixture of two regioisomers. The resulting mixture was submitted to chiral HPLC (Column: CHIRALPAK IC 250×21 mm, 5 μm, mobile phase: Hexane-IPA-MeOH, 50:25:25, flow: 12 mL/min) to afford 5-[3-[[3-(cyclopropylmethoxy)-

1,2-benzoxazol-6-yl]oxy]propyl]-N,N-dimethyl-isoxazole-3-carboxamide (9.5 mg, 23.4 μmol, 4.85% yield) as a white solid. ^{1}H NMR (500 MHz, CD_3OD) δ_H 0.40 – 0.44 (m, 2H), 0.65 – 0.68 (m, 2H), 1.35 – 1.43 (m, 1H), 2.24 (p, 2H), 3.06 (t, 2H), 3.10 (s, 3H), 3.18 (s, 3H), 4.13 (t, 2H), 4.19 (d, 2H), 6.41 (s, 1H), 6.91 (dd, 1H), 6.99 (d, 1H), 7.52 (d, 1H). LCMS(ESI): $[M+H]^+$ m/z: calcd 386.18; found 386.0.

Synthesis of 5-(3-((3-isobutoxybenzo[d]isoxazol-6-yl)oxy)propyl)-N,N-dimethylisoxazole-3-carboxamide (50) & 5-(3-((6-isobutoxybenzo[d]isoxazol-3-yl)oxy)propyl)-N,N-dimethylisoxazole-3-carboxamide (51)

(Compound 50)

(Compound 51)

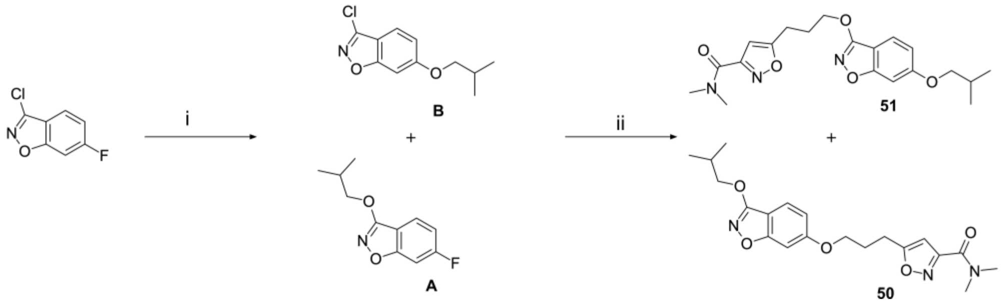

Scheme S18: Reagents and conditions: (i) NaH, EtOH, DMF, 0 °C to RT, 16h (ii) 5-(3-Hydroxypropyl)-N,N-dimethyl-isoxazole-3-carboxamide, NaH, DMF, 0 °C to RT, 16h

Compound **50** was synthesised by a route similar to Compound **46**. In this case, the isomeric product: Compound **51** was also isolated.

Step i: Data for 6-fluoro-3-isobutoxybenzo[d]isoxazole A and 3-chloro-6-isobutoxybenzo[d]isoxazole B

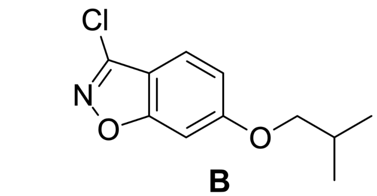

B

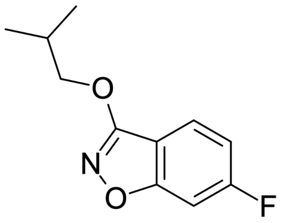

A

3-Chloro-6-fluoro-1,2-benzoxazole (400 mg, 2.33 mmol) and 2-methylpropan-1-ol (190 mg, 2.56 mmol) was dissolved in DMF (8 mL) at 0 ºC. NaH (100 mg, 2.5 mmol, 60% dispersion in mineral oil) was added portionwise at 0°C. The reaction mixture was stirred for 16 hrs. at ambient temperature. The obtained solution was used in the next step without work up. 6-fluoro-3-isobutoxy-1,2-benzoxazole (**A**): LCMS(ESI): $[M+H]^+$ m/z: calcd 210.10; found 210.0. 3-chloro-6-isobutoxybenzo[d]isoxazole (**B**): LCMS(ESI): $[M+H]^+$ m/z: calcd 226.07; found 226.0.

Step ii: Data for 5-(3-((3-isobutoxybenzo[d]isoxazol-6-yl)oxy)propyl)-N,N-dimethylisoxazole-3-carboxamide (Compound 50) and 3-chloro-6-isobutoxybenzo[d]isoxazole (Compound 51)

5-(3-Hydroxypropyl)-N,N-dimethyl-isoxazole-3-carboxamide (332 mg, 1.67 mmol) was added to the previously obtained solution of 6-fluoro-3-isobutoxy-1,2-benzoxazole (0.35 g, 1.67 mmol) in DMF (8 mL). To the resulting mixture NaH (67 mg, 1.67 mmol, 60% dispersion in mineral oil) was added at 0 ºC. The reaction mixture was stirred at ambient temperature for 16 hrs. The resulting mixture was diluted with water (10 mL) and extracted with ethyl acetate (20 mL). The organic layer was washed with water (3 × 1 mL), dried over anhydrous sodium sulphate and concentrated. The residue was subjected to HPLC (0-1.3-6 min. 35-35-75%, water - ACN, flow: 30 mL/min; column: XBridge C18 100×19mm,) to afford a mixture of two regioisomers. The resulting mixture was submitted to chiral HPLC (Column: CHIRALPAK IC 250×21 mm, 5 μm, mobile phase: IPA-MeOH, 50:50,

flow: 10 mL/min) to afford 5-[3-[(3-isobutoxy-1,2-benzoxazol-6-yl)oxy]propyl]-N,N-dimethyl-isoxazole-3-carboxamide (**50**, 8.5 mg, 21.9 μmol, 1.31% yield) and 5-(3-((6-isobutoxybenzo[d]isoxazol-3-yl)oxy)propyl)-N,N-dimethylisoxazole-3-carboxamide (**51**, 8.4 mg, 21.7 μmol, 1.30% yield).

Compound **50**

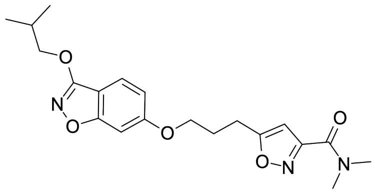

Analytical data: ^{1}H NMR (500 MHz, CD_3OD) δ_H 1.07 (d, 6H), 2.15 – 2.22 (m, 1H), 2.22 – 2.28 (m, 2H), 3.07 (t, 2H), 3.10 (s, 3H), 3.19 (s, 3H), 4.11 – 4.17 (m, 4H), 6.41 (s, 1H), 6.92 (d, 1H), 7.00 (s, 1H), 7.51 (d, 1H). LCMS(ESI): $[M+H]^+$ m/z: calcd 388.21; found 388.2. Analytical chiral HPLC (column: Chiralpak IC (250 × 4.6 mm, 5 μm; mobile phase: IPA-MeOH, 50:50; flow 0.6 mL/min.): Rt 18.37 min.

Compound **51**

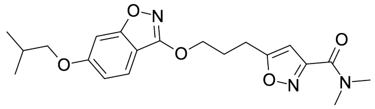

Analytical data: ^{1}H NMR (500 MHz, CD_3OD) δ_H 1.06 (d, 6H), 2.07 – 2.14 (m, 1H), 2.32 (p, 2H), 3.08 (t, 2H), 3.10 (s, 3H), 3.18 (s, 3H), 3.82 (d, 2H), 4.46 (t, 2H), 6.44 (s, 1H), 6.91 (dd, 1H), 6.97 (d, 1H), 7.49 (d, 1H). LCMS(ESI): $[M+H]^+$ m/z: calcd 388.21; found 388.2. Analytical chiral HPLC (column: Chiralpak IC (250 × 4.6 mm, 5 μm; mobile phase: IPA-MeOH, 50:50; flow 0.6 mL/min.): Rt 21.71 min.

General Procedure A: Parallel Synthesis of Phenol Ethers by Mitsunobu Coupling

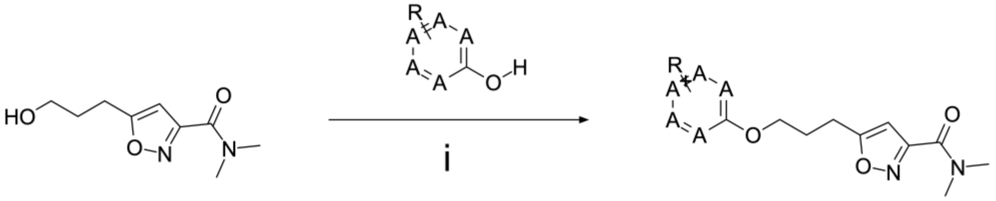

Scheme S19: Reagents and conditions: (i) hydroxy aryl compound, ADDP, PBu_3, dioxane, RT, 16h

Data for 5-(3-(3-Fluorophenoxy)propyl)-N,N-dimethylisoxazole-3-carboxamide (Compound 52)

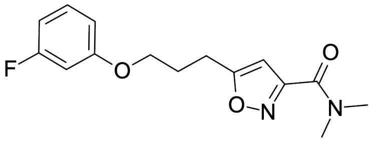

Following General Procedure A using 3-fluorophenol afforded 12.9 mg of 5-(3-(3-fluorophenoxy)propyl)-N,N-dimethylisoxazole-3-carboxamide as a beige solid (44.1 μmol, 25.8% yield). HPLC method: 1.3-7.3 min., 30-50% water – ACN, +0.1% vol. of 25% aq. NH_3, flow: 30 mL/min + 4 mL/min (organic phase), column: XBridge BEH C18 5μm 130A, 100 ×19mm, 5μm. ^{1}H NMR (500 MHz, dmso) δ_H 2.09 (p, 2H), 2.95 (t, 2H), 2.98 (s, 3H), 3.03 (s, 3H), 4.03 (t, 2H), 6.51 (s, 1H), 6.70 – 6.82 (m, 3H), 7.29 (q, 1H). LCMS(ESI): $[M+H]^+$ m/z: calcd 293.15; found 293.2.

Data for 5-(3-((1-Fluoro-5,6,7,8-tetrahydronaphthalen-2-yl)oxy)propyl)-N,N-dimethylisoxazole-3-carboxamide (Compound 53)

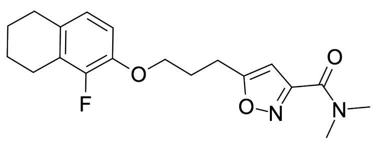

Following General Procedure A using 1-fluoro-5,6,7,8-tetrahydronaphthalen-2-ol afforded 12.0 mg of 5-(3-((1-fluoro-5,6,7,8-tetrahydronaphthalen-2-yl)oxy)propyl) -N,N-dimethylisoxazole-3-carboxamide as a white solid (34.6 μmol, 24.0% yield). HPLC method: 1.3-7.3 min., 40-65% water – ACN, +0.1% vol. of 25% aq. NH_3, flow: 30 mL/min + 4 mL/min (organic phase), column: XBridge BEH C18 5μm 130A, 100 ×19mm, 5μm. ^{1}H NMR (500 MHz, dmso) δ_H 1.62 – 1.72 (m, 4H), 2.08 (p, 2H), 2.57 – 2.67 (m, 4H), 2.94 (t, 2H), 2.97 (s, 3H), 3.03 (s, 3H), 4.02 (t, 2H), 6.50 (s, 1H), 6.79 (d, 1H), 6.88 (t, 1H). LCMS(ESI): $[M+H]^+$ m/z: calcd 347.21; found 347.2.

Data for 5-(3-(4-Fluorophenoxy)propyl)-N,N-dimethylisoxazole-3-carboxamide (Compound 54)

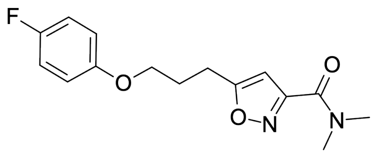

Following General Porcedure A using 4-fluorophenol afforded 15.1 mg of 5-(3-(4-fluorophenoxy)propyl)-N,N-dimethylisoxazole-3-carboxamide as a beige solid (52.0 μmol, 30.2% yield). HPLC method: 1.3-7.3 min., 30-55% water – ACN, +0.1% vol. of 25% aq. NH_3, flow: 30 mL/min + 4 mL/min (organic phase), column: XBridge BEH C18 5μm 130A, 100 ×19mm, 5μm. ^{1}H NMR (500 MHz, dmso) δ_H 2.08 (p, 2H), 2.95 (t, 2H), 2.98 (s, 3H), 3.03 (s, 3H), 3.98 (t, 2H), 6.50 (s, 1H), 6.88 – 6.96 (m, 2H), 7.09 (t, 2H). LCMS(ESI): $[M+H]^+$ m/z: calcd 293.15; found 293.2.

Data for 5-(3-(4-(prop-2-yn-1-yl)phenoxy)propyl)-N,N-Dimethylisoxazole-3-carboxamide (Compound 55)

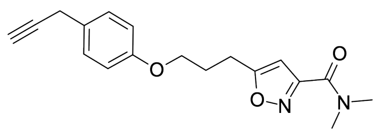

Following General Procedure A using 4-(prop-2-yn-1-yl)phenol afforded 19.5 mg of 5-(3-(4-(prop-2-yn-1-yl)phenoxy)propyl)-N,N-Dimethylisoxazole-3-carboxamide as a yellow solid (62.4 μmol, 39.0% yield). HPLC method: 1.3-7.3 min., 30-50% water – ACN, +0.1% vol. of 25% aq. NH_3, flow: 30 mL/min + 4 mL/min (organic phase), column: XBridge BEH C18 5μm 130A, 100 ×19mm, 5μm. ^{1}H NMR (500 MHz, dmso) δ_H 2.09 (p, 2H), 2.95 (t, 2H), 2.97 (s, 3H), 3.00 (s, 1H), 3.03 (s, 3H), 3.52 (s, 2H), 3.99 (t, 2H), 6.50 (s, 1H), 6.87 (d, 2H), 7.20 (d, 2H). LCMS(ESI): $[M+H]^+$ m/z: calcd 313.18; found 313.2.

Data for 5-(3-(4-(But-3-yn-1-yl)phenoxy)propyl)-N,N-dimethylisoxazole-3-carboxamide (Compound 56)

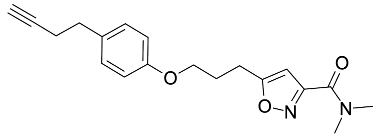

Following General Procedure A using 4-(But-3-yn-1-yl)phenol afforded 14.1 mg of 5-(3-(4-(but-3-yn-1-yl)phenoxy)propyl)-N,N-dimethylisoxazole-3-carboxamide as a yellow gum (43.2 μmol, 28.2% yield). HPLC method: 1.3-7.3 min., 30-50% water – ACN, +0.1% vol. of 25% aq. NH_3, flow: 30 mL/min + 4 mL/min (organic phase), column: XBridge BEH C18 5μm 130A, 100 ×19mm, 5μm. ^{1}H NMR (500 MHz, dmso) δ_H 2.08 (p, 2H), 2.35 – 2.40 (m, 2H), 2.66 (t, 2H), 2.74 (t, 1H), 2.94 (t, 2H), 2.97 (s, 3H), 3.03 (s, 3H), 3.98 (t, 2H), 6.50 (s, 1H), 6.82 (d, 2H), 7.13 (d, 2H). LCMS(ESI): $[M+H]^+$ m/z: calcd 327.30; found 327.2.

Synthesis of (E)-N-ethoxy-1-[4-[2-[1-(6-methylpyridazin-3-yl)-4-piperidyl] ethoxy]phenyl]methanimine (Compound S-57 (BTA-188))

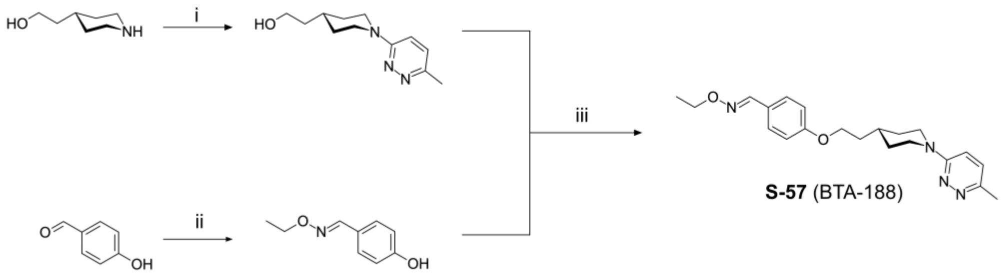

Scheme S20: Reagents and conditions: (i) 3-chloro-6-methyl-pyridazine, Na_2CO_3, DMAA, 150 °C, 16h (ii) O-ethylhydroxylamine, $NaHCO_3$, dioxane, reflux, 16h (iii) PPh_3, DIAD, THF, 16h

Step i: Data for 2-[1-(6-methylpyridazin-3-yl)-4-piperidyl]ethanol

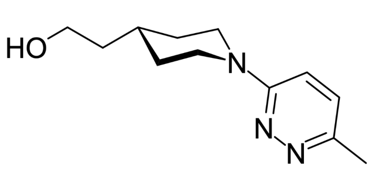

Na_2CO_3 (1.28 g, 12.1 mmol) and 2-(4-piperidyl)ethanol (1.00 g, 6.04 mmol, HCl) were added to a stirred solution of 3-chloro-6-methyl-pyridazine (776 mg, 6.04 mmol) in DMAA (5.0 mL) at room temperature. The resulting mixture was stirred at 150 °C overnight. The resulting mixture was cooled to room temperature and filtered through a thin pad of silica. The filtrate was subjected to HPLC (0-1-5 min., 10-10-50% water – MeOH, +0.1% vol. of 25% aq. NH_3, flow: 30 mL/min, column: XBridge C18 100×19 mm, 5 μm) to afford 2-[1-(6-methylpyridazin-3-yl)-4-piperidyl]ethanol (570 mg, 2.58 mmol, 42.7% yield) as a brown solid. LCMS(ESI): $[M+H]^+$ m/z: calcd 222.19; found 222.2.

Step ii: Data for 4-[(E)-ethoxyiminomethyl]phenol

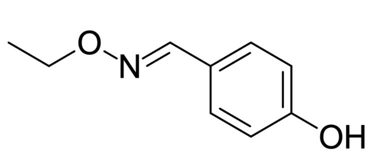

O-ethylhydroxylamine (3.99 g, 40.9 mmol, HCl) and $NaHCO_3$ (3.44 g, 40.9 mmol) were added to a stirred solution of 4-hydroxybenzaldehyde (1.00 g, 8.19 mmol, 886 μL) in

dioxane (20 mL) at room temperature. The resulting mixture was stirred under reflux overnight. The reaction mixture was cooled to room temperature and concentrated under reduced pressure. The residue was diluted in EtOAc (50 mL) and washed with water (10 mL). The organic layer was separated, dried over anhydrous sodium sulfate and concentrated under reduced pressure to afford 4-[(E)-ethoxyiminomethyl]phenol (1.30 g, 7.87 mmol, 96.1% yield) as a beige solid which was used in the next step without further purification. LCMS(ESI): $[M+H]^+$ m/z: calcd 166.09; found 166.2.

Step iii: Data for (E)-N-ethoxy-1-[4-[2-[1-(6-methylpyridazin-3-yl)-4-piperidyl]ethoxy]phenyl]methanimine (Compound S-57 (BTA-188))

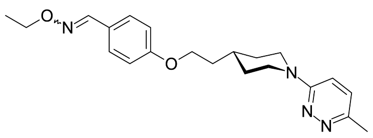

2-[1-(6-Methylpyridazin-3-yl)-4-piperidyl]ethanol (100 mg, 452 μmol), 4-[(E)-ethoxyiminomethyl]phenol (82.1 mg, 497 μmol) and PPh_3 (130 mg, 497 μmol) were dissolved in THF (5.0 mL) at room temperature. To the obtained solution DIAD (101 mg, 497 μmol, 98 μL) was added dropwise at room temperature. The resulting mixture was stirred at room temperature overnight. The reaction mixture was concentrated under reduced pressure. The residue was subjected to HPLC (0-1-5 min., 45-45-65% water – ACN, +0.1% vol. of 25% aq. NH_3, flow: 30 mL/min, column: XBridge C18 100×19 mm, 5 μm) to afford (E)-N-ethoxy-1-[4-[2-[1-(6-methylpyridazin-3-yl)-4-piperidyl]ethoxy]phenyl]methanimine (32.0 mg, 86.9 μmol, 19.2% yield) as a beige solid. ^{1}H NMR (500 MHz, dmso) δ_H 1.16 – 1.26 (m, 5H), 1.66 – 1.72 (m, 2H), 1.73 – 1.82 (m, 3H), 2.41 (s, 3H), 2.84 (t, 2H), 4.07 (t, 2H), 4.11 (q, 2H), 4.29 (d, 2H), 6.98 (d, 2H), 7.21 (q, 2H), 7.53 (d, 2H), 8.14 (s, 1H). LCMS(ESI): $[M+H]^+$ m/z: calcd 369.27; found 369.2. The data were in agreement with those published.[S1]

Synthesis of 1-(2-amino-4-pyridyl)-3-[5-[4-(5-methyl-1,2,4-oxadiazol-3-yl)phenoxy]pentyl]imidazolidin-2-one (Compound S-59 (NLD-22))

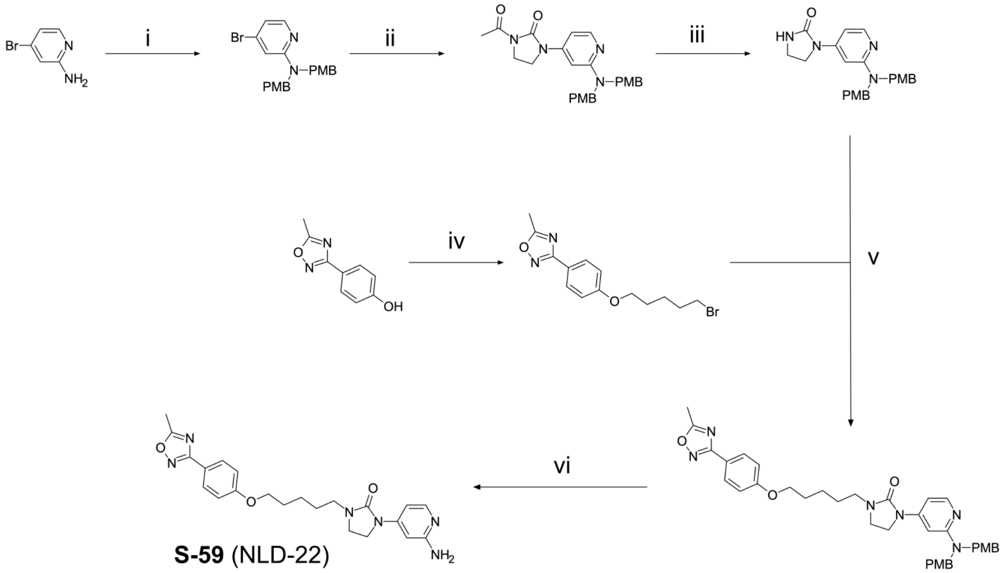

Scheme S21: Reagents and conditions: (i) 1-(chloromethyl)-4-methoxy-benzene, NaH, DMF, 0 °C to RT, 16h (ii) 1-acetylimidazolidin-2-one, Cs_2CO_3, $Pd(dba)_2$, Xantphos, toluene, 100 °C, 16h (iii) K_2CO_3, MeOH, RT, 16h (iv) 5-bromopentan-1-ol, PPh_3, DIAD, THF, 0 °C to RT, 16h (v) NaH, DMF, 0 °C to RT, 16h (vi) TFA, DCM, 0 °C to RT, 16h

Step i: Data for 4-bromo-N,N-bis[(4-methoxyphenyl)methyl]pyridin-2-amine

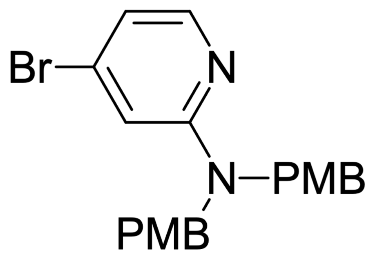

4-Bromopyridin-2-amine (1.00 g, 5.78 mmol) was added to a stirred solution of NaH (925 mg, 23.1 mmol, 60% dispersion in mineral oil) in DMF (20 mL) at 0 °C. The resulting mixture was stirred at ambient temperature for 30 min. The mixture was cooled to 0 °C then 1-(chloromethyl)-4-methoxy-benzene (2.26 g, 14.5 mmol) was added. The resulting mixture was stirred at room temperature for 16 hr. The reaction mixture was quenched by addition of

water (25 mL). The resulting mixture was extracted with EtOAC (3×15 mL). The combined organic layers were washed with water (3×15 mL) and brine (15 mL), dried over anhydrous sodium sulfate and concentrated under reduced pressure to afford 4-bromo-N,N-bis[(4-methoxyphenyl)methyl]pyridin-2-amine (1.70 g, 4.11 mmol, 71.2% yield) as a yellow solid which was used in the next step without further purification. LCMS(ESI): $[M+H]^+$ m/z: calcd 413.09; found 413.0.

Step ii: Data for 1-acetyl-3-[2-[bis[(4-methoxyphenyl)methyl]amino]-4-pyridyl]imidazolidin-2-one

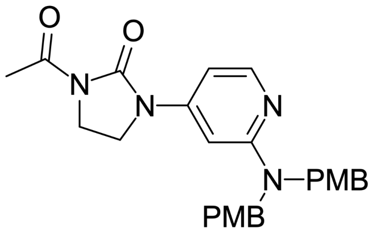

Cs_2CO_3 (591 mg, 1.81 mmol), 4-bromo-N,N-bis [(4-methoxyphenyl)methyl]pyridin-2-amine (500 mg, 1.21 mmol) and 1-acetylimidazolidin-2-one (465 mg, 3.63 mmol) were mixed in toluene (20 mL). The resulting suspension was degassed and backfilled with argon. To the resulting mixture $Pd(dba)_2$ (69.6 mg, 121 μmol) and Xantphos (70.00 mg, 121 μmol) were added sequentially. The reaction mixture was stirred at 100 °C for 16 hr. The reaction mixture was cooled to room temperature and filtered. The filtrate was concentrated under reduced pressure. The residue was subjected to column chromatography (SiO_2, eluent DCM - MeOH in ratio 95:5) to afford 1-acetyl-3-[2-[bis[(4-methoxyphenyl)methyl]amino]-4-pyridyl]imidazolidin-2-one (200 mg, 434 μmol, 35.9% yield) as a yellow solid. LCMS(ESI): $[M+H]^+$ m/z: calcd 461.22; found 461.2.

Step iii: Data for 1-[2-[bis[(4-methoxyphenyl)methyl]amino]-4-pyridyl] imidazolidin-2-one

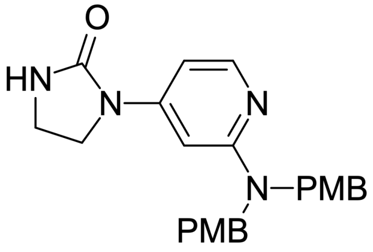

K_2CO_3 (180 mg, 1.30 mmol) was added to a stirred solution of 1-acetyl-3-[2-[bis[(4-methoxyphenyl)methyl]amino]-4-pyridyl]imidazolidin-2-one (200 mg, 434 μmol) in MeOH (15 mL) at room temperature. The resulting mixture was stirred at room temperature for 16 hr. The reaction mixture was diluted in water (30 mL) and filtered. The precipitate was washed with water and dried to afford 1-[2-[bis[(4-methoxyphenyl)methyl]amino]-4-pyridyl]imidazolidin-2-one (180 mg, 430 μmol, 99.0% yield) as a white solid which was used in the next step without further purification. LCMS(ESI): $[M+H]^+$ m/z: calcd 419.21; found 419.2

Step iv: Data for 3-[4-(5-bromopentoxy)phenyl]-5-methyl-1,2,4-oxadiazole

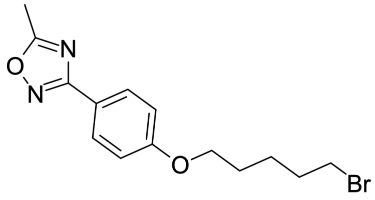

4-(5-Methyl-1,2,4-oxadiazol-3-yl)phenol (0.5 g, 2.84 mmol), 5-bromopentan-1-ol (545 mg, 3.26 mmol) and PPh_3 (856 mg, 3.26 mmol) were mixed in THF (20 mL) at room temperature. The mixture was cooled to 0 °C. DIAD (660 mg, 3.26 mmol, 643 μL) was added to the obtained mixture. The resulting reaction mixture was stirred under ambient conditions for 16 hr. The reaction mixture was concentrated under reduced pressure. The obtained residue was subjected to flash column chromatography (SiO_2, eluent Hex - EOAc in ratio 8:2) to afford 3-[4-(5-bromopentoxy)phenyl]-5-methyl-1,2,4-oxadiazole (0.60 g, 1.85 mmol, 65.0% yield) as a yellow gum. LCMS(ESI): $[M+H]^+$ m/z: calcd 325.06; found 325.0.

Step v: Data for 1-[2-[bis[(4-methoxyphenyl)methyl]amino]-4-pyridyl]-3-[5-[4-(5-methyl-1,2,4-oxadiazol-3-yl)phenoxy]pentyl]imidazolidin-2-one

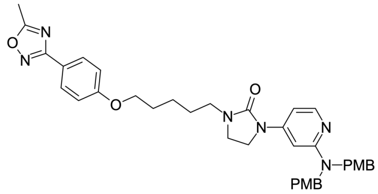

1-[2-[Bis[(4-methoxyphenyl)methyl]amino]-4-pyridyl]imidazolidin-2-one (90.0 mg, 215 μmol) was added to a stirred solution of NaH (12.9 mg, 323 μmol, 60% dispersion in oil) in DMF (10 mL) at 0 °C. The resulting mixture was stirred at ambient temperature for 30 min. The mixture was cooled to 0 °C then 3-[4-(5-bromopentoxy)phenyl]-5-methyl-1,2,4-oxadiazole (70.0 mg, 215 μmol) was added to the mixture. The reaction mixture was stirred at room temperature for 16 hr. The reaction mixture was diluted with water (20 mL) and extracted with EtOAc (2×15 mL). The combined organic layers were washed with water (2×10 mL) and brine (10 mL), dried over anhydrous sodium sulfate and concentrated under reduced pressure to afford 1-[2-[bis[(4-methoxyphenyl)methyl]amino]-4-pyridyl]-3-[5-[4-(5-methyl-1,2,4-oxadiazol-3-yl)phenoxy]pentyl]imidazolidin-2-one (100 mg, 151 μmol, 70.2% yield) as a yellow gum which was used in the next step without further purification. LCMS(ESI): $[M+H]^+$ m/z: calcd 663.33; found 663.2.

Step vi: Data for -(2-amino-4-pyridyl)-3-[5-[4-(5-methyl-1,2,4-oxadiazol-3-yl)phenoxy]pentyl]imidazolidin-2-one (Compound S-59 (NLD-22))

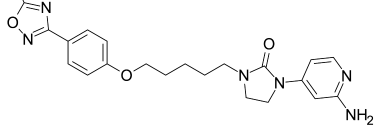

1-[2-[Bis[(4-methoxyphenyl)methyl]amino]-4-pyridyl]-3-[5-[4-(5-methyl-1,2,4-oxadiazol-3-yl)phenoxy]pentyl]imidazolidin-2-one (200 mg, 302 μmol) was dissolved in DCM (10 mL) and the resulting solution was cooled to 0 °C. TFA (344 mg, 3.02 mmol, 231 μL) was added to the stirred solution. The resulting mixture was stirred at room

temperature for 16 hr. The reaction mixture was concentrated under reduced pressure. The residue was subjected to HPLC (0-1-5 min., 40-40-80% water – MeOH, +0.1% vol. of 25% aq. NH_3, flow: 30 mL/min, column: XBridge C18 100×19 mm, 5 μm) to afford 1-(2-amino-4-pyridyl)-3-[5-[4-(5-methyl-1,2,4-oxadiazol-3-yl)phenoxy]pentyl]imidazolidin-2-one (26.0 mg, 61.5 μmol, 20.4% yield) as a white solid. ^{1}H NMR (500 MHz, dmso) δ_H 1.39 – 1.46 (m, 2H), 1.53 – 1.61 (m, 2H), 1.74 – 1.81 (m, 2H), 2.63 (s, 3H), 3.21 (t, 2H), 3.42 – 3.48 (m, 2H), 3.68 – 3.73 (m, 2H), 4.05 (t, 2H), 5.71 (s, 2H), 6.55 (d, 1H), 6.81 (dd, 1H), 7.09 (d, 2H), 7.24 (d, 1H), 7.91 (d, 2H). LCMS(ESI): $[M+H]^+$ m/z: calcd 423.24; found 423.2. The data were in agreement with those published.[S2]

Synthesis of 6-phenyl-N3-[4-(trifluoromethyl)phenyl]-1H-pyrazolo[3,4-d]pyrimidine-3,4-diamine (Compound S-60 (OBR-5-340))

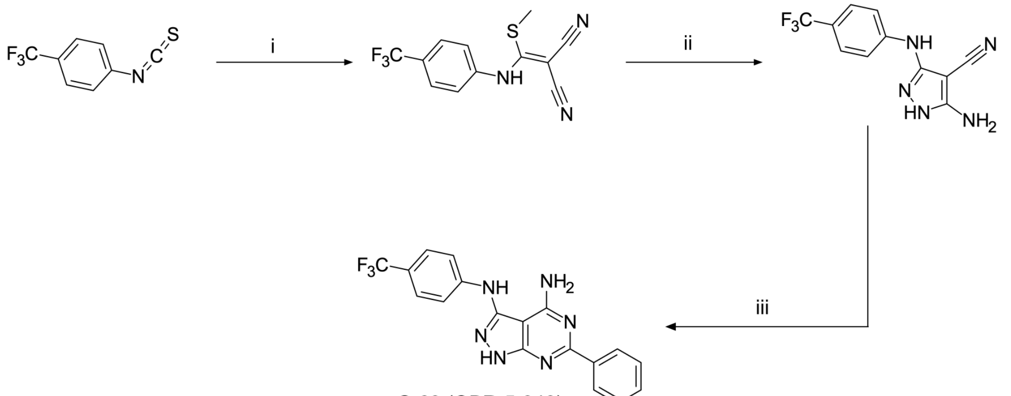

S-60 (OBR-5-340)

Scheme S22: Reagents and conditions: (i) methyl iodide, malonitrile, NaH, DMF, RT, 16h (ii) hydrazine hydrate, EtOH, RT, reflux, 3h (iii) benzamidine, NaOAc, neat, 180 °C, 30 mins

Step i: Data for 2-[methylsulfanyl-[4-(trifluoromethyl)anilino]methylene] propanedinitrile

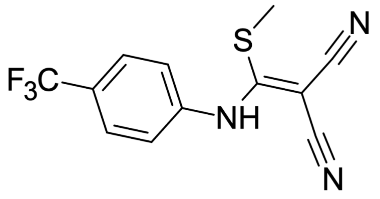

NaH (594 mg, 14.8 mmol, 60% dispersion in mineral oil) was added portionwise to a stirred solution of propanedinitrile (943 mg, 14.3 mmol) in DMF (50 mL) at room temperature. The resulting mixture was stirred at ambient temperature for 30 min. A solution of 1-isothiocyanato-4-(trifluoromethyl)benzene (2.90 g, 14.3 mmol) in DMF (5.0 mL) was added dropwise to the mixture. The resulting mixture was stirred at ambient temperature for 1 hr. Iodomethane (2.03 g, 14.3 mmol, 889 μL) was added dropwise at room temperature to the mixture. The resulting mixture was stirred at room temperature overnight. The reaction mixture was poured into water (400 mL), the obtained precipitate was filtered, washed with water (2×50 mL) and air dried to afford 2-[methylsulfanyl-[4-(trifluoromethyl)anilino]methylene]propanedinitrile (3.60 g, 10.9 mmol, 76.6% yield) as a yellow solid which was used in the next step without further purification. LCMS(ESI): $[M+H]^+$ m/z: calcd 284.05; found 284.0.

Step ii: Data for 6-phenyl-N3-[4-(trifluoromethyl)phenyl]-1H-pyrazolo[3,4-d]pyrimidine-3,4-diamine

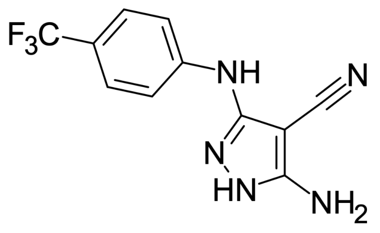

Hydrazine hydrate (3.50 g, 69.9 mmol) was added to a stirred solution of 2-[methylsulfanyl-[4-(trifluoromethyl) anilino]methylene]propanedinitrile (3.60 g, 12.7 mmol) in EtOH (50 mL) at room temperature. The resulting mixture was stirred under reflux for 3 hr. The reaction mixture was cooled to room temperature and diluted with water (50 mL). The obtained precipitate was filtered, washed with water (2×10 mL) and air dried to afford 3-amino-5-[4-(trifluoromethyl)anilino]-1H-pyrazole-4-carbonitrile (2.40 g, 8.98 mmol,

70.7% yield) as a beige solid which was used in the next step without further purification. LCMS(ESI): $[M+H]^+$ m/z: calcd 268.08; found 268.0.

Step iii: Data for 3-amino-5-[4-(trifluoromethyl)anilino]-1H-pyrazole-4-carbonitrile

(Compound S-60 (OBR-5-340))

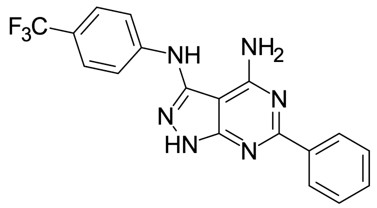

A mixture of 5-amino-3-[4-(trifluoromethyl)anilino]-1H-pyrazole-4-carbonitrile (800 mg, 2.99 mmol), benzamidine (1.17 g, 7.48 mmol, HCl) and NaOAc (614 mg, 7.48 mmol) was heated in neat at 180 °C for 30 min. The reaction mixture was cooled to room temperature and subjected to HPLC (0-1-5 min., 65-65-90% water – MeOH, +0.1% vol. of 25% aq. NH_3, flow: 30 mL/min, column: XBridge C18 100×19 mm, 5 μm) to afford 6-phenyl-N3-[4-(trifluoromethyl)phenyl]-1H-pyrazolo[3,4-d]pyrimidine-3,4-diamine (60.0 mg, 162 μmol, 5.41% yield) as a white solid. ^{1}H NMR (500 MHz, dmso) δ_H 7.45 – 7.51 (m, 3H), 7.56 (br., s, 2H), 7.61 (d, 2H), 7.80 (d, 2H), 8.34 – 8.40 (m, 2H), 8.86 (s, 1H), 12.75 (br., s, 1H). LCMS(ESI): $[M+H]^+$ m/z: calcd 371.14; found 371.2.The data were in agreement with those published.[S3]

Methodology for Antiviral and ADME Assays

EV-D68 Antiviral Screening Assay in RD Cells

For the full protocol, please visit the following entry on protocols.io:

DOI dx.doi.org/10.17504/protocols.io.yxmvmywybv3p/v1

RD cells (2k per well) were seeded in 96 Well Black/Clear Bottom Plates and incubated overnight. On the following day, cell density was determined using Trypan Blue. Infection media containing DMSO was prepared, and deep 96-well plates were filled with 800 μL per well, except for column 12. Diluted compounds were added to the designated wells. After a 2.5-hour incubation at 37°C with 5% CO2, the test plate was infected with EV-D68 (strain US/MO/14-18949, ATCC/BEI Resources) at an MOI of 1. After 24 hours of infection, media was aspirated, and cells were fixed with 4% Formaldehyde + PBS.

Following fixation, formaldehyde was carefully removed and the monolayers were washed once with PBS. PBS was discarded, and cells were incubated with 0.1% Triton X-100 for 20 minutes. The monolayer was washed three times with PBS. Cells were then incubated with 0.3% BSA/PBS for 30 minutes. The primary antibody, Anti-Enterovirus D68 VP1 (GTX132313), was diluted in 0.3% BSA/PBS and added to the wells. Following a 1-hour incubation, cells were washed three times with PBS. The secondary antibody, goat anti-rabbit IgG (H+L) Alexa Fluor 488 (ab150077), diluted in 0.3% BSA/PBS, was added to the cells and incubated for an additional hour, protected from light. The monolayer was washed three times with PBS and counterstained with DAPI. The final PBS wash was left on the cells for imaging.

Imaging was performed using the Cytation 1 system, Viral infection and cell viability were assessed using fluorescence imaging to quantify Alexa Fluor 488 and DAPI signals. Images were analyzed using Gen5 software to calculate infection rates and determine antiviral efficacy.

Rupintrivir was used as a positive antiviral control. RD cell cytotoxicity was determined by MTT assays on uninfected cells.

EV Panel Antiviral Screening Assays

For the full protocol, please visit the following entry on protocols.io:

DOI dx.doi.org/10.17504/protocols.io.5qpvo9jddv4o/v1

Cell monolayers were prepared in 96-well plates and exposed to eight serial half-log10 concentrations of test compounds, along with infected and uninfected controls, and a known active drug. After virus inoculation and incubation until >80% CPE was observed in virus controls, cell viability was quantified using neutral red staining and spectrophotometric analysis at 540 nm.

ADME Assays - LogD

For the full protocol, please visit the following entry on protocols.io:

DOI dx.doi.org/10.17504/protocols.io.e6nvw14kdlmk/v1

LogD was measured at pH 7.4 using the shake-flask method, optimised for high-throughput experimentation.[S4]

ADME Assays - KSOL

For the full protocol, please visit the following entry on protocols.io:

DOI dx.doi.org/10.17504/protocols.io.j8nlk8y41l5r/v1

Kinetic aqueous solubility was measured using the shake-flask method.

ADME Assays - MDCK Permeability

For the full protocol, please visit the following entry on protocols.io:

DOI dx.doi.org/10.17504/protocols.io.n2bvjne6ngk5/v1

Membrane permeability was measured by assessing the flux of compound across monolayers of Madine Darby Canine Kidney II (MDCKII) cells transfected with the human Multidrug Resistance 1 gene (MDR1), expressing the efflux transporter protein: P-gp.[S5–S11] For broader screening, the rate of apical to basolateral (A→B) permeability was determined. In some cases, the B→A direction was measured, in order to obtain the efflux ratio (ER = B→A / A→B).

ADME Assays - Human and Mouse Microsomal Stability

For the full protocol, please visit the following entry on protocols.io:

DOI dx.doi.org/10.17504/protocols.io.5qpvokdb9l4o/v1

Test compounds in 96-well plate format were assessed for their susceptibility to metabolism by liver enzymes, primarily cytochrome P450 (CYP) enzymes. Using NADPH supplemented human liver microsomes (HLM) or mouse liver microsomes (MLM).

Microsomal incubations are carried out in 5 aliquots of 30 μL (one for each time point). The liver microsomal incubation medium comprises phosphate buffer (100 mM, pH 7.4), $MgCl_2$ (3.3 mM), NADPH (3 mM), glucose-6-phosphate (5.3 mM), glucose-6-phosphate dehydrogenase (0.67 units/mL) with 0.415 mg of liver microsomal protein per mL. In the negative control reactions, the NADPH-cofactor system is substituted with phosphate buffer. Test compounds (2 μM, final acetonitrile concentration 1.6%) are incubated with microsomes at 37°C, shaking at 100 rpm. Five time points over 40 minutes are analysed (0, 7, 15, 25, and 40 min). The reactions are stopped by adding 5 volumes of acetonitrile with internal standard to incubation aliquots, followed by protein sedimentation by centrifuging at 5500

rpm for 5 minutes. Supernatants are analyzed using the HPLC-MS/MS system coupled with a tandem mass spectrometer. The elimination constant (k_{el}), half-life ($t_{1/2}$), and intrinsic clearance (Cl_{int}) are determined in a plot of ln(AUC) versus time, using linear regression analysis:

$$k_{\mathrm{el}} = -slope \tag{1}$$

$$t_{1/2} = \frac{\ln 2}{k_{\mathrm{el}}} \tag{2}$$

$$Cl_{\mathrm{int}} = \frac{\ln 2}{t_{1/2}} * \frac{Incubation\, Volume(\mu L)}{Protein\, In\, Incubation(mg)} \tag{3}$$

Antiviral and ADME profiling of Published Capsid-Targeting EV-D68 Antivirals

Table S1: Measured EV-D68 antiviral activity (RD cells) and and *in-vitro* ADME profile of published enterovirus capsid-interacting antiviral compounds. EC_{50} and CC_{50} values are stated as the geometric mean.

Identifier	Synonym	Ref	Structure	EC_{50}[CC_{50}] (μM)	LogD (pH 7.4)	KSOL (μM)	MDCKII-MDR1 P_{app}A→B (10^{-6} cm s^{-1})	Cl_{int} HLM , MLM (μL min^{-1} mg^{-1})
**1**	Pleconaril	S12–S14	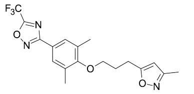	<0.0295[>50]	4.2	18	1.4	12 , 22
**2**	CP-11526092	S15,S16	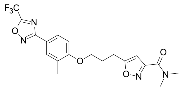	0.043[>42]	≥4.5	9	2.3	11 , 24
**3**	Vapendavir	S17–S20	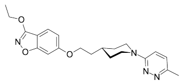	0.170[>50]	3	16	2.4	42 , 95
**S-57**	BTA-188	S1	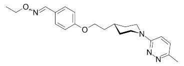	>2.24[>50]	4.4	8	7.2	18 , 38
**S-58**	Pirodavir	S21	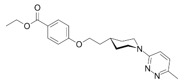	0.277[>50]	4.1	9	9	708 , 580
**S-59**	NLD-22	S2	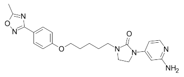	<5.57[>25.6]	2.5	10	1.9	33 , 30
**S-60**	OBR-5-340	S3	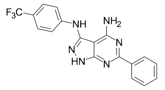	7.16[>50]	≥4.5	2	1.8	<10 , 11

Published EV Capsid Structural Data

List of Ligand-Bound Crystal and Cryo-EM Structures of VP1

Table S2: List of known published enterovirus capsid structures with a small molecule ligand bound to VP1. For further structural work relating to numerous WIN compounds and how they bind to the HRV-14 capsid VP1, see the seminal work of Rossmann et al.[S22] XRD = X-Ray Diffraction, CryoEM = Cryo Electron Microscopy, EV = Enterovirus, POV = Poliovirus, HRV = Human Rhinovirus, Cox = Coxsackievirus.

Compound	Method	PDB ID	Organism	Resolution (Å)
	XRD	1C8M	HRV16	2.80
	XRD	1NA1	HRV14	3.30
	XRD	1NCQ	HRV14	2.50
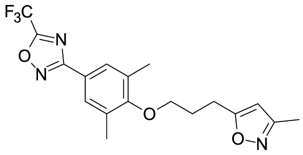	XRD	1NCR	HRV16	2.70
	XRD	1ND3	HRV16	2.80
**1** (pleconaril)	XRD	4WM7	EV-D68	2.32
	CryoEM	7OZK	EV-70	2.31
	CryoEM	7TAG	EV-D68	2.70
	CryoEM	8AYY	POV-3 (VLP)	2.60
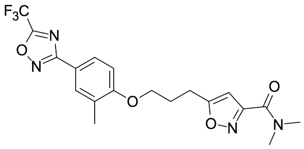 **2** (CP-11526092))	CryoEM	7TAF	EV-D68	2.00

Table S2: (Continued)

Compound	Method	PDB ID	Organism	Resolution (Å)
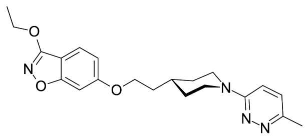 **3** (Vapendavir)	XRD	3VDD	HRV2	3.20
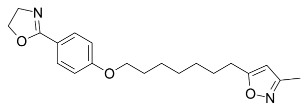 **S-61 (Disoxaril)**	XRD	1D4M	CoxA9	2.9
	XRD	1PIV	POV-3	2.9
	XRD	2R04	HRV-B14	3.0
	XRD	3ZFF	EV-A71	4.40
	XRD	3ZFG	EV-A71	3.2
	CryoEM	7OZL	EV-70	2.74
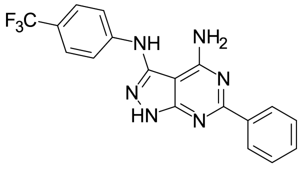 **S60** (OBR-5-340)	CryoEM	6SK5	HRV-B5	3.60
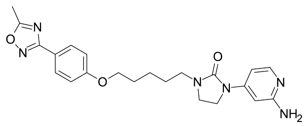 **S-59** (NLD-22)	CryoEM	6LQD	EV-A71	3.26

Table S2: (Continued)

Compound	Method	PDB ID	Organism	Resolution (Å)
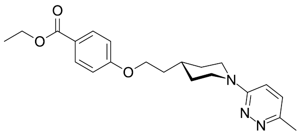 **S-58** (Pirodavir)	XRD	1PO2	POV-1	2.9
	XRD	1VBC	POV-3	2.8
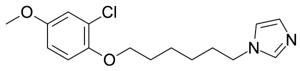 **S-62** (SCH 38057)	XRD	1HRI	HRV-B14	3.00

3D Overlays

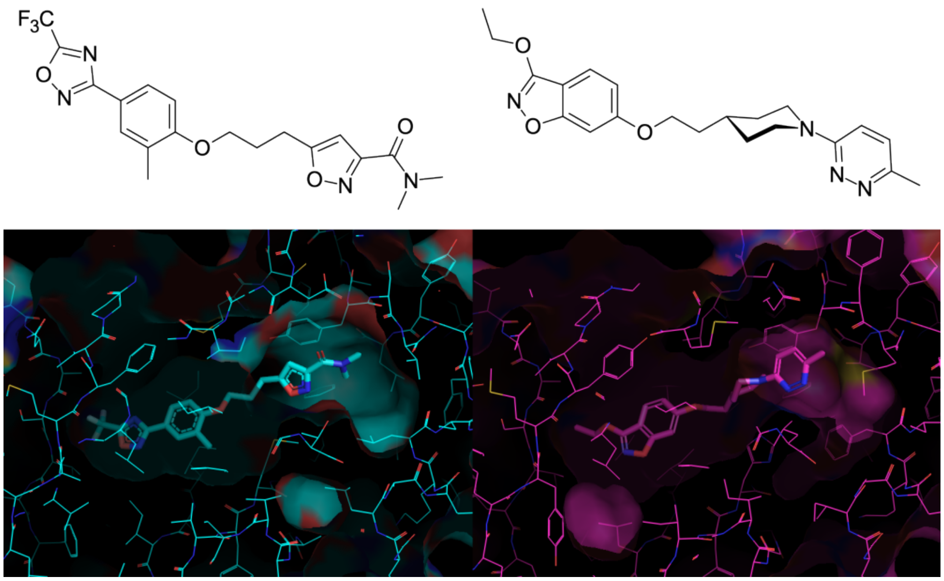

Figure S1: Aligned protein structures of EV-D68 (cyan, 7TAF)[S16] and HRV-A2 (magenta, 3VDD)[S20] capsid VP1 bound to CP-11526092 and vapendavir, respectively.

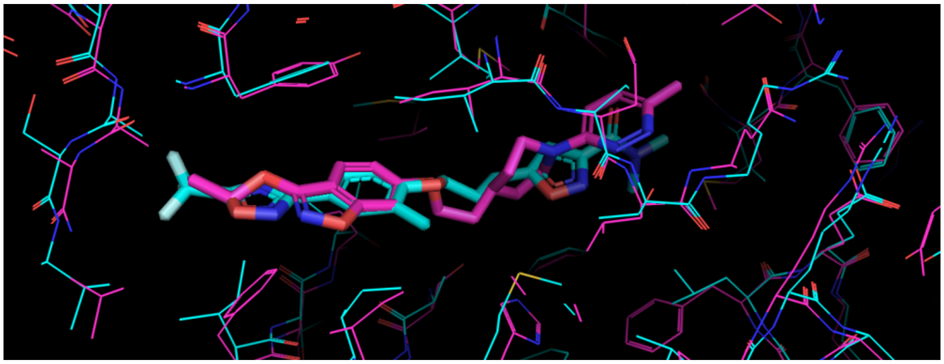

Figure S2: Overlay of 7TAF (cyan) and 3VDD (magenta), showing the relative orientation of the bound ligand with respect to the capsid VP1 hydrophobic pocket. Analysis such as this guided the design of vapendavir/pleconaril hybrid compounds seen in Table 5 of the main text.

References

References

(S1) Watson, K. G.; Brown, R. N.; Cameron, R.; Chalmers, D. K.; Hamilton, S.; Jin, B.; Krippner, G. Y.; Luttick, A.; McConnell, D. B.; Reece, P. A.; Ryan, J.; Stanislawski, P. C.; Tucker, S. P.; Wu, W.-Y.; Barnard, D. L.; Sidwell, R. W. An Orally Bioavailable Oxime Ether Capsid Binder with Potent Activity against Human Rhinovirus. *Journal of Medicinal Chemistry* **2003**, *46*, 3181–3184.

(S2) Zhang, M.; Wang, Y.; He, W.; Sun, Y.; Guo, Y.; Zhong, W.; Gao, Q.; Liao, M.; Wang, X.; Cai, Y.; Guo, Y.; Rao, Z. Design, Synthesis, and Evaluation of Novel Enterovirus 71 Inhibitors as Therapeutic Drug Leads for the Treatment of Human Hand, Foot, and Mouth Disease. *Journal of Medicinal Chemistry* **2020**, *63*, 1233–1244.

(S3) Makarov, V. A.; Braun, H.; Richter, M.; Riabova, O. B.; Kirchmair, J.; Kazakova, E. S.; Seidel, N.; Wutzler, P.; Schmidtke, M. Pyrazolopyrimidines: Potent Inhibitors Targeting the Capsid of Rhino- and Enteroviruses. *ChemMedChem* **2015**, *10*, 1629–1634.

(S4) Wilson, D.; Wang, X.; Walsh, E.; Rourick, R. High Throughput Log D Determination Using Liquid Chromatography-Mass Spectrometry. *Combinatorial Chemistry & High Throughput Screening* **2001**, *4*, 511–519.

(S5) Furubayashi, T.; Inoue, D.; Nishiyama, N.; Tanaka, A.; Yutani, R.; Kimura, S.; Katsumi, H.; Yamamoto, A.; Sakane, T. Comparison of Various Cell Lines and Three-Dimensional Mucociliary Tissue Model Systems to Estimate Drug Permeability Using an In Vitro Transport Study to Predict Nasal Drug Absorption in Rats. *Pharmaceutics* **2020**, *12*, 79.

(S6) Kuteykin-Teplyakov, K.; Luna-Tortós, C.; Ambroziak, K.; Löscher, W. Differences in the Expression of Endogenous Efflux Transporters in*MDR1*-transfected versus Wild-type Cell Lines Affect P-glycoprotein Mediated Drug Transport. *British Journal of Pharmacology* **2010**, *160*, 1453–1463.

(S7) Srinivasan, B.; Kolli, A. R.; Esch, M. B.; Abaci, H. E.; Shuler, M. L.; Hickman, J. J. TEER Measurement Techniques for In Vitro Barrier Model Systems. *SLAS Technology* **2015**, *20*, 107–126.

(S8) Taub, M. E.; Podila, L.; Ely, D.; Almeida, I. FUNCTIONAL ASSESSMENT OF MULTIPLE P-GLYCOPROTEIN (P-GP) PROBE SUBSTRATES: INFLUENCE OF CELL LINE AND MODULATOR CONCENTRATION ON P-GP ACTIVITY. *Drug Metabolism and Disposition* **2005**, *33*, 1679–1687.

(S9) Xiong, X.-H.; Huang, L.-H.; Zhong, Y.-M.; Cheng, X.-G.; Cen, M.-F.; Wang, G.-X.; Zang, L.-Q.; Wang, S.-J. Absorption Mechanism of Oxymatrine in Cultured Madin–Darby Canine Kidney Cell Monolayers. *Pharmaceutical Biology* **2016**, *54*, 2168–2175.

(S10) Wang, Z.; Pal, D.; Patel, A.; Kwatra, D.; Mitra, A. K. Influence of Overexpression of Efflux Proteins on the Function and Gene Expression of Endogenous Peptide Transporters in MDR-transfected MDCKII Cell Lines. *International Journal of Pharmaceutics* **2013**, *441*, 40–49.

(S11) Harwood, M. D.; Zettl, K.; Weinheimer, M.; Pilla-Reddy, V.; Shen, H.; Jacobs, F.; Chu, X.; Huth, F.; Nakakariya, M.; Chothe, P. P.; Neuhoff, S.; Wiśniewski, J. R. Interlaboratory Variability in the Madin–Darby Canine Kidney Cell Proteome. *Molecular Pharmaceutics* **2023**, *20*, 3505–3518.

(S12) Pevear, D. C.; Tull, T. M.; Seipel, M. E.; Groarke, J. M. Activity of Pleconaril against Enteroviruses. *Antimicrobial Agents and Chemotherapy* **1999**, *43*, 2109–2115.

(S13) Rogers, J. M.; Diana, G. D.; McKinlay, M. A. In *Antiviral Chemotherapy 5*; Mills, J., Volberding, P. A., Corey, L., Eds.; Springer US: Boston, MA, 1999; Vol. 458; pp 69–76.

(S14) Romero, J. R. Pleconaril: A Novel Antipicornaviral Drug. *Expert Opinion on Investigational Drugs* **2001**, *10*, 369–379.

(S15) Egorova, A.; Kazakova, E.; Jahn, B.; Ekins, S.; Makarov, V.; Schmidtke, M. Novel Pleconaril Derivatives: Influence of Substituents in the Isoxazole and Phenyl Rings on the Antiviral Activity against Enteroviruses. *European Journal of Medicinal Chemistry* **2020**, *188*, 112007.

(S16) Lane, T. R.; Fu, J.; Sherry, B.; Tarbet, B.; Hurst, B. L.; Riabova, O.; Kazakova, E.; Egorova, A.; Clarke, P.; Leser, J. S.; Frost, J.; Rudy, M.; Tyler, K. L.; Klose, T.; Volobueva, A. S.; Belyaevskaya, S. V.; Zarubaev, V. V.; Kuhn, R. J.; Makarov, V.; Ekins, S. Efficacy of an Isoxazole-3-Carboxamide Analog of Pleconaril in Mouse Models of Enterovirus-D68 and Coxsackie B5. *Antiviral Research* **2023**, *216*, 105654.

(S17) Rhoden, E.; Zhang, M.; Nix, W. A.; Oberste, M. S. *In Vitro* Efficacy of Antiviral Compounds against Enterovirus D68. *Antimicrobial Agents and Chemotherapy* **2015**, *59*, 7779–7781.

(S18) Sun, L.; Meijer, A.; Froeyen, M.; Zhang, L.; Thibaut, H. J.; Baggen, J.; George, S.; Vernachio, J.; Van Kuppeveld, F. J. M.; Leyssen, P.; Hilgenfeld, R.; Neyts, J.; Delang, L. Antiviral Activity of Broad-Spectrum and Enterovirus-Specific Inhibitors against Clinical Isolates of Enterovirus D68. *Antimicrobial Agents and Chemotherapy* **2015**, *59*, 7782–7785.

(S19) Aviragen Antiviral Drug Vapendavir Fails Phase IIb Study in Asthma Patients. https://www.genengnews.com/news/aviragen-antiviral-drug-vapendavir-fails-phase-iib-study-in-asthma-patients/.

(S20) Feil, S. C.; Hamilton, S.; Krippner, G. Y.; Lin, B.; Luttick, A.; McConnell, D. B.; Nearn, R.; Parker, M. W.; Ryan, J.; Stanislawski, P. C.; Tucker, S. P.; Watson, K. G.; Morton, C. J. An Orally Available 3-Ethoxybenzisoxazole Capsid Binder with Clinical Activity against Human Rhinovirus. *ACS Medicinal Chemistry Letters* **2012**, *3*, 303–307.

(S21) Andries, K.; Dewindt, B.; Snoeks, J.; Willebrords, R.; Van Eemeren, K.; Stokbroekx, R.; Janssen, P. A. In Vitro Activity of Pirodavir (R 77975), a Substituted Phenoxy-Pyridazinamine with Broad-Spectrum Antipicornaviral Activity. *Antimicrobial Agents and Chemotherapy* **1992**, *36*, 100–107.

(S22) Badger, J.; Minor, I.; Oliveira, M. A.; Smith, T. J.; Rossmann, M. G. Structural Analysis of Antiviral Agents That Interact with the Capsid of Human Rhinoviruses. *Proteins: Structure, Function, and Bioinformatics* **1989**, *6*, 1–19.
